# Synthesis and Anti-Proliferative Evaluation of Arctigenin Analogues with C-9′ Derivatisation

**DOI:** 10.3390/ijms24021167

**Published:** 2023-01-06

**Authors:** Emily K. Paulin, Euphemia Leung, Lisa I. Pilkington, David Barker

**Affiliations:** 1 School of Chemical Sciences, University of Auckland, Auckland 1010, New Zealand; 2 The MacDiarmid Institute for Advanced Materials and Nanotechnology, Wellington 6012, New Zealand; 3 Auckland Cancer Society Research Centre, University of Auckland, Auckland 1023, New Zealand; 4 Department of Molecular Medicine and Pathology, University of Auckland, Auckland 1023, New Zealand

**Keywords:** lignans, dibenzylbutyrolactone, arctigenin, prodrugs, anti-proliferative

## Abstract

Dibenzylbutyrolactone lignans (DBLs) are a class of natural products with a wide variety of biological activities. Due to their potential for the development of human therapeutic agents, DBLs have been subjected to various SAR studies in order to optimise activity. Previous reports have mainly considered changes on the aromatic rings and at the benzylic carbons of the compounds, whilst the effects of substituents in the lactone, at the C-9′ position, have been relatively unexplored. This position has an unexploited potential for the development of novel dibenzyl butyrolactone derivatives, with previous preliminary findings revealing C-9′-hydroxymethyl analogues inducing programmed cell cycle death. Using the core structure of the bioactive natural product arctigenin, C-9′ derivatives were synthesised using various synthetic pathways and with prepared derivatives providing more potent anti-proliferative activity than the C-9′-hydroxymethyl lead compound.

## 1. Introduction

Lignans are a large natural product class of structurally and functionally diverse phenylpropanoids, isolated from over 70 known families of plants worldwide [1,2,3,4]. The lignan framework is derived from the oxidative dimerisation of two phenylpropane (C-6–C-3) moieties, which produces a linkage between each monomer’s propyl side chains at the respective C-8 carbons [1,5].

**Figure 1:**
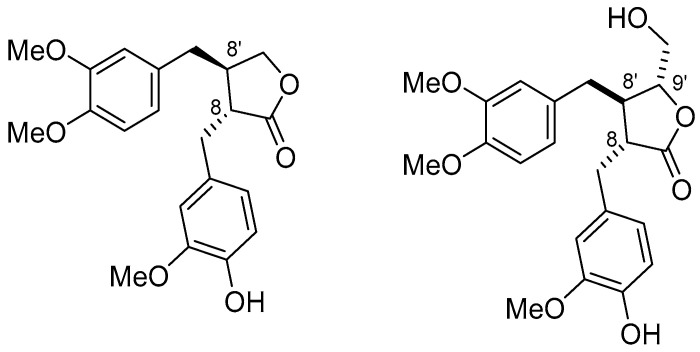
Arctigenin (**left**) and racemic C-9′ methylenehydroxy analogue (**right**).

Arctigenin is a natural product belonging to the dibenzylbutyrolactone subclass of lignans (Figure 1). These structures comprise a γ-lactone core, with dibenzyl substitution at the C-8 and C-8’ positions in an anti-relationship [6,7]. Arctigenin possesses a range of biological activities and consequently has been well-studied to determine structure-activity relationships of its derivatives [6,7,8,9,10,11,12]. Results from previous studies have confirmed the lignan’s biological activities, with arctigenin analogues having cytotoxic, anti-tumour and hypoglycaemic activities, amongst others [10,12,13,14]. Many of these derivatives have explored modifications of the aromatic rings, and to a slightly lesser extent the benzylic positions, [9,11,13,15,16], but there is a large underrepresentation of lactone ring modifications.

To date, only one synthetic derivative of arctigenin with C-9′ modification has been reported—a compound containing a methylenehydroxy group at C-5 in the lactone ring (C-9′ according to lignan nomenclature, Figure 1 right) [17,18]. This compound showed the induction of apoptosis in Jurkat T cells with only 2% necrosis. This derivative was accessed using an acyl-Claisen rearrangement as the key step to establish the necessary *trans* relationship between C-8 and C-8’ groups. The prolific activities of arctigenin and lack of SAR information at the C-9′ position inspired this work to synthesise additional C-9′ analogues. Herein, we report the synthesis of 15 arctigenin derivatives with different C-9′ substitution, and their anti-proliferative activities.

## 2. Results and Discussion

### 2.1. Retrosynthetic Analysis of C-9′ Arctigenin Analogues

**Scheme 1:**
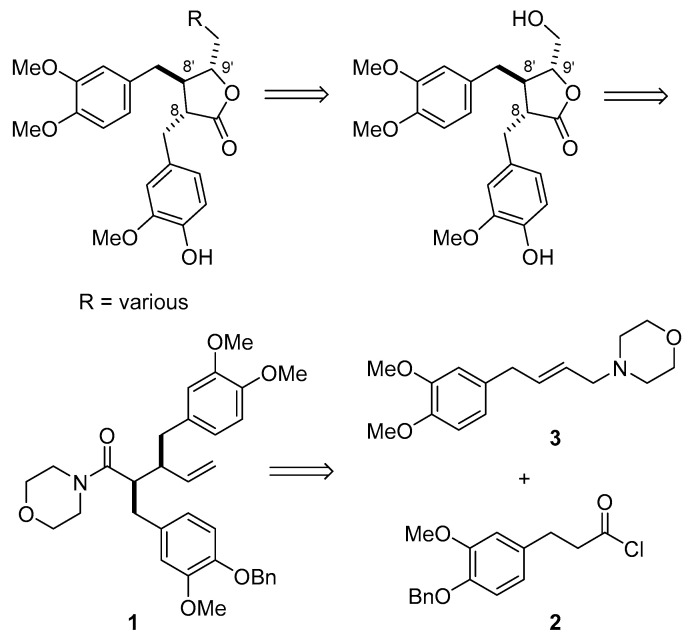
Retrosynthetic approach to racemic C-9′ analogues.

The proposed pathway to access the targeted arctigenin analogues also exploited an acyl-Claisen rearrangement [17] to introduce the correct relative stereochemistry between benzyl groups in the morpholine pentenamide **1** and converged two parallel pathways (Scheme 1). Cyclisation of the rearrangement product was envisaged to establish the core DBL lactone framework and included the C-9′ substitution of a methylenehydroxy group, from which derivatisations could be prepared. The acyl-Claisen precursors, an acid chloride **2** and allylic morpholine **3**, could be prepared from vanillin **4** and 4-allyl-1,2-dimethoxybenzene **5**, respectively, through two separate routes.

### 2.2. Synthesis of Acid Chloride ***2***

**Scheme 2:**
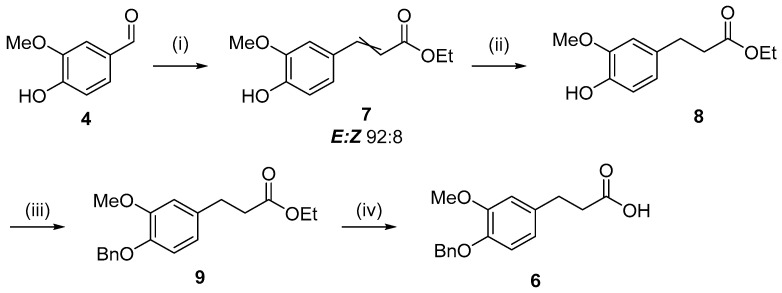
Synthesis of acid **6**. Reagents and conditions: (**i**) Ph_3_PCHCO_2_Et (1.1 equiv.), CH_2_Cl_2_, rt, 21 h, **7** 80%; (**ii**) H_2_, Pd/C (10% *w*/*w*), EtOAc, rt, 20 h, **8** quant.; (**iii**) BnBr (3 equiv.), K_2_CO_3_ (3 equiv.), MeCN, 80 °C, 46 h, **9** 93%; (**iv**) NaOH (4.5 equiv.), MeOH, rt, 2.5 h, **6** 92%.

Acid chloride **2** was prepared when required from the more stable carboxylic acid **6**. Synthesis of **6** began from vanillin **4**, which was subjected to a Wittig olefination with (carbethoxymethylene)triphenylphosphorane, following literature methods [17] to give α,β-unsaturated ethyl ester **7** in 80% yield (*E*:*Z*, 92:8). The newly installed alkene was reduced by catalytic hydrogenation to give saturated ester **8** in quantitative yield (Scheme 2). With the saturated ester **8** in hand, the phenol substituent was protected as the benzyl ether to give **9** in 93% yield. Hydrolysis of the ester **9** gave carboxylic acid **6** in 92% yield and the resulting acid chloride was prepared in situ at the time of the successive step due to its instability.

### 2.3. Synthesis of Allylic Morpholine ***3***

**Scheme 3:**
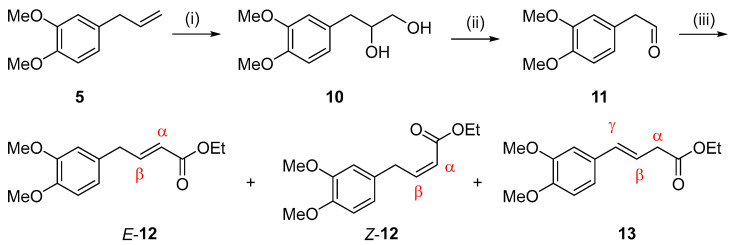
Synthesis of allylic ester **12**. Reagents and conditions: (**i**) OsO_4_ (2.5 mol-%), NMO (3 equiv.), *t*-BuOH/H_2_O (1:1), rt, 4 days, **10** 94%; (**ii**) NaIO_4_ (1.2 equiv.), MeOH/H_2_O (3:1), rt, 3 days, **11** 97%; (**iii**) (EtO)_2_POCH_2_CO_2_Et (1.5 equiv.), NaH (2 equiv.), THF, 0 °C to rt, 18 h, *E*-**12**, 39%, *Z*-**12** 2%, **13** 7%.

To obtain allylic morpholine **3**, 4-allyl-1,2-dimethoxybenzene **5** was first dehydroxylated, under Upjohn conditions [19], to give diol **10** in 94% yield (Scheme 3). The newly formed diol moiety then underwent oxidative cleavage using NaIO_4_ [17], to afford aldehyde **11** in 97% yield, which was used immediately, due to its tendency to degrade even stored at low temperatures. Thus, **11** underwent a Horner-Wadsworth-Emmons (HWE) reaction, forming the respective (*E*)-α,β-unsaturated ethyl ester **12** in 73% yield [20,21,22]. During repeated syntheses of α,β-unsaturated ester **12**, the *E*-selectivity of the Horner-Wadsworth-Emmons reaction was found to vary with the formation of both the *Z*-isomer and a third regioisomer, which was determined to be a β,γ-unsaturated ester **13** (Scheme 3). Separation of the desired *E*-isomer **12** on AgNO_3_-treated silica was possible but poor, leading to diminished yields.

### 2.4. Prevention of Isomeric Esters

Only *E*-isomer *E*-**12** was required for further steps, therefore, in order to prevent double bond migration to form **13**, different conditions were trialled for the formation of allylic ester **12**. While the mechanism of rearrangement was not confirmed, it was proposed to be base-mediated, through abstraction of the γ-proton after formation of the initial α,β-unsaturated product (Scheme 3), with the resulting β,γ-unsaturated product **13** stabilised by increased conjugation. As a result, different bases were screened. The migrated isomer was observed to a lesser extent under kinetic control or with the use of hindered bases, such as DBU under Masamune-Roush conditions [23], but unfortunately, these reactions still had poor *E*/*Z* stereocontrol. After reports of MeMgBr use to suppress isomerisation in PhCH_2_CHO aldehydes, this was applied as a base in the HWE reaction between triethylphosphonoacetate and aldehyde **11** [24]. As a result, neither the migrated species **13** nor *Z*-isomer *Z*-**12** were observed. On a larger scale, good selectivity was maintained (9:1 *E*:*Z*), but unfortunately, the yield was poor (10%), so the exploration of other methods was resumed.

### 2.5. Grubbs Cross Metathesis Pathway; Revised Route to ***3***

An alternative route which did not involve a HWE reaction was then developed by implementing a cross metathesis [25] approach between 4-allyl-1,2-dimethoxybenzene **5** and ethyl acrylate. Using Grubb’s second-generation catalyst at a loading of 5 mol-% and three equivalents of ethyl acrylate, full conversion to the *E* product *E*-**12** took place in 91% yield. No migrated product **13** was observed, allowing large scale synthesis of ester *E*-**12**.

**Scheme 4:**
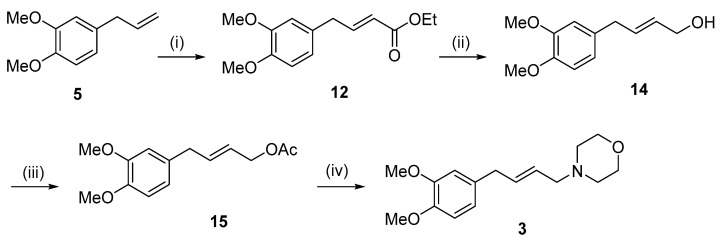
Synthetic pathway to allylic morpholine **3** using cross metathesis approach. Reagents and conditions: (**i**) Ethyl acrylate (3 equiv.), Grubbs II (1.9 mol-%), CH_2_Cl_2_, rt, 24 h, **12** 91%; (**ii**) DIBAL-H (2.9 equiv.), PhMe, −10 °C to rt, 20 h, **14** quant.; (**iii**) Ac_2_O (2.2 equiv.), Et_3_N (3 equiv.), DMAP (10 mol-%), CH_2_Cl_2_, 0 °C to rt, 25 h, **15**; (**iv**) morpholine (1.9 equiv.), Pd(PPh_3_)_4_ (5 mol-%), THF, reflux, 5 days, **3** 83% (two steps).

Ester *E*-**12** was then fully reduced to primary allylic alcohol **14** using DIBAL-H (Scheme 4). The final step towards allylic morpholine **3** involved substitution of the hydroxyl group in **14** for a morpholine moiety. The reaction was attempted using various strategies, including via mesylation, tosylation and bromination with all giving the desired product **3**, but in poor yields.

In an alternate approach, acetate **15** was then synthesised from alcohol **14**, then subjected to Tsuji-Trost allylation conditions, using palladium tetrakis Pd(PPh_3_)_4_ and morpholine. With the possibility of two regioisomers of the allylic amine product, thermodynamic control was implemented to ensure the desired linear isomer was obtained over the possible kinetic branched product [26]. Over two steps from the allylic alcohol **14**, the desired allylic morpholine **3** was achieved in 83% yield as solely the *E*-isomer, linear product (Scheme 4).

### 2.6. Synthesis of 9’-CH_2_OH Lactones

**Scheme 5:**
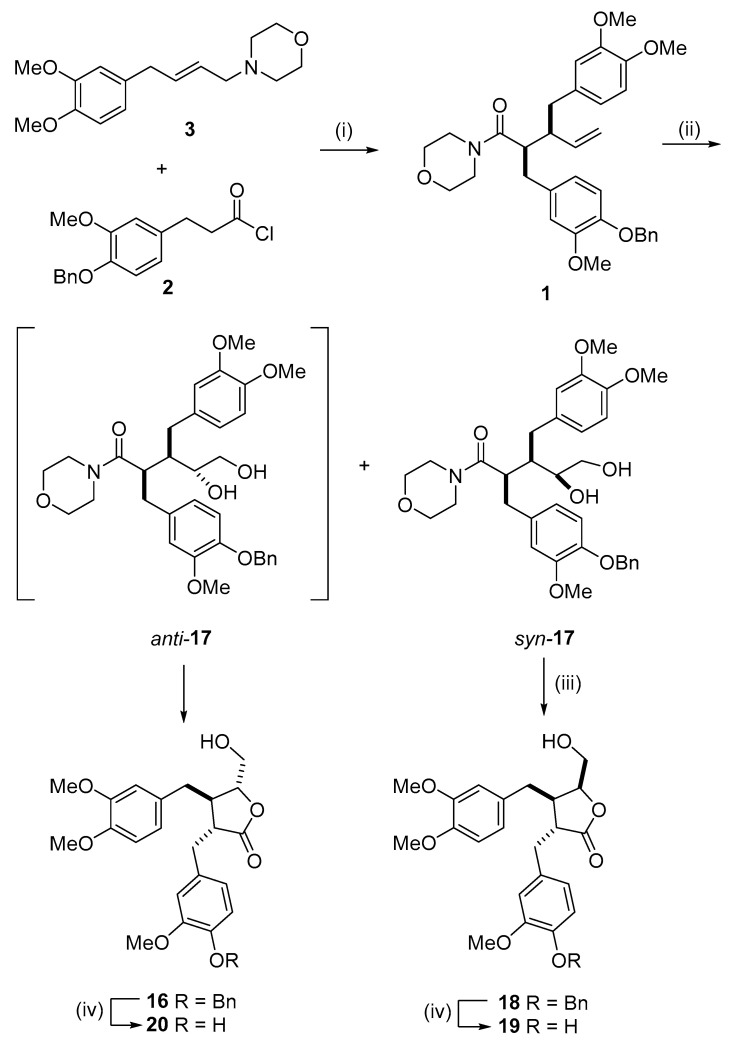
Acyl-Claisen rearrangement and cyclisation. Reagents and conditions: (**i**) TiCl_4_.2THF (1 equiv.), *i*-Pr_2_NEt (1.5 equiv.), CH_2_Cl_2_, rt, 24 h, **1** 85%; (**ii**) OsO_4_ (2.5 mol-%), NMO (3 equiv.), *t*-BuOH/H_2_O (1:1), rt, 4 days, *syn*-**17** 12%, **16** 88%; (**iii**) 2M HCl, MeOH, reflux, 4 h, **18** 94%; (**iv**) Pd/C (10% *w*/*w*), H_2_, EtOAc, rt, 19 h, **19** 56%. **20** quant.

With **3** and **6** prepared, acid **6** was then converted to acid chloride **2** in situ using oxalyl chloride, before undergoing a TiCl_4_.2THF induced acyl-Claisen rearrangement [17,27] with (*E*)-allylic morpholine **3**. One equivalent of TiCl_4_.2THF was required, as we have previously shown acyl-Claisen rearrangements with aromatic substituents require stochiometric amounts of Lewis acid to occur [27]. The rearrangement successfully took place to give racemic morpholine amide **1** in 85% yield as a single *syn*-diastereomer. Dihydroxylation, again under Upjohn conditions, cyclised amide **1** in situ to γ-lactone **16** in 88% yield (Scheme 5). The cyclisation proceeds through a diol intermediate, from which anti-**17** spontaneously formed lactone **16**. *Syn*-diol **17** was also formed in 12% yield and did not undergo cyclisation. This allowed *syn*-**17** to be isolated and treated with 2 M HCl to give the epimeric lactone **18** with a *trans*,*cis* relationship between the C-8, C-8’ and C-9′ stereocentres. Under both sets of conditions, the respective diastereomer (**16** or **18**) was afforded as the sole product. The benzyl ether in each diastereomer was deprotected to their respective phenol, in 56% yield as *trans*,*cis* isomer **19** and *trans*,*trans* isomer **20** in quantitative yields (Scheme 5).

### 2.7. Synthesis of Lactone Derivatives

With lactone **16** successfully synthesised, a range of C-9′ functionalised derivatives were targeted to explore the effects of modifications at this position on the anti-proliferative activity.

#### 2.7.1. Ester Derivatives

**Scheme 6:**
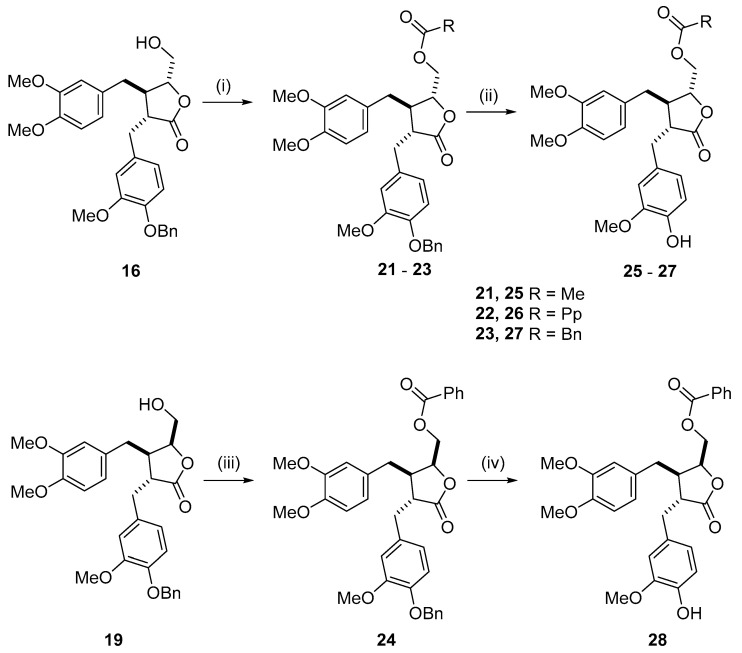
Synthesis of esters. Reagents and conditions: (**i**) Et_3_N (2 equiv.), DMAP (5 mol-%), RCOCl (1.2 equiv.), CH_2_Cl_2_, 0 °C to rt, 4–23 h, **21**–**23** 72–88%; (**ii**) Pd/C (10% *w*/*w*), H_2_, EtOAc, rt, 22–24 h, **25**–**27** (64%–quant.); (**iii**) Et_3_N (2 equiv.), DMAP (5 mol-%), BzCl (1.2 equiv.), CH_2_Cl_2_, 0 °C–rt, 21 h, **24** 48%; (**iv**) Pd/C (10% *w*/*w*), H_2_, EtOAc, rt, 19 h, **28** quant.

A series of ester derivatives was completed to add prodrug-like groups, capable of being cleaved by cellular esterases [28]. Differing ester groups were installed using the addition of acid chlorides to lactone **16** with Et_3_N and catalytic DMAP. Differing chain lengths and aromaticity were accessed with the synthesis of acetate **21**, propionate **22**, and benzoate **23** esters (Scheme 6). An additional benzoate ester **24** was synthesised from isomer **19**, in 48% yield, to provide an example of the epimeric *trans*,*cis* form. Following derivatisation of the 9’-CH_2_OH functionality, the benzylic ether at C-4 was removed via catalytic hydrogenation to give the free phenol, a directly comparable analogue of arctigenin. The synthesis of **21**–**24** was achieved in yields ranging from 72–88%. Deprotection of the benzyl ether in all the ester analogues gave C-4 phenols **25**–**27**, in yields of 64% to quantitative (Scheme 6).

#### 2.7.2. Azido Derivatives

In addition to the obtained ester derivatives, it was decided to install a triazole scaffold due to its presence in clinically used drugs, with a range of pharmacological activities and ability to enhance solubility [29,30,31]. As a preliminary example of triazole derivatised arctigenin, the simple 4-phenyl-1*H*-1,2,3-triazole moiety was accessed through a copper-catalysed azide-alkyne 1,3-dipolar cycloaddition (CuAAC) click reaction between azide-containing arctigenin analogue **29** and phenylacetylene.

**Scheme 7:**
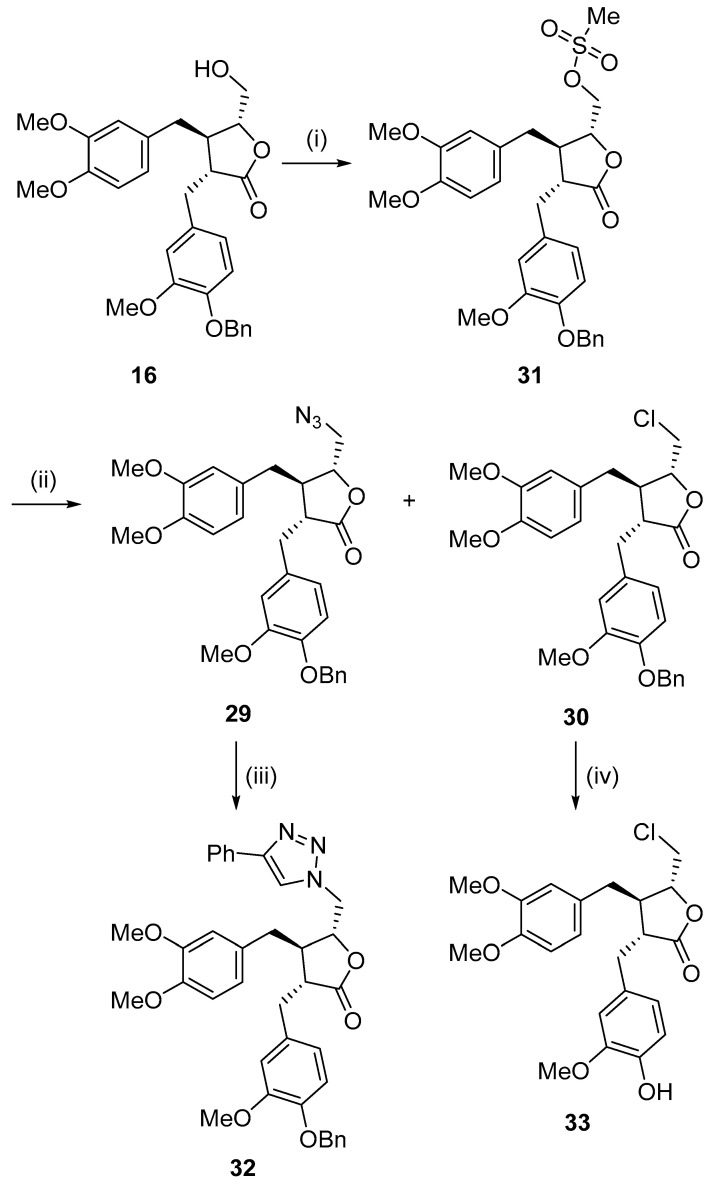
Synthesis of nitrogen-containing derivatives. Reagents and conditions: (**i**) MsCl (1.5 equiv.), Et_3_N (1.4 equiv.), CH_2_Cl_2_, 0 °C to rt, 2.5 h, **31**; (**ii**) NaN_3_ (3.8 equiv.), DMF, 85–100 °C, 27 h, **29** 78% (two steps) and **30** 25% (two steps); (**iii**) phenylacetylene (1.5 equiv.), Cu_2_SO_4_.5H_2_O (0.16 equiv.), sodium L-ascorbate (0.2 equiv.), MeCN, rt, 4 days, **32** 74%; (**iv**) Pd/C (10% *w*/*w*), H_2_, EtOAc, rt, 5 days, **33** 29%.

Azide **29** was afforded through a two-step approach of mesylation and subsequent displacement using sodium azide, affording azide **29** in 78% yield over two steps (Scheme 7). This process also saw formation of a minor alkyl chloride side product **30** through halide displacement.

The reaction of azide **29** and phenylacetylene was achieved using sodium ascorbate and copper sulfate pentahydrate in acetonitrile and after four days obtained the 1,4-disubstituted triazole **32** in 74% yield (Scheme 7).

Chloride **30** was stable to catalytic hydrogenation conditions and the benzyl ether removed cleanly through hydrogenolysis to give **33** in 29% yield. However, hydrogenation of triazole **32** was found to be surprisingly difficult and the desired phenol could not be obtained even after using a variety of conditions.

### 2.8. Anti-Proliferative Activity

Following the synthesis of the arctigenin derivatives, their anti-proliferative activities were evaluated against colorectal cancer HCT-116 and triple negative breast cancer MDA-MB-231 cell lines (Table 1). Arctigenin has previously been shown to affect the growth of MDA-MB-231 [32] whilst the HCT-116 has been used for the anti-proliferative assessment of polyphenolic natural products [33,34]. All of the compounds showed improved activity in the HCT-116 cell line over MDA-MB-231 cells. Five of the fifteen compounds had better activity than the previously prepared hydroxylmethylene derivative **16** in the MDA-MB-231 cell line, and 10 out of the 15 compounds had better activity than that reported for arctigenin itself [32]. It was found that compounds with aromatic benzylic ethers tended to outperform their phenol counterparts, and *trans*,*trans* stereochemistry between C-8, C-8’ and C-9′ was favourable over a *trans*,*cis* relationship. The most potent four compounds, based upon their ability to inhibit cell growth at 10 µM, were the same across both cell lines; **24**, **29**, **32**, and **33**, and their IC_50_ values were determined. All of the tested compounds, **24**, **29**, **32** and **33** produced similar inhibition–with mean IC_50_ values ranging between 5.79–7.45 µM (MDA-MB-231) and 3.27–6.10 µM (HCT-116) (Table 2).

## 3. Materials and Methods

### 3.1. Synthesis

General experimental details: All reactions were carried out under an inert atmosphere using distilled anhydrous solvents unless otherwise specified. Triethylamine and diisopropylethylamine were each distilled and stored over activated 4 Å Molecular Sieves. All NMR spectra were recorded on a Bruker Avance DRX 400 MHZ spectrometer at ambient temperature. Chemical shifts are reported relative to the solvent peak of CDCl_3_ (δ 7.26 for ^1^H and δ 77.16 for ^13^C) or DMSO (δ 2.50 for ^1^H and δ 39.52 for ^13^C). ^1^H NMR data are reported as position (δ), relative integral, multiplicity (s, singlet; d, doublet; dd, doublet of doublets; ddd, doublet of doublet of doublets; dt, doublet of triplets; dq, doublet of quartets; t, triplet; td, triplet of doublets; q, quartet; m, multiplet), coupling constant (*J*, Hz), and the assignment of the atom. Proton-decoupled ^13^C NMR data are reported as position (δ) and assignment of the atom. NMR assignments were performed using HMBC, COSY and HSQC, experiments. ^1^H and ^13^C NMR spectra for all precursor and final compounds (Figures S1–S45) are found in the supplementary material. The numbering of arctigenin analogues was done according to lignan nomenclature, with the two C-6-C-3 units numbered 1–9 and 1’–9’ [18]. All melting points for solid compounds are given in degrees Celsius (°C), were measured using a Reicher-Kofler block, and are uncorrected. A Perkin-Elmer Spectrum 1000 series Fourier Transform Infrared ATR spectrometer was used to record infrared spectra. Absorption maxima are expressed in wavenumbers (cm^−1^). High-resolution mass spectroscopy (HRMS) was carried out by electrospray ionisation (ESI^+^) on a MicroTOF-Q II mass spectrometer. Fétizon’s reagent was prepared following a literature procedure [35]. Unless noted, chemical reagents were used as purchased. General procedures, synthetic experimental methods, and full characterisation data (including copies of NMR spectra for all synthesised final compounds) can be found in the Supplementary Materials.

### 3.2. Cell Proliferation Assays

The synthesised arctigenin derivatives were measured for anti-proliferative activity against colorectal cancer HCT-116 and triple negative breast cancer MDA-MB-231 cell lines using ^3^H-thymidine incorporation assays. Cell lines were purchased from the American Type Culture Collection (ATCC). The anti-proliferative assays were conducted according to our previously reported methods. [36,37,38]. In short, cells were seeded in 96 well plates with 3000 cells per well and incubated with 10 µM of arctigenin-derived compounds for three days. An amount of 0.04 μCi of ^3^H-thymidine was added per well and incubated for 5 h before cells were harvested and counted. All experiments were performed in duplicate wells on separate plates with three repeats. The percentage of cells which showed incorporation of ^3^H-thymidine into the DNA relative to the control samples directly measured the cell proliferation. Two known previously active compounds were used as positive controls [39], alongside a negative control, with no compounded added.

## 4. Conclusions

In this study, fifteen novel C-9′ derivatives of arctigenin with C-9′ substitution were successfully synthesised, and analysed for their anti-proliferative activities, for the first time. This study demonstrated the use of acyl-Claisen rearrangement as an effective method to access a dibenzylbutyrolactone framework as a single *trans* diastereomer between C-8 and C-8’ and to provide C-9′ analogues with a *trans*,*trans* C-8, C-8’, C-9′ configuration. The benefits of this divergent strategy are evident through the preparation of these derivatives. Anti-proliferative testing of the synthesised compounds showed IC_50_ values as low as 3.27 µM in HCT-116 (compound **32**) and 5.79 µM (compound **29**) in MDA-MB-231 cancer cell lines, which were improved over the natural product, arcitgenin, itself. These results highlight that C-9′ substitution of dibenzylbutyrolactone lignans can improve the biological activity compared to the unsubstituted natural products. Furthermore, as the C-9′ is rarely substituted in other lignan natural products, this work suggests the potential that other classes of lignans could be similarly modified to increase their biological activity.

## Figures and Tables

**Table 1:** Anti-proliferative activities when cells treated with compound (10 µM). Values are given as the average ± standard error of three experimental replicates. Most active compounds, selected for IC_50_ determination, in highlighted rows.

Compound	Relative Thymidine Uptake at 10 µM (%)	
	HCT-116	MDA-MB-231
**16**	42.70 ± 1.47	54.48 ± 2.17
**18**	67.44 ± 8.16	86.62 ±3.64
**19**	90.01 ± 0.97	100.40 ± 2.66
**20**	87.83 ± 1.20	92.22 ± 2.66
**21**	48.92 ± 1.48	60.60 ± 1.68
**22**	44.64 ± 1.08	50.69 ± 2.37
**23**	42.31 ± 0.93	60.45 ± 0.47
**24**	34.80 ± 0.15	44.15 ± 1.78
**25**	86.01 ± 0.65	104.00 ± 3.48
**26**	90.38 ± 2.00	105.19 ± 5.09
**27**	55.86 ± 0.86	75.04 ± 3.06
**29**	23.72 ± 1.54	36.53 ± 1.32
**30**	76.48 ± 3.97	96.57 ± 2.88
**32**	28.02 ± 2.17	44.80 ± 0.37
**33**	16.48 ± 0.29	34.09 ± 1.58
Arctigenin *	-	84.81 ± 5.96 [32]

* = determined using MTT assay, after 24 h [32].

**Table 2:** IC_50_ values. Values are given as the average ± standard error of three experimental replicates. Most active compounds in highlighted rows.

Compound	Mean IC_50_ ± Standard Error (µM)	
	HCT-116	MDA-MB-231
**24**	6.10 ± 1.53	6.90 ± 0.10
**29**	5.17 ± 1.13	6.23 ± 0.63
**32**	3.27 ± 1.13	6.89 ± 0.20
**33**	5.29 ± 1.17	7.45 ± 0.70

## Data Availability

Data are contained within the article.

## Supplementary Materials

The following supporting information can be downloaded at: https://www.mdpi.com/article/10.3390/ijms24021167/s1. Synthetic procedures for all novel compounds, ^1^H and ^13^C NMR spectra for all precursor and final compounds (Figures S1–S45). References [17,40,41,42,43,44,45,46,47,48,49] are cited in supplementary materials.

## Sample Availability

Samples of the compounds are available from the authors.
