# Supplementary material for: Synthesis and Anti-Proliferative Evaluation of Arctigenin Analogues with C-9′ Derivatisation
Source: Int J Mol Sci. 2023 Jan 6;24(2):1167. doi: 10.3390/ijms24021167 (PMC9866048; doi:10.3390/ijms24021167)
Supplement: Supplementary file 1 [file ijms-24-01167-s001.zip › ijms-2114575-supplementary.pdf]

Supplementary Materials:

Synthesis and Anti-proliferative Evaluation of Arctigenin Analogues with C-9' Derivatisation

Emily K. Paulin[a,c], Euphemia Leung[b], Lisa I. Pilkington[a], and David Barker[a,c]*

[a] School of Chemical Sciences, University of Auckland, Auckland 1010, New Zealand

[b] Auckland Cancer Society Research Centre and Department of Molecular Medicine and Pathology, University of Auckland, 1142 Auckland, New Zealand.

[c] The MacDiarmid Institute for Advanced Materials and Nanotechnology, Wellington, New Zealand.

*To whom correspondence should be addressed: School of Chemical Sciences, The University of Auckland, Private Bag 92019, Auckland 1142, New Zealand.

Email: d.barker@auckland.ac.nz, Tel. 64-9-923-9703

Table of Contents:

General Synthetic Procedures: S3

Synthetic Procedures and Compound Characterisation Data..........S4

NMR spectra for precursors to compounds 3 and 6. ....S30

NMR spectra of C-9’ lactone compounds..........S43

References S75

General Synthetic Procedures:

General Procedure A: Acyl Claisen Rearrangement

To a stirred suspension of $TiCl_4$.2THF (1 mmol) in CH_2Cl_2 (5 mL) under an atmosphere of nitrogen at room temperature was added a solution of allylic morpholine (1 mmol) in CH_2Cl_2 (2.5 mL) dropwise, followed by dropwise addition of $^{i}Pr_2NEt$ (1.5 mmol) after 5 min. After stirring for 10 min a solution of acid chloride (1.2 mmol) in CH_2Cl_2 (2.5 mL) was added dropwise and the resultant mixture stirred for the specified time. The mixture was quenched with aqueous NaOH (12 mL, 1 M) and extracted with CH_2Cl_2 (3 × 10 mL). The combined organic extracts were washed with brine (6 mL), dried ($MgSO_4$) and the solvent removed *in vacuo*. The crude product was purified by flash chromatography to give the amide product.

General Procedure B: Dihydroxylation

To a stirred solution of morpholine pentenamide (1 mmol) in $^{t}BuOH/H_2O$ (1:1, 20 mL) was added NMO (3 mmol). A solution of OsO_4 (0.08 mmol, 2.5% w/v in $^{t}BuOH$) was added dropwise and the mixture stirred for the specified time. The mixture was quenched with saturated aqueous Na_2SO_3 (30 mL) and stirred for an additional hour then extracted with ethyl acetate (3 × 20 mL). The combined organic extracts were washed with aqueous KOH (5 mL, 1 M), dried ($MgSO_4$) and the solvent removed *in vacuo*. The crude product was purified by flash chromatography.

General Procedure C: Esterification

To a solution of alcohol (1 mmol) in CH_2Cl_2 (3 mL) under nitrogen at 0 °C was added DMAP (0.05 mmol) and triethylamine (2 mmol), followed by acid chloride (1.2 mmol) dropwise. The resulting mixture was warmed to rt and stirred for the specified time. After completion, the reaction was quenched with NH_4Cl (3 mL), extracted with CH_2Cl_2 (3 × 5 mL), washed with $NaHCO_3$, brine and dried (Na_2SO_4). The solvent was removed *in vacuo* and the crude product was purified by flash chromatography.

General Procedure D: Benzyl Deprotection

To a stirred solution of benzyl ether (1 mmol) in ethyl acetate (5 mL) was added 10% palladium on activated carbon (20% w/w). The solution was stirred at rt under an atmosphere of hydrogen for the specified time. The reaction mixture was filtered through celite, washed with ethyl acetate and the solvent was then removed in vacuo. The crude product was purified by flash chromatography if necessary to give the deprotected alcohol.

Synthetic Procedures and Compound Characterisation Data

Ethyl-3'-(4-hydroxy-3-methoxyphenyl)acrylate (7)

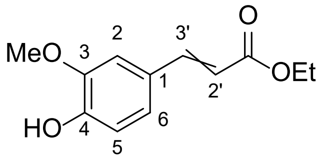

To a solution of vanillin **4** (2.0 g, 13.1 mmol) in CH_2Cl_2 (50 mL) was added (carbethoxymethylene)triphenylphosphorane (5.06 g, 14.5 mmol) and the resulting mixture stirred at rt for 21 h. The solvent was then removed *in vacuo* and the crude product purified using flash chromatography (2:1 petroleum ether, ethyl acetate) to give the *title compound* **7** (2.35 g, 80%) in a 92:8 mixture of *E*:*Z* isomers as a yellow oil.

R$_f$ = 0.41, 0.33 (*Z*-**4**, *E*-**4**, 4:1 petroleum ether, ethyl acetate);

δ_H (400 MHz; $CDCl_3$) 1.33 (3H, t, *J* = 7.1 Hz, OEtC$\underline{H_3}$), 3.91 (3H, s, 3-OCH_3), 4.25 (2H, q, *J* = 7.1 Hz, OEtC$\underline{H_2}$), 5.98 (1H, s, OH), 6.28 (1H, d, *J* = 16.0 Hz, H-3'), 6.91 (1H, d, *J* = 8.0 Hz, H-5), 7.02 (1H, d, *J* = 2.0 Hz, H-2), 7.06 (1H, dd, *J* = 8.0, 2.0 Hz, H-6), 7.61 (1H, d, *J* = 16.0 Hz, H-2').

The obtained spectroscopic data was in agreement with literature values.[1]

Ethyl 3'-(4-hydroxy-3-methoxyphenyl)propanoate (8)

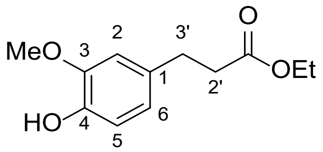

To a solution of α,β-unsaturated ester **7** (1.01 g, 3.20 mmol) in EtOAc (30 mL) was added 10% palladium on activated carbon (0.102 g, 10% w/w). The reaction mixture was stirred under an atmosphere of hydrogen for 21 h, then filtered through Celite with EtOAc and the solvent removed *in vacuo*. The crude product was purified using flash chromatography (4:1 petroleum ether, ethyl acetate) to give the *title compound* **8** (0.602 g, 84%) as a colourless oil.

R$_f$ = 0.45 (4:1 petroleum ether, ethyl acetate);

δ$_H$ (400 MHz; $CDCl_3$) 1.24 (3H, t, *J* = 7.2 Hz, $OCH_2C\underline{H_3}$), 2.59 (2H, t, *J* = 7.8 Hz, H-2'), 2.88 (2H, t, *J* = 7.8 Hz, H-3'), 3.86 (3H, s, 3-OCH_3), 4.13 (2H, q, *J* = 7.2 Hz, $OC\underline{H_2}CH_3$), 5.57 (1H, s, OH), 6.68 (1H, d, *J* = 2.0 Hz, H-6), 6.70 (1H, dd, *J* = 8.0, 2.0 Hz, H-2), 6.82 (1H, d, *J* = 8.0 Hz, H-5);

δ$_C$ (100 MHz; $CDCl_3$) 14.3 ($OCH_2\underline{C}H_3$), 30.8 (C-3'), 36.5 (C-2'), 56.0 (3-OCH_3), 60.5 ($OC\underline{H_2}CH_3$), 111.1 (C-2), 114.5 (C-5), 121.0 (C-6), 132.6 (C-1), 144.1 (C-4), 146.5 (C-3), 173.1 (C=O).

The obtained spectroscopic data was in agreement with literature values.[2,3]

Ethyl 3'-(4-(benzyloxy)-3-methoxyphenyl)propanoate (9)

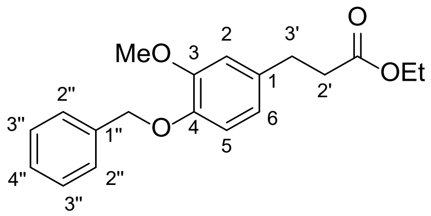

To a solution of ester **8** (0.576 g, 2.57 mmol) in MeCN (20 mL) was added K_2CO_3 (1.08g, 7.81 mmol) and stirred for 10 min, followed by the addition of BnBr (0.9 mL, 7.77 mmol). The resulting mixture was stirred for 46 h, then quenched with water (10 mL) and extracted with CH_2Cl_2 (3 × 10 mL). The organic phases were combined, washed with water (2 × 10 mL) and dried ($MgSO_4$). The solvent was removed *in vacuo* and the crude product purified using flash chromatography (9:1 petroleum ether, ethyl acetate) to give the *title compound* **9** (0.750 g, 93%) as a colourless solid. **m.p.** 28–30 °C.

R$_f$ = 0.37 (9:1 petroleum ether, ethyl acetate);

δ$_H$ (400 MHz; $CDCl_3$) 1.23 (3H, t, *J* = 7.1 Hz, $OCH_2C\underline{H_3}$), 2.59 (2H, t, *J* = 7.8 Hz, H-2'), 2.89 (2H, t, *J* = 7.8 Hz, H-3'), 3.87 (3H, s, 3-OCH_3), 4.13 (2H, q, *J* = 7.1 Hz, $OC\underline{H_2}CH_3$), 5.12 (2H, s, 1''-$C\underline{H_2}$), 6.67 (1H, dd, *J* = 8.0, 2.0 Hz, H-6), 6.76 (1H, d, *J* = 2.0 Hz, H-2), 6.80 (1H, d, *J* = 8.0 Hz, H-5), 7.29 (1H, t, *J* = 7.1 Hz, H-4''), 7.36 (1H, t, *J* = 7.1 Hz, H-3''), 7.43 (1H, d, *J* = 7.1 Hz, H-2'');

δ$_C$ (100 MHz; $CDCl_3$) 14.4 ($OCH_2\underline{C}H_3$), 30.8 (C-3'), 36.3 (C-2'), 56.1 (CH_3), 60.5 ($O\underline{C}H_2CH_3$), 71.3 (1''-$\underline{C}H_2$), 112.4 (C-2), 114.4 (C-5), 120.3 (C-6), 127.4 (C-2''), 127.9 (C-4''), 128.6 (C-3''), 134.0 (C-1), 137.5 (C-1''), 129.7 (C-3), 146.8 (C-4), 173.1 (C=O).

The obtained spectroscopic data was in agreement with literature values.[4]

3'-(4-(Benzyloxy)-3-methoxyphenyl)propanoic acid (6)

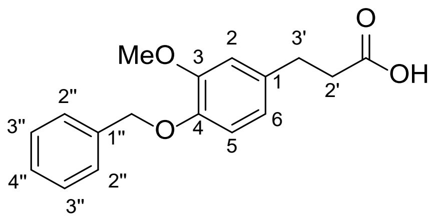

To a solution of ester **9** (0.690 g, 2.19 mmol) in MeOH (20 mL) was added aqueous NaOH (1 M, 10 mL) and stirred for 2.5 h. The mixture was then acidified with aqueous 2 M HCl, extracted with EtOAc (3 × 10 mL), dried ($MgSO_4$) and the solvent removed *in vacuo* to give the *title compound* **6** (0.574 g, 92%) as a white solid. **m.p.** 95–97 °C.

δ_H (400 MHz; $CDCl_3$) 2.65 (2H, t, *J* = 7.8 Hz, H-2'), 2.89 (2H, t, *J* = 7.8 Hz, H-3'), 3.87 (3H, s, 3-OCH_3), 5.12 (2H, s, 1''-C$\underline{H_2}$), 6.67 (1H, dd, *J* = 8.0, 2.0 Hz, H-6), 6.75 (1H, s, H-2), 6.80 (1H, d, *J* = 8.0 Hz, H-5), 7.29 (1H, t, *J* = 7.1 Hz, H-4''), 7.36 (1H, t, *J* = 7.1 Hz, H-3''), 7.45 (1H, t, *J* = 7.1 Hz, H-2'');

δ_C (100 MHz; $CDCl_3$) 30.4 (C-3'), 36.0 (C-2'), 56.1 (CH_3), 71.3 (1''-$\underline{C}H_2$), 112.4 (C-2), 114.4 (C-5), 120.2 (C-6), 127.4 (C-2''), 127.9 (C-4''), 128.6 (C-3''), 133.6 (C-1), 137.4 (C-1''), 146.9 (C-4), 149.8 (C-3), 178.9 (C=O);

IR ν_{max}(ATR)/cm^{-1}: 2909, 2881, 1690, 1514, 1389, 1229, 1141;

***m/z* (ESI$^+$)**: 309 (MNa^+, 100%); **HRMS** (ESI$^+$) Found (MNa^+): 309.1097; $C_{17}H_{18}NaO_4$ requires 309.1097.

The obtained spectroscopic data was in agreement with literature values.[5]

3'-(3,4-Dimethoxyphenyl)propane-1,2-diol (10)

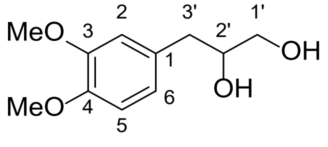

To a stirred solution of NMO (2.04 g, 17.4 mmol) in H_2O/*t*-BuOH (1:1, 30 mL) was added 4-allyl-1,2-dimethoxybenzene (1 mL, 5.84 mmol). A solution of OsO_4 (0.16 mL, 2.5% w/w in *t*-BuOH) was then added dropwise and the resulting mixture stirred at rt for 49 h. The mixture was then quenched with sat. aq. Na_2SO_3 (30 mL) and stirred for an additional 1 h. The mixture was extracted with EtOAc (3 x 15 mL), the organic layers combined, washed with aqueous KOH (1 M, 10 mL) and dried ($MgSO_4$). The solvent was removed *in vacuo* and the product was concentrated under high vacuum to give the *title compound* **10** (1.20 g, 96%) as a white solid. **m.p.** 65–67 °C.

δ$_H$ (400 MHz; $CDCl_3$) 2.07 (2H, s, OH), 2.69 (1H, dd, *J* = 13.9, 8.0 Hz, 3'-H_A), 2.77 (1H, dd, *J* = 13.9, 5.2 Hz, 3'-H_B), 3.51 (1H, dd, *J* = 11.0, 7.0 Hz, 1'-H_A), 3.68 (1H, dd, *J* = 11.0, 3.3 Hz, 1'-H_B), 3.85 (3H, s, 4-OCH_3), 3.86 (3H, s, 3-OCH_3), 3.88–3.94 (1H, m, H-2'), 6.75 (1H, s, H-2), 6.77 (1H, d, *J* = 2.0 Hz, H-6), 6.82 (1H, d, *J* = 8.0 Hz, H-5);

δ$_C$ (100 MHz; $CDCl_3$) 39.5 (C-3'), 56.0 (3-O$\underline{C}H_3$ or 4-O$\underline{C}H_3$), 56.1 (3-O$\underline{C}H_3$ or 4-O$\underline{C}H_3$), 66.2 (C-1'), 73.2 (C-2'), 111.6 (C-5), 112.6 (C-2), 121.4 (C-6), 130.3 (C-1), 148.0 (C-4), 149.2 (C-3).

The obtained spectroscopic data was in agreement with literature values.[6]

2'-(3,4-Dimethoxyphenyl)acetaldehyde (11)

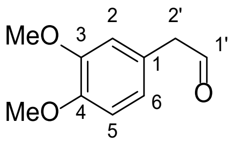

To diol **10** (0.601 g, 2.83 mmol) in MeOH/H_2O (3:1, 20 mL) was added $NaIO_4$ (0.743 g, 3.47 mmol) and the resultant mixture stirred at rt for 2 h. The reaction was quenched with the addition of brine (20 mL) and extracted with EtOAc (3 × 10 mL). The organic extracts were combined, washed with water (2 × 10 mL) and dried ($MgSO_4$). The solvent was removed *in vacuo* and the crude product purified using flash chromatography (1:1 petroleum ether: ethyl acetate) to give the *title compound* **11** (0.497 g, 97%) as a pale-yellow oil.

R$_f$ = 0.16 (1:1 petroleum ether, ethyl acetate);

δ$_H$ (400 MHz; $CDCl_3$) 3.62 (2H, d, *J* = 2.5 Hz, H-2'), 3.87 (6H, s, 3-OCH_3, 4-OCH_3), 6.70 (1H, d, *J* = 2.0 Hz, H-2), 6.77 (1H, dd, *J* = 8.0, 2.0 Hz, H-6), 6.86 (1H, d, *J* = 8.0 Hz, H-5), 9.72 (1H, t, *J* = 2.5 Hz, CHO);

δ$_C$ (100 MHz; $CDCl_3$) 50.3 (C-2'), 56.0 (3-O$\underline{C}H_3$ or 4-O$\underline{C}H_3$), 56.1 (3-O$\underline{C}H_3$ or 4-O$\underline{C}H_3$), 111.8 (C-5), 112.8 (C-2), 122.0 (C-6), 124.3 (C-1), 148.6 (C-4), 149.5 (C-3), 199.7 (CHO).

The obtained spectroscopic data was in agreement with literature values.[7]

Ethyl (*E*)-4'-(3,4-dimethoxyphenyl)but-2'-enoate (12)

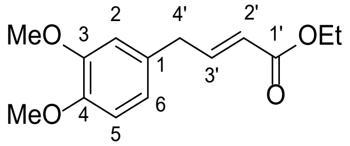

Method A: *via* Horner-Wadsworth-Emmons reaction of **11**.

Method adapted from one reported by Sun *et al.*[8]

To a suspension of NaH (0.192 g, 60% dispersion in mineral oil, 8.00 mmol) in THF (40 mL) at 0 °C was added triethylphosphonoacetate (1.2 mL, 6 mmol) dropwise and stirred for 40 min, followed by addition of aldehyde **11** (0.721 g, 4 mmol). The resultant mixture was stirred at rt for an additional 16 h then quenched with sat. aq. NH_4Cl (20 mL) and extracted with EtOAc (3 × 15 mL). The organic extracts were combined, washed with brine (10 mL) and dried (Na_2SO_4). The solvent was removed *in vacuo* and the crude product purified using flash chromatography (9:1 petroleum ether, ethyl acetate) to give the *title compound* **12** (0.733 g, 73%) as a pale-yellow oil.

Method B: *via* Grubbs cross-metathesis of **5** and ethyl acrylate.

Method adapted from one reported by Hryniewicka *et al.*[9]

To a mixture of 4-allyl-1,2-dimethoxybenzene **5** (1 mL, 5.83 mmol) and ethyl acrylate (1.90 mL, 17.5 mmol) in CH_2Cl_2 (40 mL) was added Grubbs' 2nd generation catalyst (92.8 mg, 0.109 mmol, 1.9 mol-%) in CH_2Cl_2 (2 mL). The resulting mixture was stirred at 40 °C for 23 h then the solvent removed *in vacuo* and the crude product purified using flash chromatography (19:1 to 9:1 petroleum ether, ethyl acetate) to give the *title compound* **12** (1.176 g, 81%) as a yellow oil.

R_f = 0.67 (2:1 petroleum ether, ethyl acetate);

δ_H (400 MHz; $CDCl_3$) 1.27 (3H, t, *J* = 7.1 Hz, $OCH_2C\underline{H_3}$), 3.46 (2H, dd, *J* = 6.8, 2.0 Hz, H-4'), 3.86 (3H, s, 3-OCH_3), 3.87 (3H, s, 4-OCH_3), 4.18 (2H, q, *J* = 7.1 Hz, $OC\underline{H_2}CH_3$), 5.80 (1H, dt, *J* = 15.5, 1.7 Hz, H-2'), 6.67 (1H, d, *J* = 2.0 Hz, H-2), 6.71 (1H, dd, *J* = 8.0, 2.0 Hz, H-6), 6.81 (1H, d, *J* = 8.0 Hz, H-5), 7.08 (1H, dt, *J* = 15.5, 6.8 Hz, H-3');

δ_C (100 MHz; $CDCl_3$) 14.4 ($OCH_2C\underline{H_3}$), 38.2 (C-4'), 56.0 (3-$O\underline{C}H_3$ or 4-$O\underline{C}H_3$), 56.1 (3-$O\underline{C}H_3$ or 4-$O\underline{C}H_3$), 60.4 ($OC\underline{H_2}CH_3$), 111.6 (C-5), 112.2 (C-2), 121.0 (C-6), 122.3 (C-2'), 130.4 (C-1), 147.7 (C-3'), 148.0 (C-4), 149.2 (C-3), 166.7 (C-1').

The obtained spectroscopic data was in agreement with literature values.[5]

(*E*)-4'-(3,4-Dimethoxyphenyl)but-2'-en-1'-ol (14)

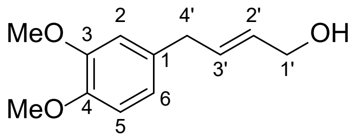

To a solution of α,β-unsaturated ester **12** (0.103 g, 0.412 mmol) in PhMe (20 mL) at −10 °C, was added DIBAL-H (1.2 mL, 1 M in PhMe) and the resulting mixture stirred for 20 h. The reaction was quenched with the addition of 2 M HCl until gas evolution ceased, the organic phase was separated, and the aqueous phase further extracted with CH_2Cl_2 (3 × 10 mL). The organic extracts were combined, washed with water (10 mL) and dried ($MgSO_4$). The solvent was removed *in vacuo* to give the *title compound* **14** (84 mg, quant.) as a yellow oil.

δ_H (400 MHz; $CDCl_3$) 3.33 (2H, d, *J* = 6.5 Hz, H-4'), 3.86 (3H, s, 4-OCH_3), 3.87 (3H, s, 3-OCH_3), 4.14 (2H, t, *J* = 5.3 Hz, H-1'), 5.67–5.74 (1H, m, H-2'), 5.82–5.89 (1H, m, H-3'), 6.70–6.74 (2H, m, H-2, H-6), 6.81 (1H, d, *J* = 8.0 Hz, H-5);

δ_C (100 MHz; $CDCl_3$) 38.4 (C-4'), 56.0 (3-OCH_3 or 4-OCH_3), 56.1 (3-OCH_3 or 4-OCH_3), 111.5 (C-5), 112.1 (C-2), 120.5 (C-6), 130.2 (C-2'), 132.0 (C-3'), 132.7 (C-1), 147.6 (C-4), 149.1 (C-3).

The obtained spectroscopic data was in agreement with literature values.[5]

(*E*)-4-(4-(Benzyloxy)-3-methoxyphenyl)but-2-en-1-yl acetate (15)

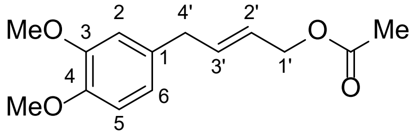

Method adapted from one reported by Parpal *et al.*[10]

To allylic alcohol **14** (68 mg, 0.239 mmol) in CH_2Cl_2 (8 mL) at 0 °C was added Et_3N (0.10 mL, 0.717 mmol) and stirred for 5 min. Ac_2O (0.05 mL, 0.529 mmol) was added dropwise and catalytic DMAP (3 mg, 10 mol-%) added. The reaction mixture was warmed to rt and stirred for 23 h, after which sat. aq. $NaHCO_3$ (5 mL) and CH_2Cl_2 (10 mL) were added, the organic layers extracted (3 × 20 mL), dried ($MgSO_4$) and solvent removed *in vacuo* to give the *title compound* **15** as a yellow oil which was used immediately in the next step without further purification.

(*E*)-1'-(4'-(3,4-Dimethoxyphenyl)but-2'-en-1'-yl)morpholine (47c), 4-(methylsulfonyl)morpholine (62) and 1,4'-bis(3,4-dimethoxyphenyl)but-2'-ene (3)

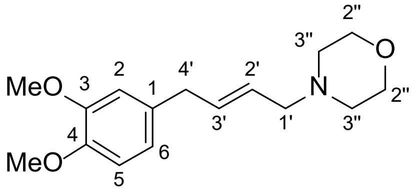

Method A: *via* mesylate intermediate.

To a solution of allylic alcohol **3** (0.311 g, 1.49 mmol) in CH_2Cl_2 (20 mL) at 0 °C was added Et_3N (0.63 mL, 1.30 mmol), followed by MsCl (0.63 mL, 4.5 mmol) after 10 min. After an additional 10 min, morpholine (0.30 mL, 3.43 mmol) was added and the mixture warmed to rt and stirred for 6 h. The reaction was quenched with sat. aq. $NaHCO_3$ (10 mL) and water (3 mL) and extracted with CH_2Cl_2 (3 × 20 mL). The organic layers were dried ($MgSO_4$), and the solvent removed *in vacuo*. The crude product was purified using flash chromatography (1:4 petroleum ether, ethyl acetate) to give the *title compound* **3** (0.228 g, 55% over two steps) as a colourless oil.

Method B: *via* Tsuji-Trost allylation.

Method adapted from one reported by Dittrich *et al.*[11]

To allylic acetate **15** (78 mg, 0.239 mmol) in THF (3 mL) was added morpholine (0.04 mL, 0.464 mmol) dropwise, followed by catalytic tetrakis(triphenylphospine)palladium(0) (14 mg, 5 mol-%) and the suspension heated to 70 °C for 2 d, then reduced to 50 °C for an additional 3 d. The solvent was removed *in vacuo*, extracted with CH_2Cl_2 (3 x 10 mL), washed with sat. aq. $NaHCO_3$ (10 mL) and dried ($MgSO_4$). The crude product was purified using flash chromatography (1:3 petroleum ether, ethyl acetate) to give the *title compound* **3** (70 mg, 83% over two steps) as a yellow oil.

R_f = 0.17 (3:1 ethyl acetate, petroleum ether);

δ_H (400 MHz; $CDCl_3$) 2.45 (4H, s, H-3''), 2.99 (2H, d, *J* = 6.8 Hz, H-1'), 3.32 (2H, d, *J* = 6.8 Hz, H-4'), 3.72 (4H, t, *J* = 4.5 Hz, H-2''), 3.86 (6H, s, 4-OCH_3, 3-OCH_3), 5.54–5.60 (1H, m, H-3'), 5.73–5.80 (1H, m, H-2'), 6.69–6.72 (2H, m, H-2, H-6), 6.80 (1H, d, *J* = 8.0 Hz, H-5);

δ_C (100 MHz; $CDCl_3$) 38.6 (C-4'), 53.7 (C-3''), 55.9 (3-OCH_3 or 4-OCH_3), 56.1 (3-OCH_3 or 4-OCH_3), 61.2 (C-1'), 67.1 (C-2''), 111.5 (C-5), 112.0 (C-2), 120.5 (C-6), 127.2 (C-3'), 133.0 (C-1), 133.9 (C-2'), 147.6 (C-4), 149.1 (C-3).

The obtained spectroscopic data was in agreement with literature values.[5]

(2*R,3*S**)-2-(4-(Benzyloxy)-3-methoxybenzyl)-3-(3,4-dimethoxybenzyl)-1-morpholinopent-4-en-1-one (1)**

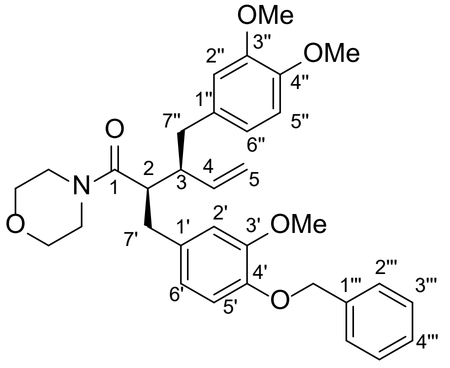

The reaction was carried out according to general procedure A using acid chloride **2** (0.684 g, 2.25 mmol) and allylic morpholine **3** (0.519 g, 1.87 mmol) for 23 h. The crude product was purified using flash chromatography (1:3 petroleum ether, ethyl acetate) to give the *title compound* **1** (0.868 g, 85%) as a yellow oil.

R_f = 0.42 (1:3 petroleum ether, ethyl acetate);

δ_H (400 MHz; $CDCl_3$) 2.57 (1H, dd, *J* = 13.4, 9.0 Hz, $\underline{H}_A$-7''), 2.62–2.74 (2H, m, H-3, $OC\underline{H}_ACH_2N$), 2.76–2.81 (1H, m, $OCH_2C\underline{H}_AN$), 2.83–2.92 (4H, m, H-2, H-7', $\underline{H}_B$-7''), 2.96–3.02 (1H, ddd, *J* = 13.4, 7.6, 3.0 Hz, $OCH_2C\underline{H}_BN$), 3.20–3.32 (3H, m, $OC\underline{H}_BCH_2N$, $OC\underline{H}_CC\underline{H}_CN$), 3.50–3.55 (1H, m, $OC\underline{H}_DCH_2N$), 3.61–3.66 (1H, m, $OCH_2C\underline{H}_DN$), 3.84 (6H, s, 4''-OCH_3, 3'-OCH_3), 3.85 (3H, s, 3''-OCH_3), 4.88 (1H, dd, *J* = 17.1, 1.9 Hz, $\underline{H}_A$-5), 4.97 (1H, dd, *J* = 10.4, 1.9 Hz, $\underline{H}_B$-5), 5.12 (2H, s, 1'''-$C\underline{H_2}$), 5.84 (1H, ddd, *J* = 17.1, 10.4, 9.0 Hz, H-4), 6.59 (1H, dd, *J* = 8.0, 1.9 Hz, H-6'), 6.65–6.68 (2H, m, H-2'', H-6''), 6.69 (1H, d, *J* = 2.0 Hz, H-2') 6.74 (1H, d, *J* = 8.0 Hz, H-5'), 6.76 (1H, d, *J* = 8.0 Hz, H-5''), 7.25–7.29 (1H, m, H-4'''), 7.32–7.36 (2H, m, H-3'''), 7.38–7.41 (2H, m, H-2''');

δ_C (100 MHz; $CDCl_3$) 37.3 (C-7'), 38.3 (C-7''), 41.9 ($OCH_2\underline{C}H_{CD}N$), 46.3 (C-2), 46.5 ($OCH_2\underline{C}H_{AB}N$), 48.5 (C-3), 55.96, 55.98, 56.2 (3''-OCH_3, 4''-OCH_3, 3'-OCH_3), 66.3 ($O\underline{C}H_{AB}CH_2N$), 66.9 ($O\underline{C}H_{CD}CH_2N$), 71.2 (1'''-$CH_2$), 111.1 (C-5''), 112.4 (C-2''), 113.3 (C-2'), 114.5 (C-5'), 116.7 (C-5), 120.9 (C-6'), 121.3 (C-6''), 127.3 (C-2'''), 127.9 (C-4'''), 128.6 (C-3'''), 132.3 (C-1''), 133.1 (C-1'), 137.3 (C-1'''), 139.3 (C-4), 146.7 (C-4'), 147.4 (C-4''), 148.8 (C-3''), 149.7 (C-3'), 172.7 (C=O);

IR ν_{max}(ATR)/cm^{-1}: 2933, 2855, 1630, 1513, 1453, 1232, 1139, 1027, 915, 765;

***m/z* (ESI$^+$)**: 568 (MNa^+, 100%); **HRMS** (ESI$^+$) Found (MNa^+): 568.2682; $C_{33}H_{39}NNaO_6$ requires 568.2670.

(8*R*[*],8'*R*[*],9'*R*[*])-8-(4-(Benzyloxy)-3-methoxybenzyl)-8'-(3',4'-dimethoxybenzyl)-9'-(hydroxymethyl)dihydrofuran-9(8*H*)-one (16) and

(2*R*[*],3*R*[*],4*S*[*])-2-(4'-(benzyloxy)-3'-methoxybenzyl)-3-(3'',4''-dimethoxybenzyl)-4,5-dihydroxy-1-morpholinopentan-1-one (*syn*-17)

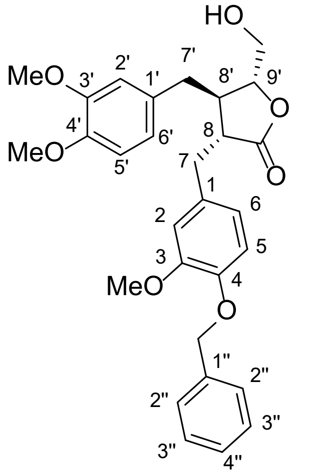

16

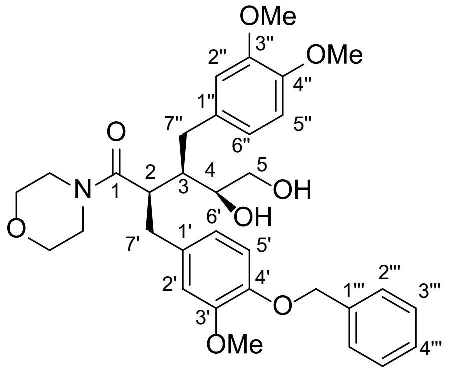

syn-**17**

The reaction was carried out according to general procedure B using amide **1** (167 mg, 0.307 mmol) in *t*-BuOH/H_2O (8 mL) for 4 d. The crude product was purified using flash chromatography (1:4 petroleum ether, ethyl acetate) to give the *title compound* **16** (0.133 g, 88%) as a white solid. **m.p.** 25–27 °C.

R_f = 0.50 (1:4 petroleum ether, ethyl acetate);

δ_H (400 MHz; $CDCl_3$) 1.57 (1H, t, *J* = 6.5 Hz, OH), 2.34–2.40 (1H, m, H-8'), 2.50 (1H, dd, *J* = 13.5, 8.0 Hz, H_A-7'), 2.59 (1H, dd, *J* = 13.5, 6.0 Hz, H_B-7'), 2.69 (1H, dt, *J* = 9.6, 6.0 Hz, H-8), 2.90 (1H, dd, *J* = 14.1, 6.2 Hz, H_A-7), 2.94 (1H, dd, *J* = 14.1, 5.6 Hz, H_B-7), 3.10 (1H, dd, *J* = 12.6, 5.2 Hz, 9'-CH_A), 3.48 (1H, dd, *J* = 12.6, 2.5 Hz, 9'-CH_B), 3.80 (3H, s, 3-OCH_3), 3.85 (6H, s, 3'-OCH_3, 4'-OCH_3), 4.18 (1H, ddd, 8.3, 5.2, 2.5 Hz, H-9'), 5.12 (2H, s, 1''-CH_2), 6.46 (1H, d, *J* = 2.0 Hz, H-2'), 6.56 (1H, dd, *J* = 8.0, 2.0 Hz, H-6'), 6.61 (1H, dd, *J* = 8.0, 2.0 Hz, H-6), 6.72 (1H, d, *J* = 2.0 Hz, H-2), 6.75 (1H, d, *J* = 8.0 Hz, H-5'), 6.79 (1H, d, *J* = 8.0 Hz, H-5), 7.26–7.30 (1H, m, H-4''), 7.31–7.36 (2H, m, H-3''), 7.39–7.42 (2H, m, H-2'');

δ_C (100 MHz; $CDCl_3$) 35.1 (C-7), 38.6 (C-7'), 41.7 (C-8'), 47.6 (C-8), 56.0 (3'-OCH_3 and 4'-OCH_3), 56.2 (3-OCH_3), 63.3 (9'-CH_2), 71.3 (1''-CH_2), 84.0 (C-9'), 111.5 (C-5'), 112.1 (C-2'), 113.3 (C-2), 114.3 (C-5), 121.0 (C-6'), 121.6 (C-6), 127.4 (C-2''), 128.0 (C-4''), 128.7 (C-3''), 130.3 (C-1'), 131.1 (C-1), 137.2 (C-1''), 147.2 (C-4), 148.2 (C-4'), 149.3 (C-3'), 150.0 (C-3), 177.7 (C-9);

IR ν_{max}(ATR)/cm^{-1}: 3523, 2928, 2809, 1759, 1513, 1138, 1023;

***m/z* (ESI$^+$)**: 515 (MNa^+, 100%), 510 (44%), 493 (MH^+, 33%), 227 (24%); **HRMS** (ESI$^+$) Found (MNa^+): 515.2033; $C_{29}H_{32}NaO_7$ requires 512.2040.

In the methanol flush of the flash chromatography column, the *title compound syn*-**17** (25 mg, 14%) was also obtained as a yellow oil.

R_f = 0.06 (1:4 petroleum ether, ethyl acetate);

δ_H (400 MHz; $CDCl_3$) 2.12 (1H, dt, *J* = 11.0, 4.7 Hz, H-2), 2.39–2.51 (3H, m, $OC\underline{H_A}CH_2N$, 7''-H_A, $OCH_2NC\underline{H_A}$), 2.58 (1H, ddd, *J* = 12.8, 7.8, 3.4 Hz, $OCH_2NC\underline{H_B}$), 2.73 (1H, dd, *J* = 12.8, 5.2 Hz, 7'-H_A), 2.88 (1H, dd, *J* = 15.1, 4.5 Hz, 7''-H_B), 2.94 (1H, dd, *J* = 12.8, 10.2 Hz, 7'-H_B), 3.03 (1H, dt, *J* = 10.5, 5.2 Hz, H-3), 3.09 (1H, ddd, *J* = 11.1, 5.2, 3.5, $OC\underline{H_B}NCH_2$), 3.25–3.35 (2H, m, $OCH_2NC\underline{H_C}$, OCH_C), 3.51 (1H, dd, *J* = 10.8, 4.6 Hz, 5-H_A), 3.56–3.59 (1H, m, $OC\underline{H_D}NCH_2$), 3.70–3.75 (1H, m, $OCH_2NC\underline{H_D}$), 3.74 (1H, dd, *J* = 10.6, 8.1 Hz, 5-CH_B), 3.82 (3H, s, 4'' or 3'-OCH_3), 3.83 (3H, s, 4''- or 3'-OCH_3), 3.84 (3H, s, 3''-OCH_3), 4.33 (1H, dd, *J* = 8.1, 4.6 Hz, H-4), 5.11 (2H, s, 1'''-CH_2), 6.49 (1H, dd, *J* = 8.1, 2.0 Hz, H-6'), 6.57–6.61 (3H, d, *J* = 2.0 Hz, H-2', H-2'', H-6''), 6.71 (1H, d, *J* = 8.1 Hz, H-5'), 6.75 (1H, d, *J* = 8.0 Hz, H-5''), 7.25–7.29 (1H, m, H-4'''), 7.32–7.36 (2H, m, H-3'''), 7.37–7.40 (2H, m, H-2''');

δ_C (100 MHz; $CDCl_3$) 32.4 (C-7''), 35.1 (C-7'), 38.5 ($OCH_2N\underline{C_{AB}}$), 42.4 ($OCH_2N\underline{C_{CD}}$), 43.7 (C-2), 44.0 (C-3), 56.0 (3'OCH_3 or 3''-OCH_3 or 4''-OCH_3), 56.1 (3'OCH_3 or 3''-OCH_3 or 4''-OCH_3), 56.2 (3'OCH_3 or 3''-OCH_3 or 4''-OCH_3), 65.9 ($O\underline{C_{AB}}NCH_2$), 66.6 (C-5) 66.7 ($O\underline{C_{CD}}NCH_2$), 69.2 (C-4), 71.3 (C-1'''), 111.5 (C-5''), 112.1 (C-2''), 113.1 (C-2'), 114.1 (C-5'), 121.0 (C-6 and C-6''), 127.5 (C-2'''), 128.0 (C-4'''), 128.7 (C-3''') 131.9 (C-1''), 132.5 (C-1') 137.2 (C-1''') 147.2 (C-4'), 147.7 (C-4''), 149.33 (C-3'), 150.1 (C-3''), 174.4 (C-1);

IR ν_{max}(ATR)/cm^{-1}: 3384, 2927, 2856, 1762, 1513, 1235, 1025;

***m/z* (ESI$^+$)**: 602 (MNa^+, 100%); **HRMS** (ESI$^+$) Found (MNa^+): 602.2723; $C_{33}H_{41}NNaO_8$ requires 602.2724.

((8R^*,8'R^*,9'R^*)-8-(4-(Benzyloxy)-3-methoxybenzyl)-3'-(3',4'-dimethoxybenzyl)-9'-oxotetrahydrofuran-9-yl)methyl acetate (21)

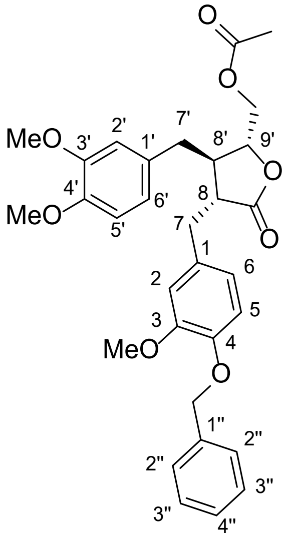

The reaction was carried out according to general procedure C using alcohol **16** (49 mg, 0.0995 mmol) and acetyl chloride (0.01 mL, 0.115 mmol) for 23 h. The crude product was purified using flash chromatography (9:1 petroleum ether, ethyl acetate) to give the *title compound* **21** (52 mg, 88%) as a yellow oil.

R_f = 0.32 (2:1 petroleum ether, ethyl acetate);

δ_H (400 MHz; $CDCl_3$) 1.93 (3H, s, OCOC$\underline{H_3}$), 2.18–2.26 (1H, m, H-8'), 2.62 (2H, d, *J* = 6.8 Hz, H-7'), 2.71 (1H, dt, *J* = 10.0, 5.5 Hz, H-8), 2.80 (1H, dd, *J* = 14.0, 5.5 Hz, H-7), 2.99 (1H, dd, *J* = 14.0, 5.9 Hz, H-7), 3.72 (1H, dd, *J* = 12.3, 5.9 Hz, 9'-CH_A), 3.80 (3H, s, 3-OCH_3), 3.84 (3H, s, 3'-OCH_3), 3.86 (3H, s, 4'-OCH_3), 4.00 (1H, dd, *J* = 12.3, 2.5, 9'-CH_B), 4.28 (1H, ddd, *J* = 8.5, 5.9, 2.5 Hz, H-9'), 5.12 (2H, s, 1''-CH_2), 6.50 (1H, d, *J* = 2.0 Hz, H-2'), 6.51 (1H, dd, *J* = 8.1, 2.0 Hz, H-6), 6.57 (1H, dd, *J* = 8.1, 2.0 Hz, H-6'), 6.67 (1H, d, *J* = 2.0 Hz, H-2), 6.766 (1H, d, *J* = 8.1 Hz, H-5'), 6.775 (1H, d, *J* = 8.1 Hz, H-5), 7.26–7.30 (1H, m, H-4''), 7.32–7.36 (2H, m, H-3''), 7.40–7.42 (2H, m, H-2'');

δ_C (100 MHz; $CDCl_3$) 20.7 (OCO$\underline{C}H_3$), 34.5 (C-7), 38.1 (C-7'), 42.1 (C-8'), 47.1 (C-8), 56.0 (3-OCH_3 or 3'-OCH_3 or 4'-OCH_3), 56.1 (3-OCH_3 or 3'-OCH_3 or 4'-OCH_3), 56.2 (3-OCH_3 or 3'-OCH_3 or 4'-OCH_3), 64.1 (9'-CH_2), 71.2 (1''-CH_2), 80.5 (C-9'), 111.6 (C-5'), 112.3 (C-2'), 113.3 (C-2), 114.2 (C-5), 121.1 (C-6'), 121.7 (C-6), 127.4 (C-2''), 128.0 (C-4''), 128.7 (C-3''), 129.9 (C-1'), 130.7 (C-1), 137.2 (C-1''), 147.3 (C-4), 148.3 (C-4'), 149.3 (C-3), 150.0 (C-3'), 170.6 (O$\underline{C}$OCH_3), 177.3 (C-9);

IR ν_{max}(ATR)/cm^{-1}: 2927, 2856, 1768, 1740, 1513, 1174, 1025;

***m/z* (ESI$^+$)**: 557 (MNa^+, 100%); **HRMS** (ESI$^+$) Found (MNa^+): 557.2154; $C_{31}H_{34}NaO_8$ requires 557.2146.

((8R^*,8'R^*,9'R^*)-8-(4-(Benzyloxy)-3-methoxybenzyl)-8'-(3',4'-dimethoxybenzyl)-9'-oxotetrahydrofuran-9-yl)methyl propionate (22)

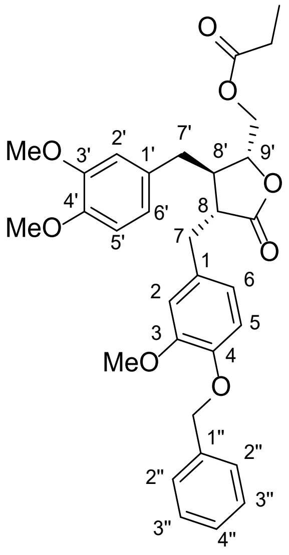

The reaction was carried out according to general procedure C using alcohol **16** (62 mg, 0.126 mmol) and propionyl chloride (0.01 mL, 0.115 mmol) for 4 h. The crude product was purified using flash chromatography (4:1 petroleum ether, ethyl acetate) to give the *title compound* **22** (50 mg, 72%) as a yellow oil.

R_f = 0.48 (4:1 petroleum ether, ethyl acetate);

δ_H (400 MHz; $CDCl_3$) 1.07 (3H, t, *J* = 7.5 Hz, -$OCOCH_2C\underline{H_3}$), 2.16–2.27 (3H, m, H-8', -$OCOC\underline{H_2}CH_3$), 2.62 (2H, d, *J* = 6.8 Hz, H-7'), 2.70 (1H, dt, *J* = 10.0, 5.5 Hz, H-8), 2.81 (1H, dd, *J* = 14.0, 5.5 Hz, H-7), 2.98 (1H, dd, *J* = 14.0, 5.9 Hz, H-7), 3.73 (1H, dd, *J* = 12.5, 5.9 Hz, 9'-CH_A), 3.81 (3H, s, 3-OCH_3), 3.84 (3H, s, 3'-OCH_3), 3.86 (3H, s, 4'-OCH_3), 4.04 (1H, dd, *J* =12.5, 2.5 Hz, 9'-CH_B), 4.29 (1H, ddd, *J* = 8.5, 5.9, 2.5 Hz, H-9'), 5.11 (2H, s, 1''-CH_2), 6.51 (2H, d, *J* = 2.5 Hz, H-2'), 6.51 (1H, dd, *J* = 8.0, 2.0 Hz, H-6), 6.57 (1H, dd, *J* = 8.0, 2.0 Hz, H-6'), 6.67 (1H, d, *J* = 2.0 Hz, H-2), 6.77 (1H, d, *J* = 8.0 Hz, H-5'), 6.77 (1H, d, *J* = 8.0 Hz, H-5), 7.26–7.30 (1H, m, H-4''), 7.32–7.36 (2H, m, H-3''), 7.40–7.43 (2H, m, H-2'');

δ_C (100 MHz; $CDCl_3$) 9.1 ($OCOCH_2\underline{C}H_3$), 27.3 ($OCO\underline{C}H_2CH_3$), 34.5 (C-7), 38.1 (C-7'), 42.1 (C-8'), 47.1 (C-8), 56.0 (3'-OCH_3 or 4'-OCH_3), 56.05 (3'-OCH_3 or 4'-OCH_3), 56.14 (3-OCH_3), 64.0 (9'-CH_2), 71.2 (1''-CH_2), 80.6 (C-9'), 111.5 (C-5'), 112.4 (C-2'), 113.3 (C-2), 114.1 (C-5), 121.1 (C-6'), 121.7 (C-6), 127.4 (C-2''), 128.0 (C-4''), 128.7 (C-3''), 129.9 (C-1'), 130.7 (C-1), 137.2 (C-1''), 147.3 (C-4), 148.2 (C-4'), 149.3 (C-3), 150.0 (C-3'), 174.0 ($O\underline{C}OCH_2CH_3$), 177.3 (C-9);

IR ν_{max}(ATR)/cm^{-1}: 2938, 2843, 1768, 1738, 1514, 1142, 1027;

***m/z* (ESI^+)**: 571 (MNa^+, 100%); **HRMS** (ESI^+) Found (MNa^+): 571.2309; $C_{32}H_{36}NaO_8$ requires 571.2302.

((8R^*,8'R^*,9'R^*)-8-(4-(Benzyloxy)-3-methoxybenzyl)-8'-(3',4'-dimethoxybenzyl)-9'-oxotetrahydrofuran-9-yl)methyl benzoate (23)

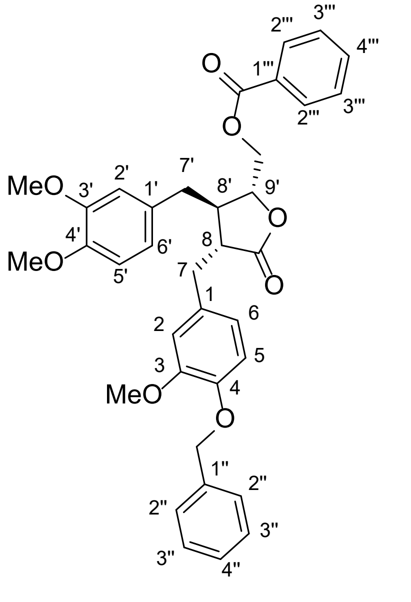

The reaction was carried out according to general procedure C using alcohol **16** (84 mg, 0.171 mmol) and benzoyl chloride (0.02 mL, 0.205 mmol) for 20 h. The crude product was purified using flash chromatography (3:1 petroleum ether, ethyl acetate) to give the *title compound* **23** (83 mg, 81%) as a colourless oil.

R_f = 0.31 (3:1 petroleum ether, ethyl acetate);

δ_H (400 MHz; $CDCl_3$) 2.36–2.44 (1H, m, H-8'), 2.64 (1H, dd, *J* = 14.0, 7.5 Hz, H-7'), 2.72 (1H, dd, *J* = 14.0, 6.0 Hz, H-7'), 2.75 (1H, dt, *J* = 11.0, 5.6 Hz, H-8), 2.86 (1H, dd, *J* = 14.1, 5.6 Hz, H-7), 3.04 (1H, dd, *J* = 14.0, 5.6 Hz, H-7), 3.72 (3H, s, 3-OCH_3), 3.82 (3H, s, 3'-OCH_3), 3.84 (3H, s, 4'-OCH_3), 3.94 (1H, dd, *J* = 12.5, 5.5 Hz, 9'-CH_A), 4.29 (1H, dd, *J* = 12.5, 2.6 Hz, 9'-CH_B), 4.42 (1H, ddd, *J* = 8.4, 5.5, 2.6 Hz, H-9'), 5.07 (2H, s, 1''-CH_2), 6.51 (2H, d, *J* = 2.0 Hz, H-2'), 6.53 (1H, dd, *J* = 8.0, 2.0 Hz, H-6), 6.60 (1H, dd, *J* = 8.0, 2.0 Hz, H-6'), 6.71 (1H, d, *J* = 2.0 Hz, H-2), 6.75 (2H, d, *J* = 8.0 Hz, H-5, H-5'), 7.26–7.30 (1H, m, H-4''), 7.32–7.36 (2H, m, H-3''), 7.39–7.46 (4H, m, H-2'', H-3'''), 7.52–7.56 (1H, m, H-4'''), 7.85–7.88 (2H, m, H-2''');

δ_C (100 MHz; $CDCl_3$) 34.2 (C-7), 37.8 (C-7'), 42.0 (C-8'), 47.2 (C-8), 55.9 (C-3), 56.0 (3'-OCH_3 or 4'-OCH_3), 56.1 (3'-OCH_3 or 4'-OCH_3), 64.2 (9'-CH_2), 71.1 (1''-CH_2), 80.6 (C-9'), 111.5 (C-5'), 112.2 (C-2'), 113.2 (C-5), 114.1 (C-2), 121.1 (C-6'), 121.7 (C-6), 127.4 (C-2''), 128.0 (C-4''), 128.6 (C-3''), 129.4 (C-1'''), 129.7 (C-2'''), 129.8 (C-1), 130.5 (C-1'), 133.5 (C-4'''), 137.2 (C-1''), 147.3 (C-4), 148.3 (C-4'), 149.3 (C-3), 150.0 (C-3'), 166.1 (1'''-COO), 177.3 (C-9);

IR ν_{max}(ATR)/cm^{-1}: 2936, 2837, 1770, 1720, 1513, 1174, 1026;

***m/z* (ESI$^+$)**: 619 (MNa^+, 100); **HRMS** (ESI$^+$) Found (MNa^+): 619.2282; $C_{36}H_{36}NaO_8$ requires 619.2282.

((8*R**,8'*R**,9'*R**)-8-(4-(Benzyloxy)-3-methoxybenzyl)-8'-(3',4'-dimethoxybenzyl)-9'-oxotetrahydrofuran-9-yl)methyl methanesulfonate (31)

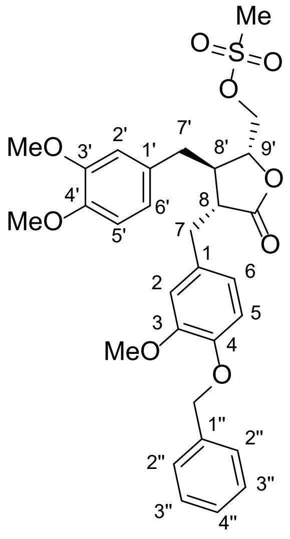

To a stirred solution of alcohol **16** (77 mg, 0.156 mmol) in CH_2Cl_2 (3 mL), under nitrogen at 0 °C, was added triethylamine (0.03 mL, 0.215 mmol), followed by MsCl (0.02 mL, 0.238 mmol), after 5 min. The mixture was stirred for 2.5 h then quenched with the addition of $NaHCO_3$ (3 mL), extracted with CH_2Cl_2 (3 × 10 mL) and dried ($MgSO_4$). The solvent was removed *in vacuo* to give the *title compound* **31** as a yellow oil which was used immediately in the next step.

(8R^*,8'R^*,9'R^*)-9'-(Azidomethyl)-8-(4-(benzyloxy)-3-methoxybenzyl)-8'-(3',4'-dimethoxybenzyl)dihydrofuran-9(8*H*)-one (29) and (8R^*,8'R^*,9R^*)-8-(4-(Benzyloxy)-3-methoxybenzyl)-9'-(chloromethyl)-8'-(3',4'-dimethoxybenzyl)dihydrofuran-9(8*H*)-one (30)

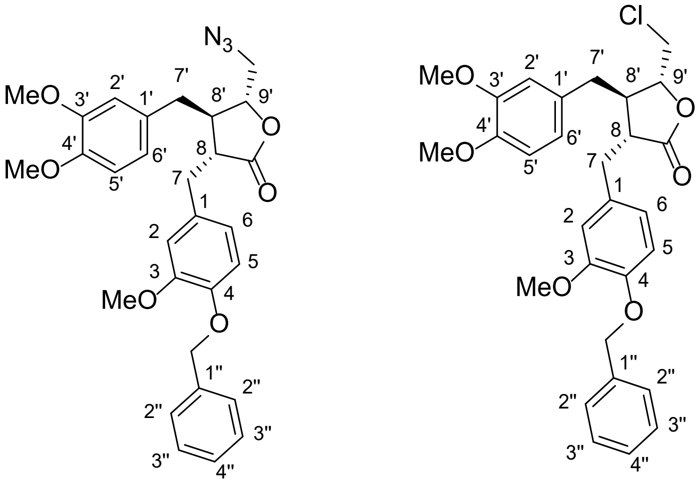

29 **30**

To a stirred solution of mesylate **31** (89 mg, 0.156 mmol) in DMF (3 mL) was added NaN_3 (38 mg, 0.585 mmol) and the mixture heated for 85 °C for 20 h, followed by 100 °C for 7 h. The mixture was cooled to rt, quenched with water (3 mL), extracted with EtOAc (3 × 10 mL), washed with water (10 mL) and brine (10 mL), then dried (Na_2SO_4). The solvent was removed *in vacuo* and the crude product purified using flash chromatography (2:1 petroleum ether: ethyl acetate) to give the *title compound* **29** (61 mg, 78%) as a yellow oil.

R_f = 0.85 (1:2 petroleum ether, ethyl acetate);

δ_H (400 MHz; $CDCl_3$) 2.29–2.37 (1H, m, H-8'), 2.45 (1H, dd, *J* = 14.0, 8.5 Hz, H-7'), 2.62 (1H, dd, *J* = 14.0, 5.8 Hz, H-7'), 2.68 (1H, dd, *J* = 13.5, 5.0 Hz, 9'-CH_A), 2.69 (1H, dt, *J* = 11.8, 5.6 Hz, H-8), 2.91 (1H, dd, *J* = 14.0, 5.6 Hz, H-7), 2.99 (1H, dd, *J* = 14.0, 6.4 Hz, H-7), 3.10 (1H, dd, *J* = 14.0, 3.1 Hz, 9'-CH_B), 3.80 (3H, s, 3-OCH_3), 3.85 (3H, s, 3'-OCH_3), 3.86 (3H, s, 4'-OCH_3), 4.19 (1H, ddd, *J* = 8.0, 5.0, 3.1 Hz, H-9'), 5.12 (1''-CH_2), 6.46 (1H, d, *J* = 2.0 Hz, H-2'), 6.55 (1H, dd, *J* = 8.0, 2.0 Hz, H-6'), 6.62 (1H, dd, *J* = 8.0, 2.0 Hz, H-6), 6.74 (1H, d, *J* = 2.0 Hz, H-2), 6.76 (1H, d, *J* = 8.0 Hz, H-5'), 6.81 (1H, d, *J* = 8.0 Hz, H-5), 7.26–7.29 (1H, m, H-4''), 7.33 (2H, t, *J* = 7.70 Hz, H-3''), 7.41 (2H, d, *J* = 7.70 Hz, H-2'');

δ_C (100 MHz; $CDCl_3$) 34.9 (C-7), 38.6 (C-7'), 42.8 (C-8'), 47.5 (C-8), 53.1 (9'-CH_2), 56.01 (3'-OCH_3 or 4'-OCH_3), 56.04 (3'-OCH_3 or 4'-OCH_3), 56.2 (3-OCH_3), 71.3 (1''-CH_2), 81.8 (C-9'), 111.5 (C-5'), 111.9 (C-2'), 113.2 (C-2), 114.4 (C-5), 120.8 (C-6'), 121.6 (C-6), 127.4 (C-2''), 128.0 (C-4''), 128.6 (C-3''), 130.1 (C-1'), 130.8 (C-1), 137.2 (C-1''), 147.3 (C-4), 148.3 (C-4'), 149.4 (C-3'), 150.1 (C-3), 177.0 (C-9);

IR ν_{max}(ATR)/cm^{-1}: 2929, 2848, 2102, 1771, 1513, 1236, 1156, 1027;

***m/z* (ESI$^+$)**: 540 (MNa$^+$, 100%); **HRMS** (ESI$^+$) Found (MNa$^+$): 540.2109; $C_{29}H_{31}N_3NaO_6$ requires 540.2105.

In a separate fraction, the *title compound* **30** (20 mg, 25%) was also obtained as a yellow oil.

R_f = 0.39 (3:1 petroleum ether, ethyl acetate);

δ_H (400 MHz; $CDCl_3$) 2.46–2.52 (2H, m, H-8', H-7'), 2.57–2.63 (1H, m, H-7'), 2.68–2.73 (1H, m, H-8), 2.91 (1H, dd, *J* = 14.1, 5.3 Hz, H-7), 2.94 (1H, dd, *J* = 12.0, 4.3 Hz, 9'-CH_B), 2.97 (1H, dd, *J* = 14.1, 6.5 Hz, H-7), 3.29 (1H, dd, *J* = 12.0, 3.8 Hz, 9'-CH_A), 3.80 (3H, s, 3-OCH_3), 3.860 (3H, s, 3'-OCH_3 or 4'-OCH_3), 3.863 (3H, s, 3'-OCH_3 or 4'-OCH_3), 4.30 (1H, dt, *J* = 7.1, 3.8 Hz, H-9'), 5.13 (2H, s, 1''-CH_2), 6.47 (1H, d, *J* = 2.0 Hz, H-2'), 6.56 (1H, dd, *J* = 8.0, 2.0 Hz, H-6'), 6.62 (1H, dd, *J* = 8.0, 2.0 Hz, H-6), 6.73 (1H, d, *J* = 2.0 Hz, H-2), 6.76 (1H, d, *J* = 8.0 Hz, H-5'), 6.80 (1H, d, *J* = 8.0 Hz, H-5), 7.25–7.29 (1H, m, H-4''), 7.31–7.35 (2H, m, H-3''), 7.39–7.42 (1H, m, H-2'');

δ_C (100 MHz; $CDCl_3$) 35.0 (C-7), 38.9 (C-7'), 43.1 (C-8'), 45.3 (9'-CH_2), 47.6 (C-8), 56.01 (3-OCH_3 or 3'-OCH_3 or 4'-OCH_3), 56.04 (3-OCH_3 or 3'-OCH_3 or 4'-OCH_3), 56.2 (3-OCH_3 or 3'-OCH_3 or 4'-OCH_3), 71.3 (1''-CH_2), 81.8 (C-9'), 111.5 (C-5'), 111.9 (C-2'), 113.2 (C-2), 114.5 (C-5), 120.8 (C-6'), 121.6 (C-6), 127.4 (C-2''), 128.0 (C-4''), 128.7 (C-3''), 130.1 (C-1'), 130.9 (C-1), 137.2 (C-1''), 147.3 (C-4), 148.3 (C-4'), 149.3 (C-3), 150.1 (C-3'), 177.0 (C-9);

IR ν_{max}(ATR)/cm^{-1}: 2920, 2851, 1770, 1513, 1139, 1013, 738;

***m/z* (ESI$^+$)**: 535 (^{37}ClMNa$^+$, 33%), 533 (^{35}ClMNa$^+$, 100%); **HRMS** (ESI$^+$) Found (^{37}ClMNa$^+$): 535.1687; $C_{29}H_{28}{}^{37}ClNaO_6$ requires 535.1684. **HRMS** (ESI$^+$) Found (^{35}ClMNa$^+$): 533.1701; $C_{29}H_{28}{}^{35}ClNaO_6$ requires 533.1700.

(8*R,8'*R**,9'*R**)-8-(4-(Benzyloxy)-3-methoxybenzyl)-8'-(3',4'-dimethoxybenzyl)-9'-((4''-phenyl-1H-1'',2'',3''-triazol-1''-yl)methyl)dihydrofuran-9(8*H*)-one (32)**

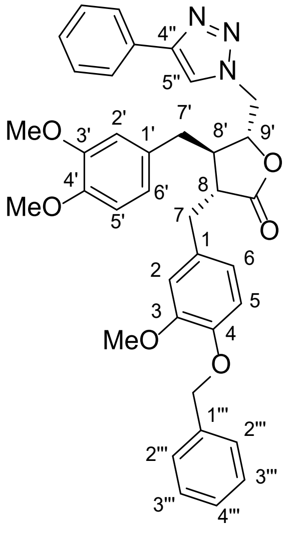

To a solution of azide **29** (32 mg, 0.0618 mmol) in MeCN (3 mL) was added phenylacetylene (0.01 mL, 0.0911 mmol). Sodium ascorbate (1M solution in water, 0.01 mL) was added, followed by $Cu_2SO_4{\cdot}5H_2O$ (0.3 M solution in water, 0.04 mL). The mixture was vigorously stirred for 49 h then diluted with water (5 mL) and extracted with EtOAc (3 × 10 mL), washed with brine, and dried (Na_2SO_4). The solvent was removed *in vacuo* and the crude product purified using flash chromatography (4:1 petroleum ether: ethyl acetate) to give the *title compound* **32** (22 mg, 58%) as a white solid. **m.p.** 59–61 °C.

R_f = 0.32 (2:1 petroleum ether, ethyl acetate);

δ_H (400 MHz; $CDCl_3$) 2.22–2.30 (1H, m, H-8'), 2.52 (1H, dd, *J* =13.8, 8.1 Hz, H_A-7'), 2.59 (1H, dd, 13.8, 6.7 Hz, 7'-H_B), 2.61 (1H, dd, 13.8, 5.0 Hz, 7-H_A), 2.66–2.71 (1H, ddd, *J* = 9.4, 6.7, 5.0 Hz, H-8), 2.84 (1H, dd, *J* = 13.8, 5.0 Hz, 7-H_B), 3.85 (3H, s, 4'-OCH_3), 3.86 (3H, s, 3'-OCH_3), 3.87 (3H, s, 3-OCH_3), 3.95 (1H, dd, *J* = 14.8, 5.6 Hz, 9'-CH_A), 4.26 (1H, dd, *J* = 14.8, 3.1 Hz, 9'-CH_B), 4.46 (1H, ddd, *J* = 8.0, 5.6, 3.1 Hz, H-9'), 5.05 (2H, s, 1''-CH_2), 6.54 (1H, dd, *J* = 8.1, 2.0 Hz, H-6), 6.56 (1H, d, *J* = 2.0 Hz, H-2'), 6.62 (1H, dd, *J* = 8.1, 2.0 Hz, H-6'), 6.64 (1H, d, *J* = 2.0 Hz, H-2), 6.75 (1H, d, *J* = 8.1 Hz, H-5'), 6.79 (1H, d, *J* = 8.1 Hz, H-5), 7.23–7.27 (1H, m, 4''-H*para*) 7.29–7.35 (3H, m, H-4''', H-3''') 7.36–7.41 (4H, m, H-2''', 4''-H*meta*), 7.71 (1H, s, H-5''), 7.78–7.80 (2H, m, 4''-H*ortho*);

δ_C (100 MHz; $CDCl_3$) 34.9 (C-7), 38.7 (C-7'), 42.6 (C-8'), 47.3 (C-8), 52.3 (9'-CH_2), 56.0 (3-OCH_3 or 3'-OCH_3 or 4'-OCH_3), 56.1 (3-OCH_3 or 3'-OCH_3 or 4'-OCH_3), 56.2 (3-OCH_3 or 3'-OCH_3 or 4'-OCH_3), 71.1 (1''-CH_2), 80.9 (C-9'), 111.6 (C-5), 112.2 (C-2'), 113.3 (C-2), 114.4 (C-5'), 121.1 (C-6', 4''-C), 121.4 (C-6), 125.9 (4''-C*ortho*), 127.4 (C-2'''), 128.0 (C-4'''), 128.5 (4''-C*para*), 128.6 (4''-C*meta*), 129.0 (C-3'''), 129.5 (C-1),

130.0 (C-5''), 130.2 (C-1'), 137.2 (C-1'''), 147.3 (C-4, C-4'), 148.3 (C-4''), 149.5 (C-3'), 150.0 (C-3), 176.7 (C-9);

IR ν_{max}(ATR)/cm^{-1}: 2927, 2856, 1773, 1513, 1236, 1158, 1025;

***m/z* (ESI$^+$)**: 642 (MNa^+, 100%), 620 (MH^+, 51%); **HRMS** (ESI$^+$) Found (MNa^+): 642.2575; $C_{37}H_{37}N_3NaO_6$ requires 642.2578.

(8*R**,8'*R**,9'*R**)-8'-(3',4'-Dimethoxybenzyl)-8-(4-hydroxy-3-methoxybenzyl)-9'-(hydroxymethyl)dihydrofuran-9(8*H*)-one (20)

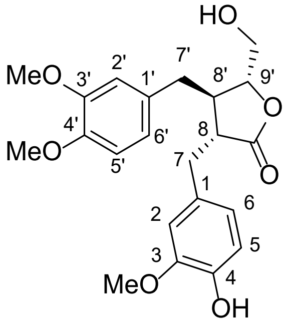

The reaction was carried out according to general procedure D using alcohol **16** (27 mg, 0.0548 mmol) for 19 h to give the *title compound* **20** (22 mg, quant.) as an off-white solid. **m.p.** 179–181 °C.

δ_H (400 MHz; $(CD_3)_2SO$) 2.19–2.27 (1H, m, H-8'), 2.44 (1H, dd, *J* = 13.8, 6.0 Hz, H-7'), 2.50 (1H, dd, *J* = 13.2, 8.0 Hz, H-7'), 2.64–2.79 (3H, m, H-8, H-7), 2.95 (1H, dt, *J* = 12.0, 5.5 Hz, 9'-CH_A), 3.23 (1H, ddd, *J* = 12.0, 5.5, 2.5 Hz, 9'-CH_B), 3.70 (3H, s, 3-OCH_3), 3.71 (3H, s, 3'-OCH_3 or 4'-OCH_3), 4.13 (1H, ddd, *J* = 8.5, 5.5, 2.5 Hz, H-9'), 4.87 (t, *J* = 5.8 Hz, 9'-$CH_2O\underline{H}$), 6.54 (1H, dd, *J* = 8.0, 2.0 Hz, H-6'), 6.60 (1H, dd, *J* = 8.0, 2.0 Hz, H-6), 6.62 (1H, d, *J* = 2.0 Hz, H-2'), 6.67 (1H, d, *J* = 8.0 Hz, H-5'), 6.69 (1H, d, *J* = 2.0 Hz, H-2), 6.83 (1H, d, *J* = 8.0 Hz, H-5), 8.75 (1H, s, 4-OH);

δ_C (100 MHz; $(CD_3)_2SO$) 34.3 (C-7), 37.3 (C-7'), 41.4 (C-8'), 46.6 (C-8), 55.4 (3-OCH_3, 4'-OCH_3), 55.5 (3'-OCH_3), 61.9 (9'-CH_2), 83.8 (C-9'), 111.7 (C-5'), 112.7 (C-2), 113.4 (C-2'), 115.3 (C-5) 120.8 (C-6), 121.5 (C-6'), 129.0 (C-1), 131.2 (C-1'), 145.0 (C-4'), 147.3 (C-3'), 147.4 (C-4), 148.6 (C-3), 177.7 (C-9);

δ_H (400 MHz; $CDCl_3$) 1.62 (1H, t, *J* = 7.0 Hz, 9'-$CH_2O\underline{H}$), 2.35–2.43 (1H, m, H-8'), 2.52 (1H, dd, *J* = 13.6, 8.0 Hz, H-7'), 2.62 (1H, dd, *J* = 13.6, 6.1 Hz, H-7'), 2.69 (1H, dt, *J* = 9.5, 6.1 Hz, H-8), 2.92 (2H, d, *J* = 6.1 Hz, H-7), 3.13 (1H, dt, *J* = 12.6, 5.5 Hz, 9'-CH_A), 3.49–3.53 (1H, m, 9'-CH_B), 3.82 (3H, s, 3-OCH_3), 3.84 (3H, s, 3'-OCH_3 or 4'-OCH_3), 3.85 (3H, s, 3'-OCH_3 or 4'-OCH_3), 4.19 (1H, ddd, *J* = 7.9, 5.5, 2.5 Hz, H-9'), 5.52 (1H, s, 4-OH), 6.46 (1H, d, *J* = 2.0 Hz, H-2'), 6.57 (1H, dd, *J* = 8.0, 2.0 Hz, H-6'), 6.63 (1H, dd, *J* = 8.0, 2.0 Hz, H-6), 6.66 (1H, d, *J* = 2.0 Hz, H-2), 6.76 (1H, d, *J* = 8.0 Hz, H-5'), 6.83 (1H, d, *J* = 8.0 Hz, H-5);

δ_C (100 MHz; $CDCl_3$) 35.1 (C-7), 38.6 (C-7'), 41.6 (C-8'), 47.7 (C-8), 56.02 (3-OCH_3, 4'-OCH_3), 56.06 (3'-OCH_3), 63.3 (9'-CH_2), 84.0 (C-9'), 111.5 (C-5'), 111.9 (C-2), 112.1 (C-2'), 114.3 (C-5), 121.0 (C-6), 122.3 (C-6'), 129.7 (C-1), 130.3 (C-1'), 144.7 (C-4), 146.8 (C-3'), 148.2 (C-4'), 149.3 (C-3), 177.8 (C-9);

IR ν_{max}(ATR)/cm^{-1}: 3516, 3391, 2924, 2850, 1745, 1515, 1256, 1154, 1022;

***m/z* (ESI$^+$)**: 425 (MNa^+, 100%); **HRMS** (ESI$^+$) Found (MNa^+): 425.1566; $C_{22}H_{26}NaO_7$ requires 425.1571.

((8*R,8'*R**,9'*R**)-8'-(3,4-Dimethoxybenzyl)-8-(4-hydroxy-3-methoxybenzyl)-9'-oxotetrahydrofuran-9-yl)methyl acetate (25)**

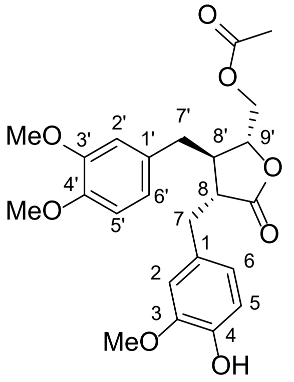

The reaction was carried out according to general procedure D using acetate **21** (26 mg, 0.0486 mmol) for 23 h to give the *title compound* **25** (14 mg, 64%) as an off-white solid. **m.p.** 32–34 °C.

δ_H (400 MHz; $CDCl_3$) 1.93 (3H, s, OCO<u>C</u>H_3), 2.19–2.27 (1H, m, H-8'), 2.64 (2H, d, *J* = 7.0 Hz, H-7'), 2.70 (1H, dt, *J* = 10.0, 5.5 Hz, H-8), 2.78 (1H, dd, *J* = 14.0, 5.5 Hz, H-7), 3.00 (1H, dd, *J* = 14.0, 5.5 Hz, H-7), 3.72 (1H, dd, *J* = 12.5, 5.8 Hz, 9'-CH_A), 3.827 (3-OCH_3), 3.830 (3'-OCH_3), 3.85 (4'-OCH_3), 4.01 (1H, dd, *J* = 12.5, 2.5 Hz, 9'-CH_B), 4.28 (1H, ddd, *J* = 8.5, 5.8, 2.5 Hz, H-9), 5.55 (1H, s, OH), 6.51 (1H, d, *J* = 2.0 Hz, H-6), 6.51 (1H, dd, *J* = 8.0, 2.0 Hz, H-2'), 6.59 (1H, dd, *J* = 8.0, 2.0 Hz, H-6'), 6.61 (1H, d, *J* = 2.0 Hz, H-2), 6.78 (1H, d, *J* = 8.0 Hz, H-5'), 6.81 (1H, d, *J* = 8.0 Hz, H-5);

δ_C (100 MHz; $CDCl_3$) 20.5 (OCO<u>C</u>H_3), 34.3 (C-7), 38.1 (C-7'), 41.6 (C-8), 47.1 (C-8'), 63.9 (9'-<u>C</u>H_2), 80.5 (C-9'), 111.4 (C-5'), 111.8 (C-2), 112.2 (C-2'), 114.1 (C-5), 121.0 (C-6), 122.4 (C-6'), 129.1 (C-1), 129.8 (C-1'), 144.6 (C-4'), 146.7 (C-4), 128.1 (C-3'), 149.2 (C-3), 170.5 (O<u>C</u>OCH_3), 177.2 (C-9);

IR ν_{max}(ATR)/cm^{-1}: 3443, 2934, 2850, 1769, 1724, 1514, 1140, 1125, 1025;

***m/z* (ESI$^+$)**: 467 (MNa^+, 100%); **HRMS** (ESI$^+$) Found (MNa^+): 467.1664; $C_{24}H_{28}NaO_8$ requires 467.1676.

((8*R**,8'*R**, 9'*R**)-8-(3',4'-Dimethoxybenzyl)-8'-(4-hydroxy-3-methoxybenzyl)-9'-oxotetrahydrofuran-9-yl)methyl propionate (26)

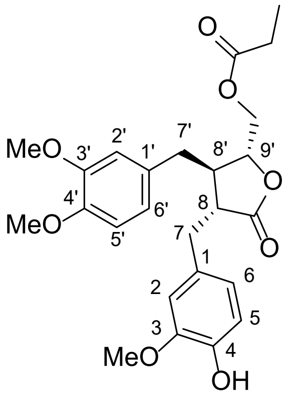

The reaction was carried out according to general procedure D using propionate **22** (13 mg, 0.0237 mmol) for 22 h to give the *title compound* **26** (9 mg, 81%) as a yellow oil.

δ_H (400 MHz; $CDCl_3$) 1.06 (3H, t, *J* = 7.5 Hz, -$OCOCH_2C\underline{H_3}$), 1.17–2.28 (3H, m, H-8', -$OCOC\underline{H_2}CH_3$), 2.65 (2H, d, *J* = 6.9 Hz, H-7'), 2.70 (1H, dt, *J* = 10.0, 5.5 Hz, H-8), 2.78 (1H, dd, *J* = 14.0, 5.5 Hz, H-7), 3.00 (1H, dd, *J* = 14.0, 5.5 Hz, H-7), 3.75 (1H, dd, *J* = 12.5, 5.8 Hz, 9'-CH_A), 3.82 (3H, s, 3-OCH_3), 3.83 (3H, s, 3'-OCH_3), 3.86 (3H, s, 4'-OCH_3), 4.05 (1H, dd, *J* =12.5, 2.5 Hz, 9'-CH_B), 4.29 (1H, ddd, *J* = 8.5, 5.8, 2.5 Hz, H-9'), 5.54 (2H, s, OH), 6.50–6.52 (2H, m, H-2', H-6), 6.60 (1H, dd, *J* = 8.0, 2.0 Hz, H-6'), 6.61 (1H, d, *J* = 2.0 Hz, H-2), 6.78 (1H, d, *J* = 8.0 Hz, H-5'), 6.80 (1H, d, *J* = 8.0 Hz, H-5);

δ_C (100 MHz; $CDCl_3$) 9.0 ($OCOCH_2\underline{C}H_3$), 27.3 ($OCO\underline{C}H_2CH_3$), 34.4 (C-7), 38.1 (C-7'), 41.7 (C-8'), 47.2 (C-8), 55.99 (4'-OCH_3), 56.03 (3-OCH_3 and 3'-OCH_3), 63.8 (9'-CH_2), 80.6 (C-9'), 111.5 (C-5'), 111.9 (C-2), 112.3 (C-2'), 114.2 (C-5), 121.1 (C-6'), 122.5 (C-6), 129.3 (C-1), 123.0 (C-1'), 144.8 (C-4), 146.8 (C-3), 148.2 (C-4'), 149.3 (C-3'), 174.1 ($O\underline{C}OCH_2CH_3$), 177.4 (C-9);

IR ν_{max}(ATR)/cm^{-1}: 3441, 2923, 2851, 1767, 1515, 1155, 1026;

***m/z* (ESI$^+$)**: 481 (MNa^+, 100%); **HRMS** (ESI$^+$) Found (MNa^+): 481.1815; $C_{25}H_{30}NaO_8$ requires 481.1833.

((8R^*,8'R^*,9'R^*)-8'-(3',4'-Dimethoxybenzyl)-8-(4-hydroxy-3-methoxybenzyl)-9'-oxotetrahydrofuran-9-yl)methyl benzoate (27)

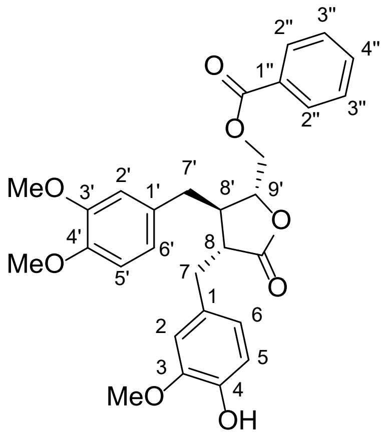

The reaction was carried out according to general procedure D using benzoate **23** (25 mg, 0.0419 mmol) for 24 h. The crude product was purified by flash chromatography (1:1 petroleum ether, ethyl acetate) to give the *title compound* **27** (21 mg, quant.) as a yellow oil.

R_f = 0.43 (1:1 petroleum ether, ethyl acetate);

δ_H (400 MHz; $CDCl_3$) 2.38–2.46 (1H, m, H-8'), 2.68 (1H, dd, *J* = 14.0, 7.5 Hz, 7'-H_A), 2.75 (1H, dt, *J* = 10.0, 5.5 Hz, H-8), 2.76 (1H, dd, *J* = 14.0, 4.7 Hz, 7'-H_B), 2.82 (1H, dd, *J* = 14.0, 5.5 Hz, 7-H_A) 3.07 (1H, dd, *J* = 14.0, 5.5 Hz, 7-H_B), 3.74 (3H, s, 3-OCH_3), 3.79 (3H, s, 3'-OCH_3), 3.84 (3H, s, 4'-OCH_3), 3.96 (1H, dd, *J* = 12.5, 5.5 Hz, 9'-CH_B), 4.32 (1H, dd, *J* = 12.5, 2.5 Hz, 9'-CH_A), 4.41 (1H, ddd, *J* = 9.0, 5.5, 2.5 Hz, H-9'), 5.51 (1H, s, 4-OH), 6.52 (1H, d, *J* = 2.0 Hz, H-2'), 6.54 (1H, dd, *J* = 8.0, 2.0 Hz, H-6), 6.62 (1H, dd, *J* = 8.5, 2.0 Hz, H-6'), 6.63 (1H, d, *J* = 2.0 Hz, H-2), 6.76 (1H, d, *J* = 8.0 Hz, H-5), 6.79 (1H, d, *J* = 8.0 Hz, H-5'), 7.43 (2H, t, *J* = 7.5 Hz, H-3''), 7.56 (1H, t, *J* = 7.5 Hz, H-4''), 7.83–7.85 (2H, m, H-2'');

δ_C (100 MHz; $CDCl_3$) 34.0 (C-7), 37.8 (C-7'), 41.6 (C-8'), 47.3 (C-8), 55.9 (3-OCH_3), 56.01 (3'-OCH_3 or 4'-OCH_3), 56.04 (3'-OCH_3 or 4'-OCH_3), 64.0 (9'-CH_2), 80.8 (C-9'), 111.5 (C-5), 112.0 (C-2), 112.2 (C-2'), 114.3 (C-5'), 121.1 (C-6'), 122.5 (C-6), 128.6 (C-3''), 129.1 (C-1''), 129.4 (C-1), 129.7 (C-2''), 129.9 (C-1'), 133.5 (C-4''), 144.7 (C-4), 146.8 (C-3'), 148.3 (C-4'), 149.4 (C-3), 166.1 (1''-COO), 177.3 (C-9);

IR ν_{max}(ATR)/cm^{-1}: 3425, 2936, 2843, 1768, 1717, 1514, 1174, 1140, 1124, 1026;

***m/z* (ESI$^+$)**: 529 (MNa^+, 100%); **HRMS** (ESI$^+$) Found (MNa^+): 529.1842; $C_{29}H_{30}NaO_8$ requires 529.1833.

(8*R,8'*R**,9'*R**)-9'-(Chloromethyl)-8'-(3',4'-dimethoxybenzyl)-8-(4-hydroxy-3-methoxybenzyl)dihydrofuran-9(8*H*)-one (33)**

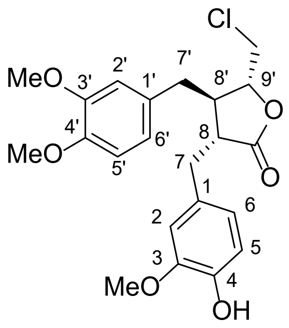

The reaction was carried out according to general procedure D using chloride **30** (20 mg, 0.0391 mmol) for 5 d to give the *title compound* **33** (4.6 mg, 29%) as a yellow oil.

δ_H (400 MHz; $CDCl_3$) 2.43–2.55 (2H, m, H-8', H-7'), 2.61–2.73 (2H, m, H-8, H-7'), 2.88–3.00 (3H, m, 9'-CH_A, H-7), 3.33 (1H, dd, *J* = 12.3, 3.8 Hz, 9'-CH_B), 3.83 (3H, s, 3-OCH_3 or 3'-OCH_3 or 4'-OCH_3), 3.85 (3H, s, 3-OCH_3 or 3'-OCH_3 or 4'-OCH_3), 3.86 (3H, s, 3-OCH_3 or 3'-OCH_3 or 4'-OCH_3), 4.31 (1H, dt, *J* = 7.2, 3.8 Hz, H-9'), 5.54 (1H, s, 4-OH), 6.48 (1H, d, *J* = 2.0 Hz, H-2'), 6.58 (1H, dd, *J* = 8.0, 2.0 Hz, H-6'), 6.64 (1H, dd, *J* = 8.0, 2.0 Hz, H-6), 6.68 (1H, d, *J* = 2.0 Hz, H-2), 6.77 (1H, d, *J* = 8.0 Hz, H-5'), 6.85 (1H, d, *J* = 8.0 Hz, H-5);

δ_C (100 MHz; $CDCl_3$) 34.9 (C-7), 38.9 (C-7'), 43.0 (C-8'), 45.3 (9'-CH_2), 47.6 (C-8), 56.02 (3-OCH_3 or 3'-OCH_3 or 4'-OCH_3), 56.03 (3-OCH_3 or 3'-OCH_3 or 4'-OCH_3), 56.09 (3-OCH_3 or 3'-OCH_3 or 4'-OCH_3), 81.9 (C-9'), 111.5 (C-5'), 111.8 (C-2 and C-2'), 114.4 (C-5), 120.8 (C-6'), 122.4 (C-6), 129.4 (C-1), 130.1 (C-1'), 144.8 (C-4), 146.9 (C-3), 148.3 (C-4'), 149.4 (C-3'), 177.0 (C-9);

IR ν_{max}(ATR)/cm^{-1}: 3426, 2921, 2851, 1766, 1514, 1153, 1023, 738;

***m/z* (ESI$^+$)**: 445 (^{37}ClMNa$^+$, 34%), 443 (^{35}ClMNa$^+$, 100%), 365 (56%); **HRMS** (ESI$^+$) Found (^{37}ClMNa$^+$): 445.1206; $C_{22}H_{25}{}^{37}ClNaO_6$ requires 445.1212. **HRMS** (ESI$^+$) Found (^{35}ClMNa$^+$): 443.1244; $C_{22}H_{25}{}^{35}ClNaO_6$ requires 443.1232.

(8*R,8'*R**,9'*S**)-8-(4-(Benzyloxy)-3-methoxybenzyl)-8'-(3',4'-dimethoxybenzyl)-9'-(hydroxymethyl)dihydrofuran-9(8*H*)-one (18)**

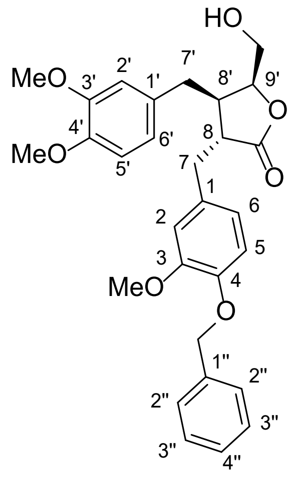

To diol *syn*-**17** (0.104 g, 0.179 mmol) in MeOH (3 mL) was added 2 M HCl (1.4 mL) and the resultant mixture heated at 70 °C for 4 h. The reaction mixture was then cooled to rt and sat. aq. $NaHCO_3$ (5 mL) added. The mixture was extracted with EtOAc (3 × 10 mL) and dried ($MgSO_4$). The solvent was removed *in vacuo* and the crude product was purified using flash chromatography (1:1 petroleum ether: ethyl acetate) to give the *title compound* **18** (83 mg, 94%) as a pale-yellow oil.

$\mathbf{R_f}$ = 0.16 (1:1 petroleum ether, ethyl acetate);

$\boldsymbol{\delta_H}$ (400 MHz; $CDCl_3$) 2.64–2.79 (4H, m, H-8', H_A-7, H-7'), 2.89–2.98 (2H, m, H-8, H_B-7), 3.79 (3H, s, 3'-OCH_3 or 4-OCH_3), 3.81 (3H, s, 3'-OCH_3 or 4-OCH_3), 3.83–3.86 (2H, m, 9'-CH_2), 3.85 (3H, s, 4'-OCH_3), 4.33 (1H, dt, *J* = 7.0, 3.0 Hz, H-9'), 5.11 (2H, s, 1''-CH_2), 6.47 (1H, dd, *J* = 8.0, 2.0 Hz, H-6), 6.56 (1H, d, *J* = 2.0 Hz, H-2'), 6.61 (1H, dd, *J* = 8.0, 2.0 Hz, H-6'), 6.62 (1H, d, *J* = 2.0 Hz, H-2), 6.75 (2H, d, *J* = 8.0 Hz, H-5, H-5'), 7.28 (1H, t, *J* = 7.3 Hz, H-4''), 7.35 (2H, t, *J* = 7.3 Hz, H-3''), 7.40–7.43 (2H, m, H-2'');

$\boldsymbol{\delta_C}$ (100 MHz; $CDCl_3$) 34.2 (C-7'), 34.6 (C-7), 41.8 (C-8'), 46.7 (C-8), 55.96 (3-OCH_3 or 3'-OCH_3 or 4'-OCH_3), 56.06 (3-OCH_3 or 3'-OCH_3 or 4'-OCH_3), 56.08 (3-OCH_3 or 3'-OCH_3 or 4'-OCH_3), 62.1 (9'-CH_2), 71.2 (1''-CH_2), 80.4 (C-9'), 111.5 (C-5'), 111.9 (C-2'), 113.2 (C-2), 114.1 (C-5), 120.5 (C-6), 121.7 (C-6'), 127.4 (C-2''), 128.0 (C-4''), 128.7 (C-3''), 130.7 (C-1'), 131.1 (C-1), 137.3 (C-1''), 147.2 (C-4), 147.9 (C-4'), 149.2 (C-3'), 149.8 (C-3), 179.0 (C-9);

IR ν_{max}(ATR)/cm^{-1}: 3522, 2928, 2869, 1762, 1514, 1139, 1025;

***m/z* (ESI$^+$)**: 515 (MNa^+, 100%); **HRMS** (ESI$^+$) Found (MNa^+): 515.2044; $C_{29}H_{32}NaO_7$ requires 512.2040.

((8*R,8'*R**,9'*S**)-8-(4-(Benzyloxy)-3-methoxybenzyl)-8'-(3',4'-dimethoxybenzyl)-9'-oxotetrahydrofuran-9-yl)methyl benzoate (24)**

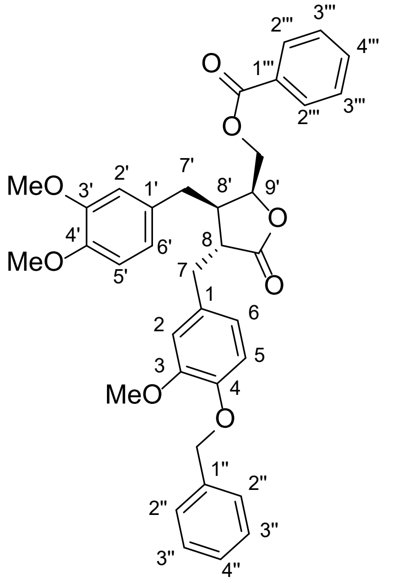

The reaction was carried out according to general procedure C with alcohol **19** (43 mg, 0.0873 mmol) and benzoyl chloride (0.01 mL, 0.0861 mmol) for 21 h. The crude product was purified using flash chromatography (3:1 petroleum ether, ethyl acetate) to give the *title compound* **24** (27 mg, 48%) as a pale-yellow solid. **m.p.** 46–49 °C.

R_f = 0.67 (1:1 petroleum ether, ethyl acetate);

δ_H (400 MHz; $CDCl_3$) 2.63–2.70 (2H, m, H-7'), 2.72–2.81 (1H, m, H-8'), 2.83–2.88 (2H, m, H-8, H_A-7), 2.99 (1H, dd, *J* = 15.6, 8.0 Hz, H_B-7), 3.79 (6H, s, 3-OCH_3, 3'-OCH_3), 3.85 (3H, s, 4'-OCH_3), 4.51 (1H, dd, *J* = 12.5, 3.3 Hz, 9'-CH_A), 4.56 (1H, dd, *J* = 12.5, 4.5 Hz, 9'-CH_B), 4.62 (1H, ddd, *J* = 7.5, 4.5, 3.3 Hz, H-9'), 5.12 (2H, s, 1''-CH_2), 6.50 (1H, d, *J* = 8.0, 2.0 Hz, H-6), 6.53 (1H, d, *J* = 2.0 Hz, H-2'), 6.58 (1H, dd, *J* = 8.0, 2.0 Hz, H-6'), 6.62 (1H, d, *J* = 2.0 Hz, H-2), 6.73 (1H, d, *J* = 8.0 Hz, H-5), 6.75 (1H, d, *J* = 8.0 Hz, H-5'), 7.27–7.31 (2H, m, H-4''), 7.33–7.37 (2H, m, H-3''), 7.41–7.43 (2H, m, H-2''), 7.46–7.50 (2H, m, H-3'''), 7.59–7.63 (1H, m, H-4'''), 8.00–8.03 (1H, m, H-2''');

δ_C (100 MHz; $CDCl_3$) 34.0 (C-7'), 34.6 (C-7), 41.9 (C-8'), 46.6 (C-8), 56.0 (4'-OCH_3), 56.1 (3-OCH_3 and 3'-OCH_3), 63.8 (9'-CH_2), 71.2 (1''-CH_2), 77.6 (C-9'), 111.5 (C-5), 111.8 (C-2'), 113.0 (C-2), 114.1 (C-5'), 120.5 (C-6'), 121.6 (C-6), 127.4 (C-2''), 128.0 (C-4''), 128.7 (C-3''), 128.8 (C-3'''), 129.5 (C-1'''), 129.9 (C-2'''), 130.2 (C-1'), 130.5 (C-1), 133.7 (C-4'''), 137.3 (C-1''), 147.3 (C-4), 148.0 (C-4'), 149.3 (C-3), 149.9 (C-3'), 166.1 (1'''-<u>C</u>O), 178.0 (C-9);

IR ν_{max}(ATR)/cm^{-1}: 2936, 2836, 1770, 1719, 1514, 1140, 1025;

***m/z* (ESI$^+$)**: 619 (MNa^+, 100%); **HRMS** (ESI$^+$) Found (MNa^+): 619.2279; $C_{36}H_{36}NaO_8$ requires 619.2302.

(8*R,8'*R**,9'*S**)-8'-(3',4'-Dimethoxybenzyl)-8-(4-hydroxy-3-methoxybenzyl)-9'-(hydroxymethyl)dihydrofuran-9(8*H*)-one (19)**

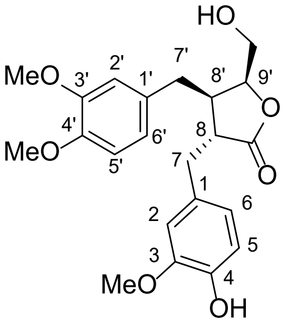

The reaction was carried out according to general procedure D using alcohol **18** (13 mg, 0.0264 mmol) for 19 h to give the *title compound* **19** (6 mg, 56%) as an off-white solid. **m.p.** 73–75 °C.

δ_H (400 MHz; $CDCl_3$) 1.96 (1H, s, 9'-CH_2OH), 2.62–2.70 (1H, m, H-8'), 2.72–2.80 (2H, m, H-7), 2.76 (1H, dd, *J* = 13.5, 5.0 Hz, H_A-7) 2.86–2.91 (1H, m, H-8), 2.95 (1H, dd, *J* = 13.5, 6.1 Hz, H_B-7), 3.79 (3H, s, 3-OCH_3), 3.81 (3H, s, 3'-OCH_3), 3.85–3.87 (5H, m, 4'-OCH_3, 9'-CH_2), 4.36 (1H, dt, *J* = 7.1, 3.6 Hz , C-9'), 5.52 (1H, s, 4-OH), 6.50 (1H, dd, *J* = 8.0, 2.0 Hz, H-6), 6.54 (2H, dd, *J* = 6.5, 2.0 Hz, H-2, H-2'), 6.63 (1H, dd, *J* = 8.0, 2.0 Hz, H-6'), 6.76 (1H, d, *J* = 8.0 Hz, H-5'), 6.78 (1H, d, *J* = 8.0 Hz, H-5);

δ_C (100 MHz; $CDCl_3$) 34.1 (C-7'), 34.6 (C-7), 41.7 (C-8'), 46.9 (C-8), 55.97 (2 x OCH_3), 56.05 (1 x OCH_3), 62.1 (9'-CH_2), 80.5 (C-9'), 111.4 (C-5'), 111.78 (C-2 or C-2'), 111.82 (C-2 or C-2'), 114.1 (C-5), 120.6 (C-6'), 122.5 (C-6), 129.4 (C-1), 131.1 (C-1'), 114.6 (C-3'), 146.8 (C-3), 147.9 (C-4'), 149.3 (C-3'), 179.0 (C-9);

IR ν_{max}(ATR)/cm^{-1}: 3402, 2937, 2840, 1753, 1515, 1261, 1154, 1025;

***m/z* (ESI$^+$)**: 425 (MNa^+, 100%); **HRMS** (ESI$^+$) Found (MNa^+): 425.1563; $C_{22}H_{26}NaO_7$ requires 425.1571.

(8R^*,8'R^*,9'S^*)-8'-(4-(Benzyloxy)-3-methoxybenzyl)-8-(3',4'-dimethoxybenzyl)-9'-(hydroxymethyl)dihydrofuran-9(8*H*)-one (28)

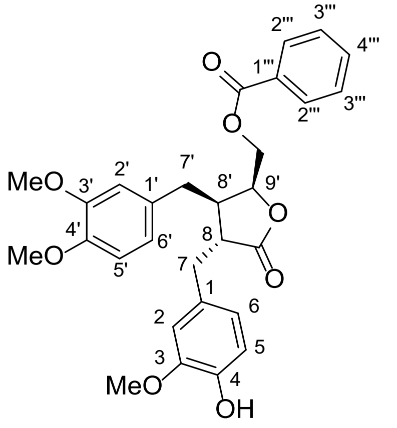

The reaction was carried out according to general procedure D using benzoate **24** (11 mg, 0.0184 mmol) for 19 h to give the *title compound* **28** (9 mg, quant.) as a pale yellow amorphous solid.

δ_H (400 MHz; $CDCl_3$) 2.66–2.70 (2H, m, H-7'), 2.72–2.78 (1H, m, H-8'), 2.81–2.88 (2H, m, H_A-7, H-8), 2.95–3.00 (1H, m, H_B-7), 3.77 (3H, s, 3-OCH_3), 3.80 (3H, s, 3'-OCH_3), 3.86 (3H, s, 4'-OCH_3), 4.55 (2H, app t, *J* = 4.3 Hz, 9'-CH_2), 4.64 (1H, ddd, *J* = 7.5, 4.3, 3.5 Hz, H-9'), 5.52 (1H, s, OH), 6.50 (2H, d, *J* = 2.0 Hz, H-2, H-2'), 6.53 (1H, dd, *J* = 8.0, 2.0 Hz, H-6), 6.59 (2H, dd, *J* = 8.5, 2.0 Hz, H-6'), 6.74 (1H, d, *J* = 8.0 Hz, H-5), 6.80 (1H, d, *J* = 8.5 Hz, H-5'), 7.46–7.50 (2H, m, H-3''), 7.59–7.63 (1H, m, H-4''), 8.00–8.04 (2H, m, H-2'');

δ_C (100 MHz; $CDCl_3$) 33.8 (C-7'), 34.3 (C-7), 41.6 (C-8'), 46.6 (C-8), 55.8 (3-OCH_3 and 3'-OCH_3), 55.9 (4'-OCH_3), 63.6 (9'-CH_2), 77.7 (C-9'), 111.3 (C-5'), 111.4 (C-2), 111.6 (C-2'), 114.0 (C-5), 120.4 (C-6'), 122.2 (C-6), 128.6 (C-3''), 129.0 (C-1), 129.3 (C-1''), 129.7 (C-2''), 130.0 (C-1'), 133.5 (C-4''), 144.6 (C-4), 146.7 (C-3), 147.9 (C-4'), 149.2 (C-3'), 166.0 (1''-C=O), 177.9 (C-9);

IR ν_{max}(ATR)/cm^{-1}: 3442, 2935, 2838, 1769, 1515, 1154, 1025;

***m/z* (ESI$^+$)**: 529 (MNa^+, 100%); **HRMS** (ESI$^+$) Found (MNa^+): 529.1715; $C_{22}H_{26}NaO_7$ requires 529.1838.

NMR spectra for precursors to compounds 3 and 6.

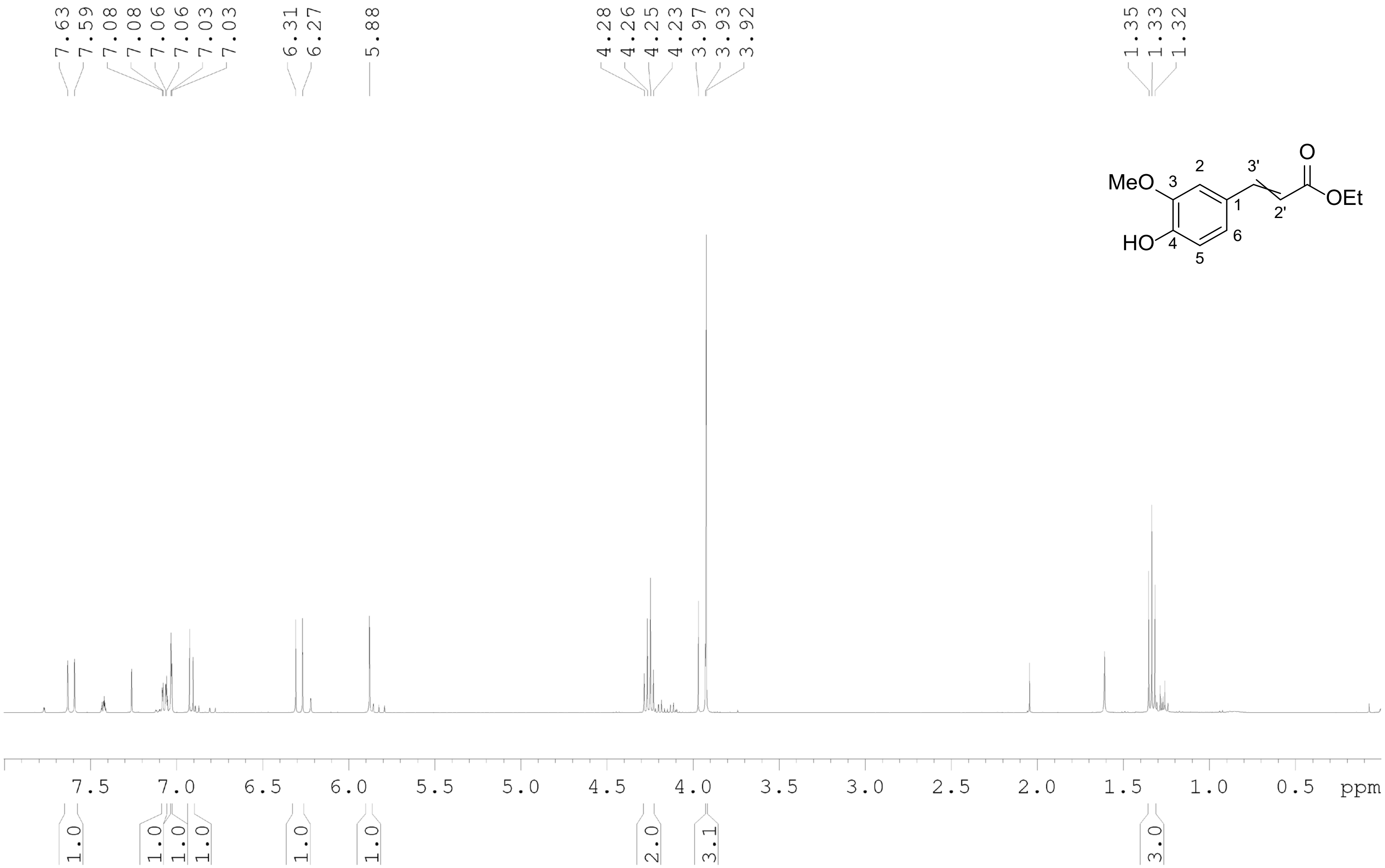

Figure S1. ^{1}H NMR spectrum of compound **7** (92:8 *E*:*Z*) in $CDCl_3$.

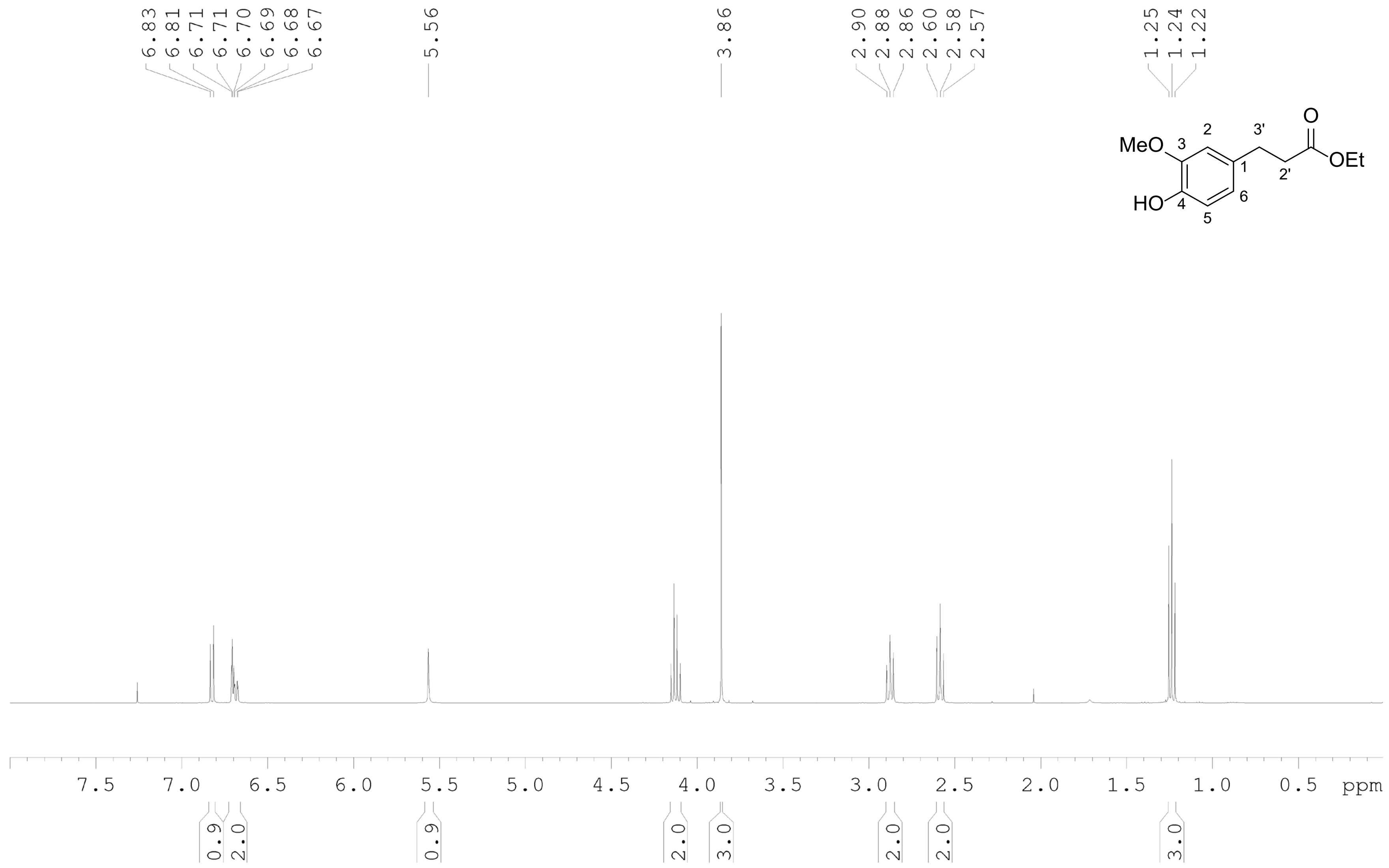

Figure S2. ^{1}H NMR spectrum of compound **8** in $CDCl_3$.

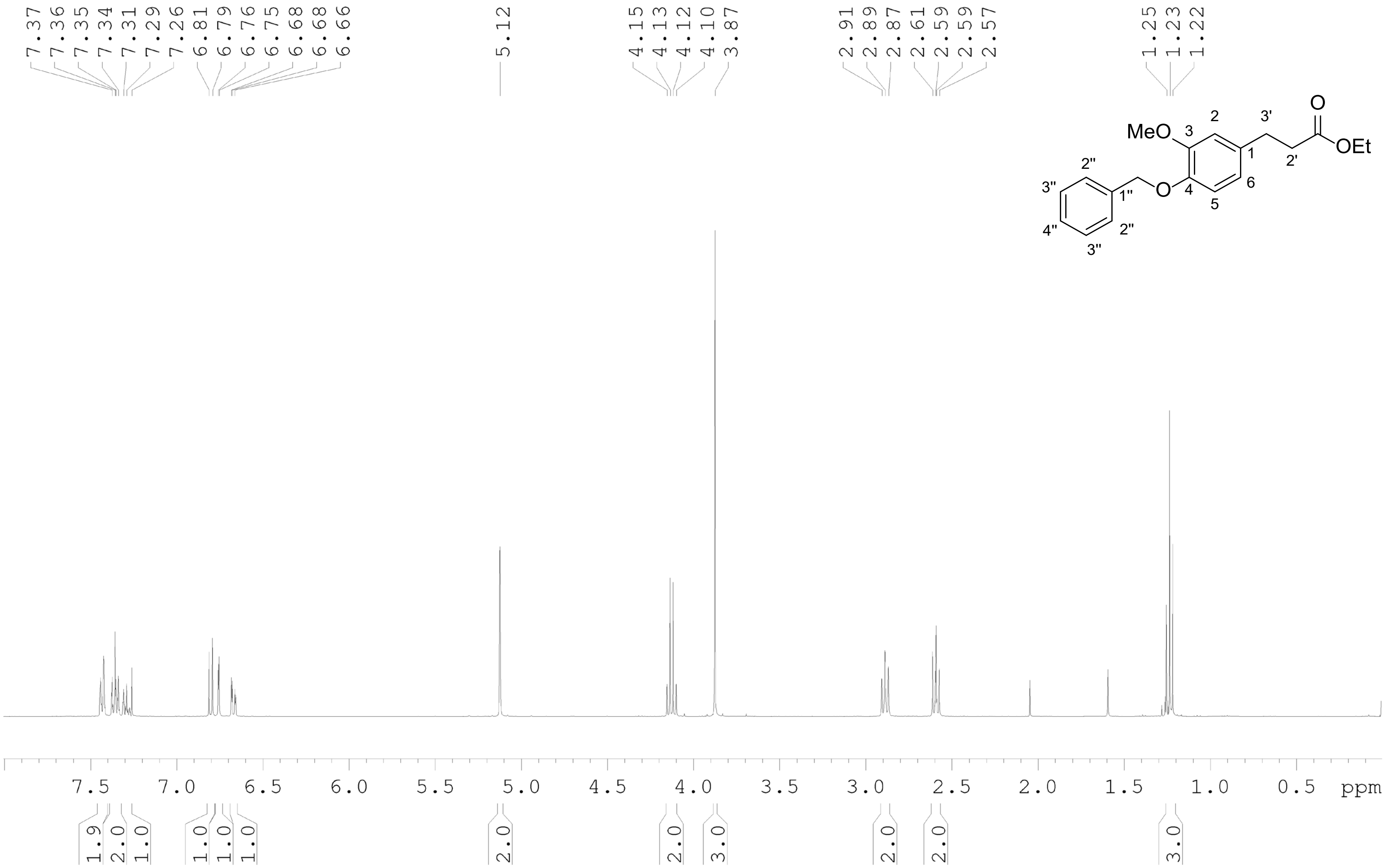

Figure S3. ^{1}H NMR spectrum of compound **9** in $CDCl_3$.

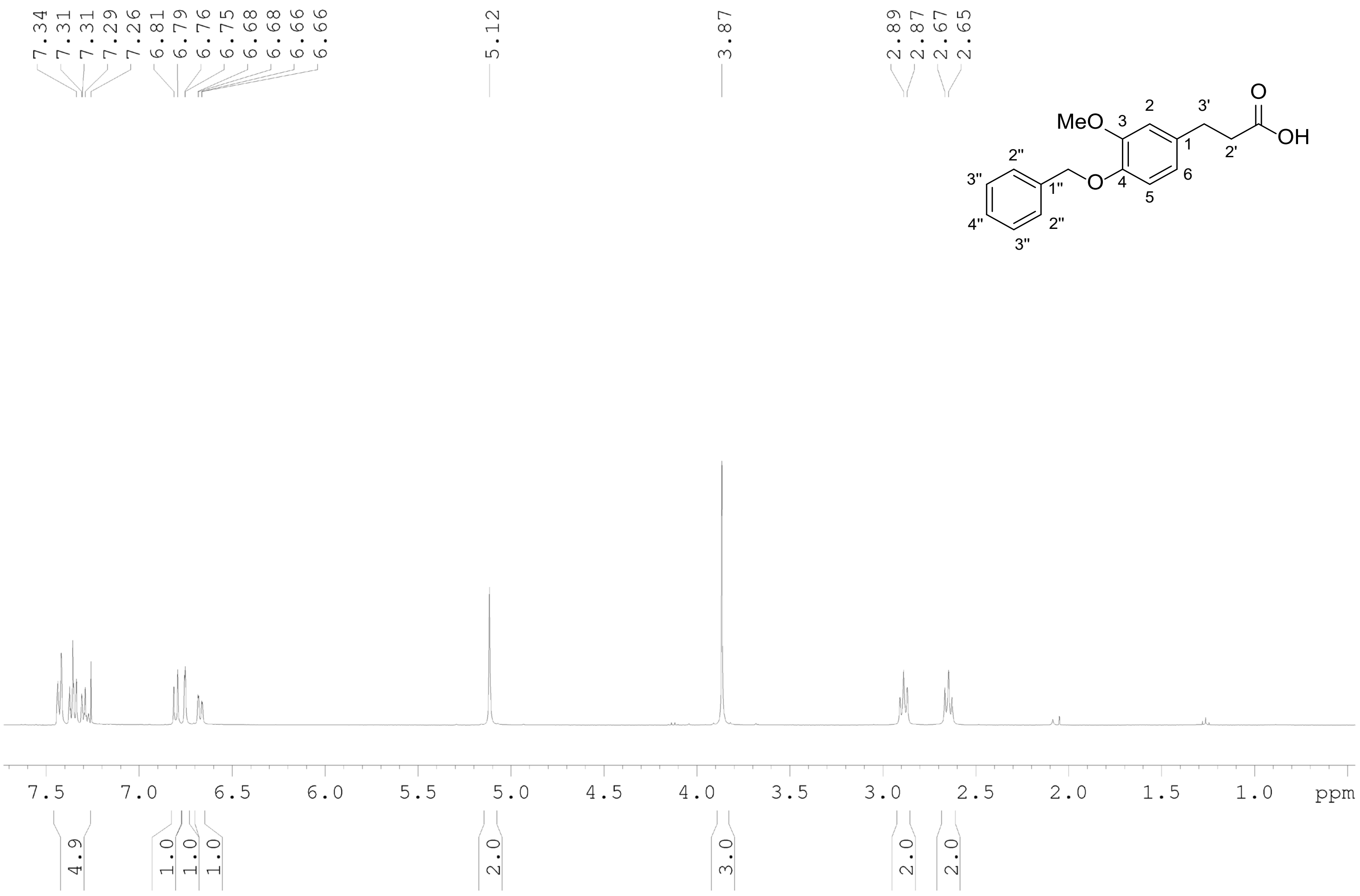

Figure S4. ^{1}H NMR spectrum of compound **6** in $CDCl_3$.

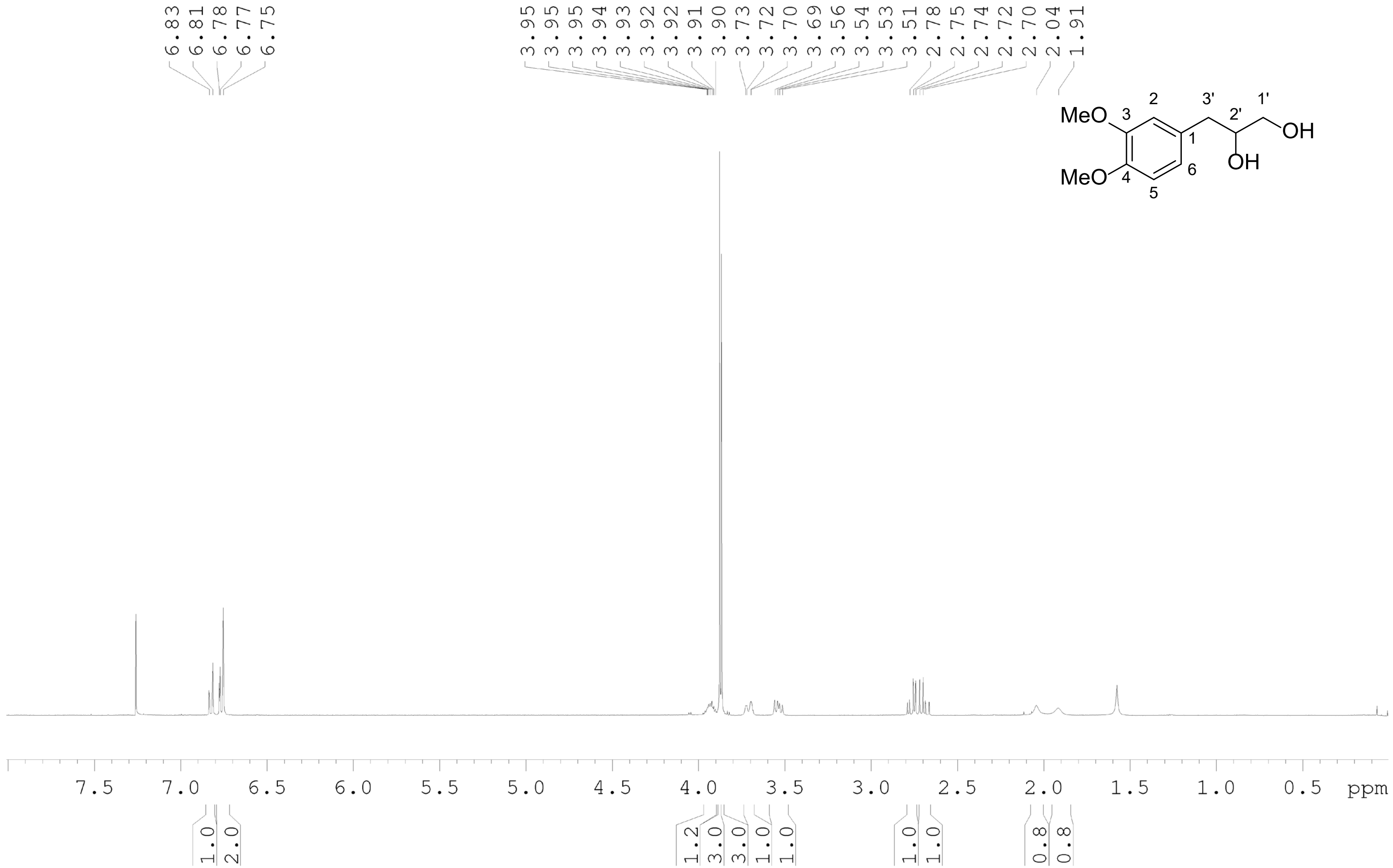

Figure S5. ^{1}H NMR spectrum of compound **10** in $CDCl_3$.

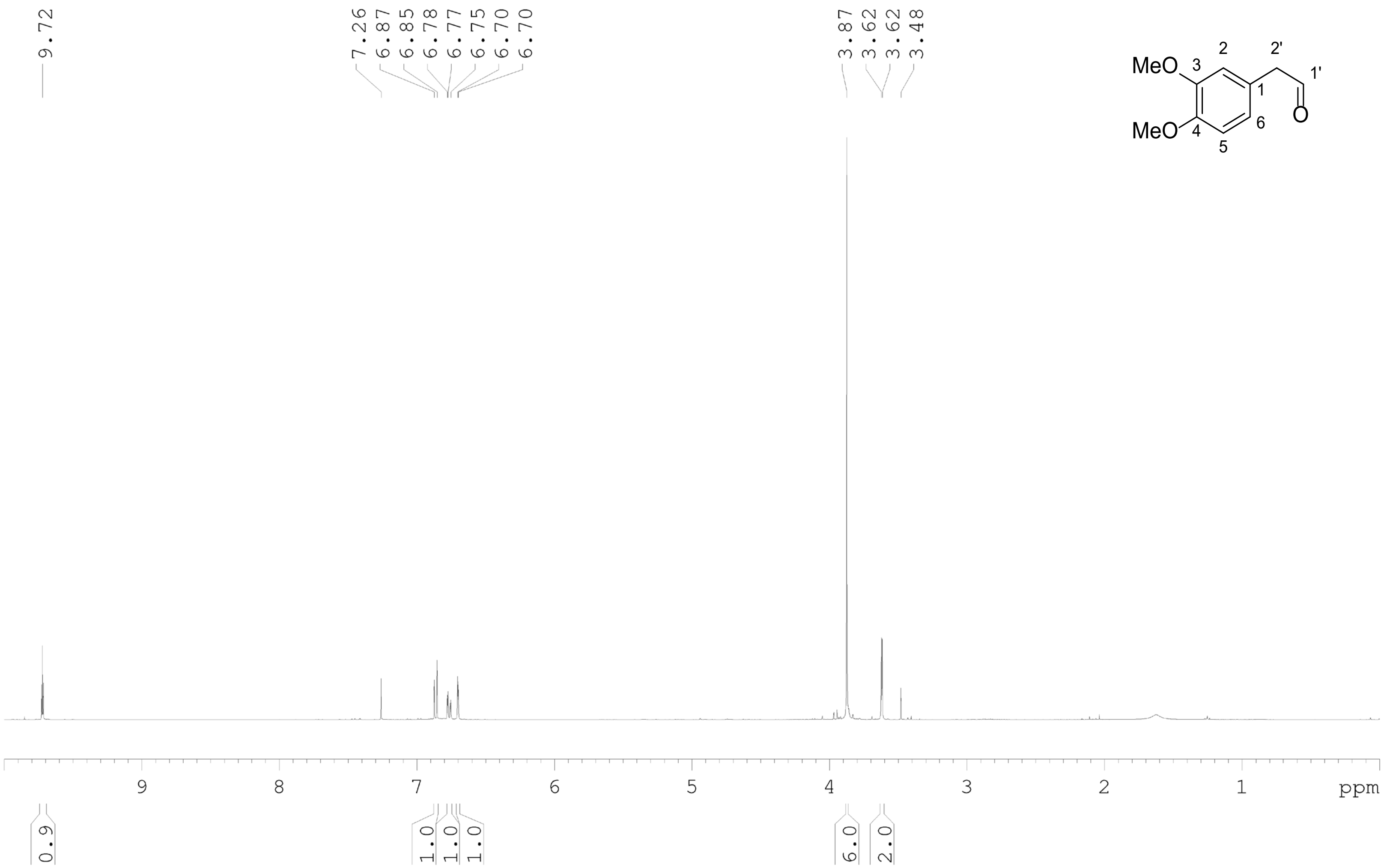

Figure S6. ^{1}H NMR spectrum of compound **11** in $CDCl_3$.

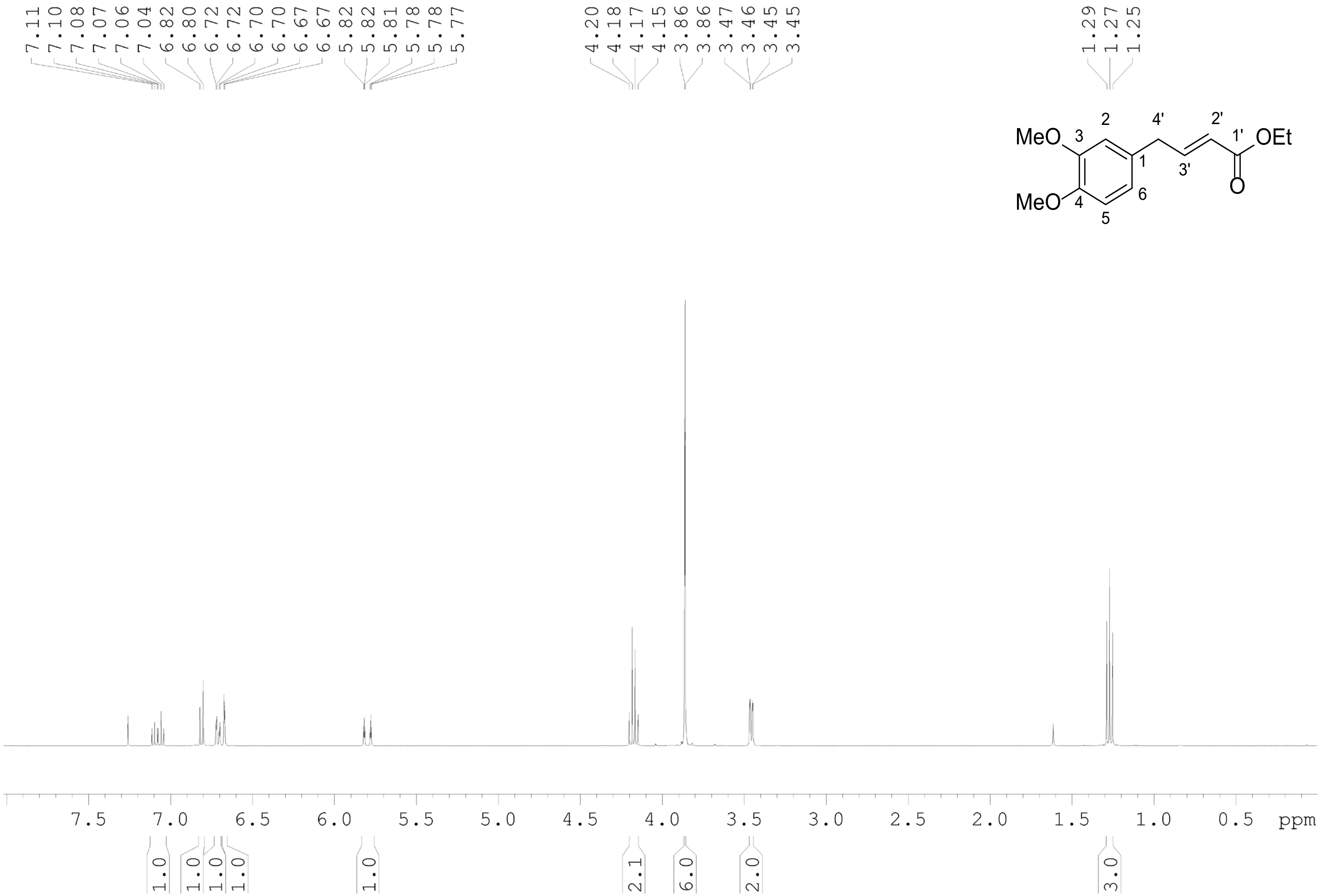

Figure S7. ^{1}H NMR spectrum of compound **12** in $CDCl_3$.

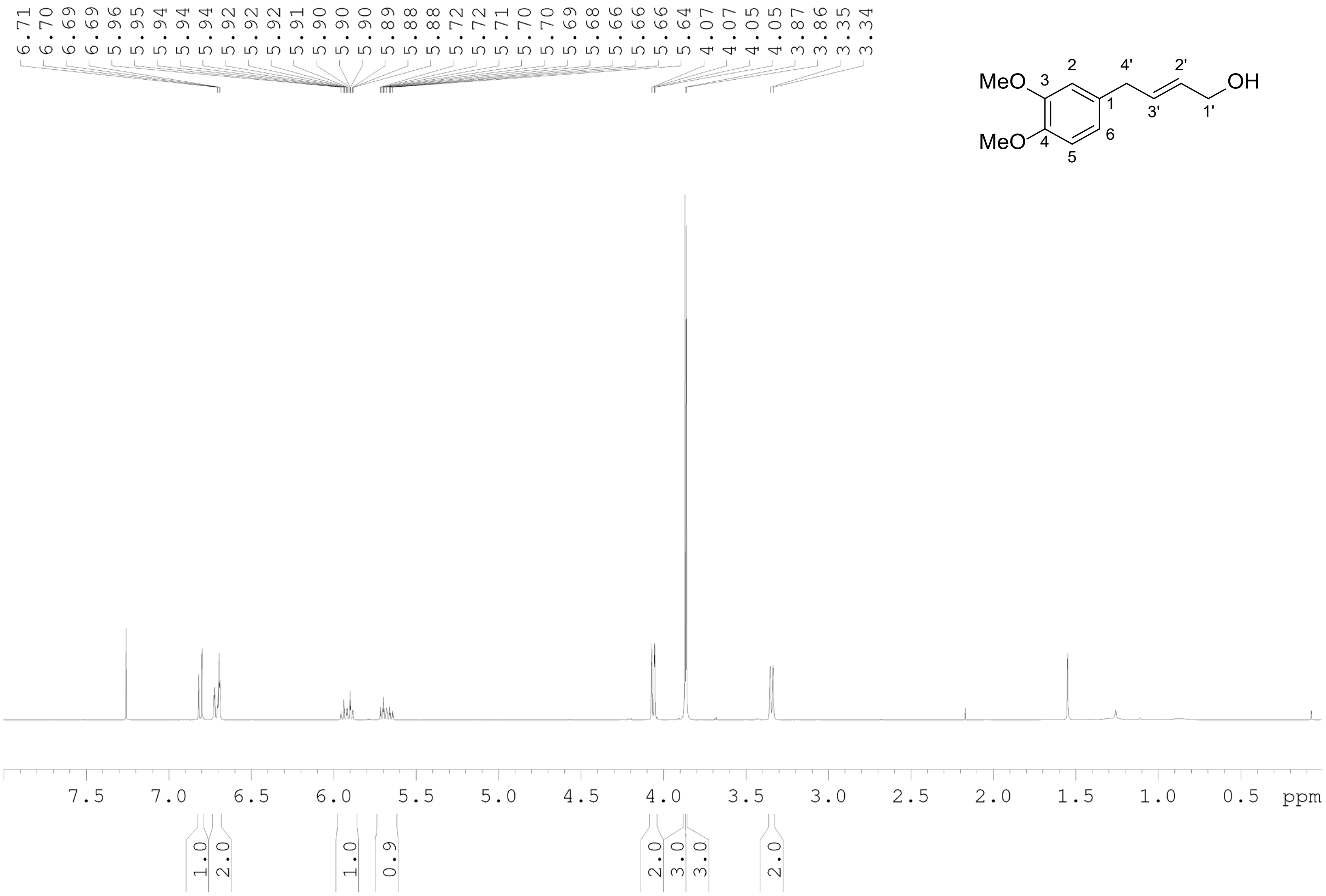

Figure S8. ^{1}H NMR spectrum of compound **14** in $CDCl_3$.

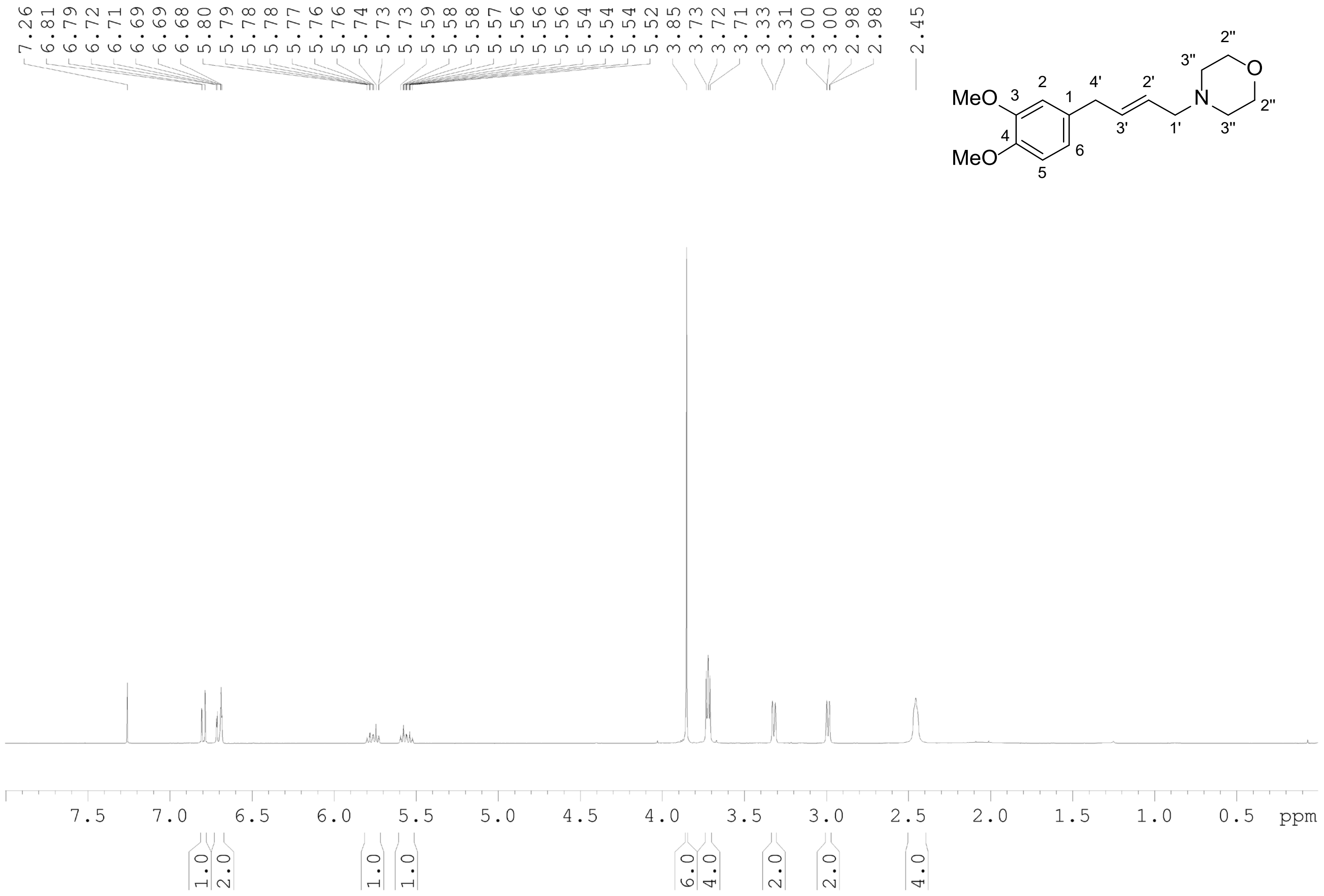

Figure S9. ^{1}H NMR spectrum of compound **3** in $CDCl_3$.

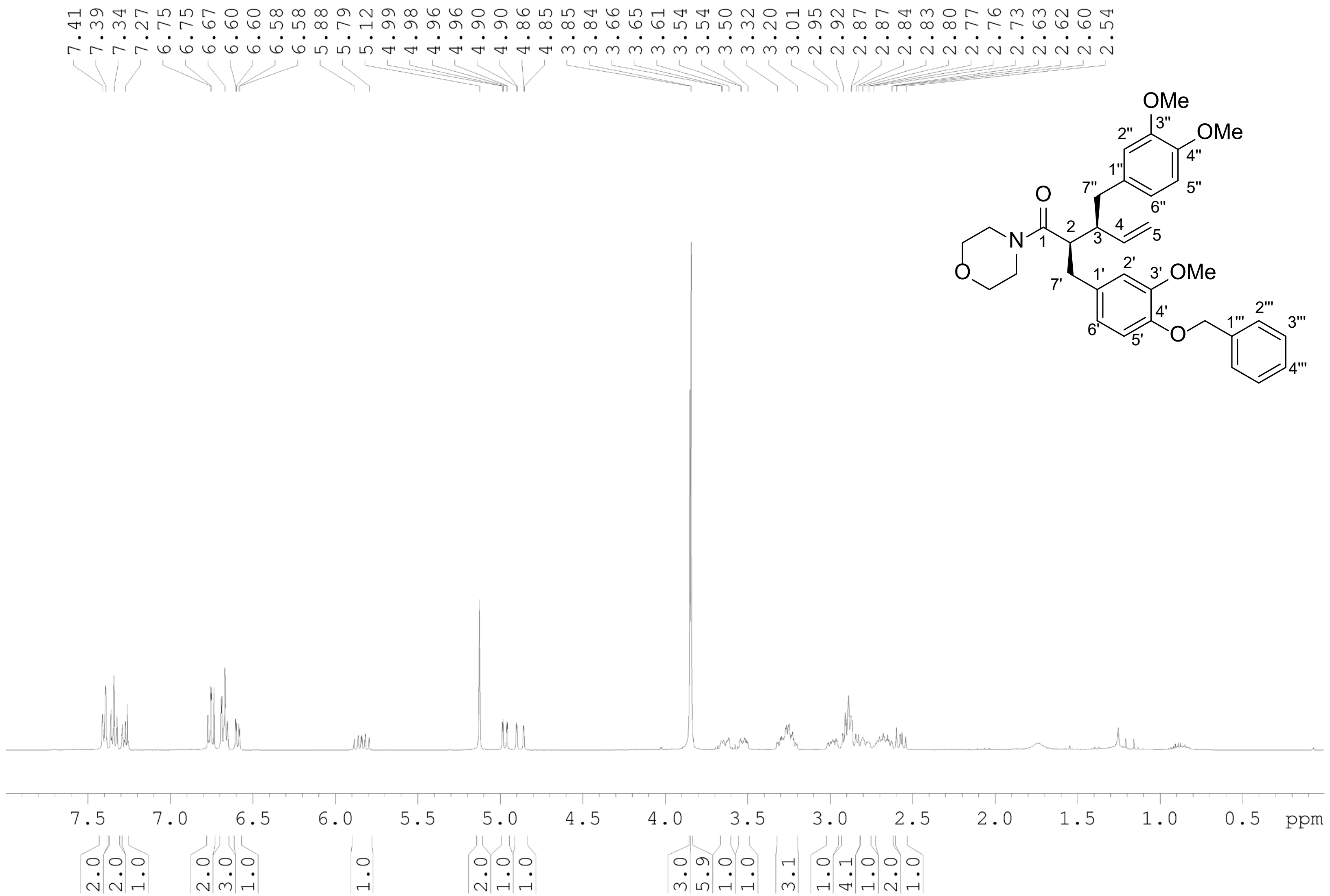

Figure S10. ^{1}H NMR spectrum of compound **1** in $CDCl_3$.

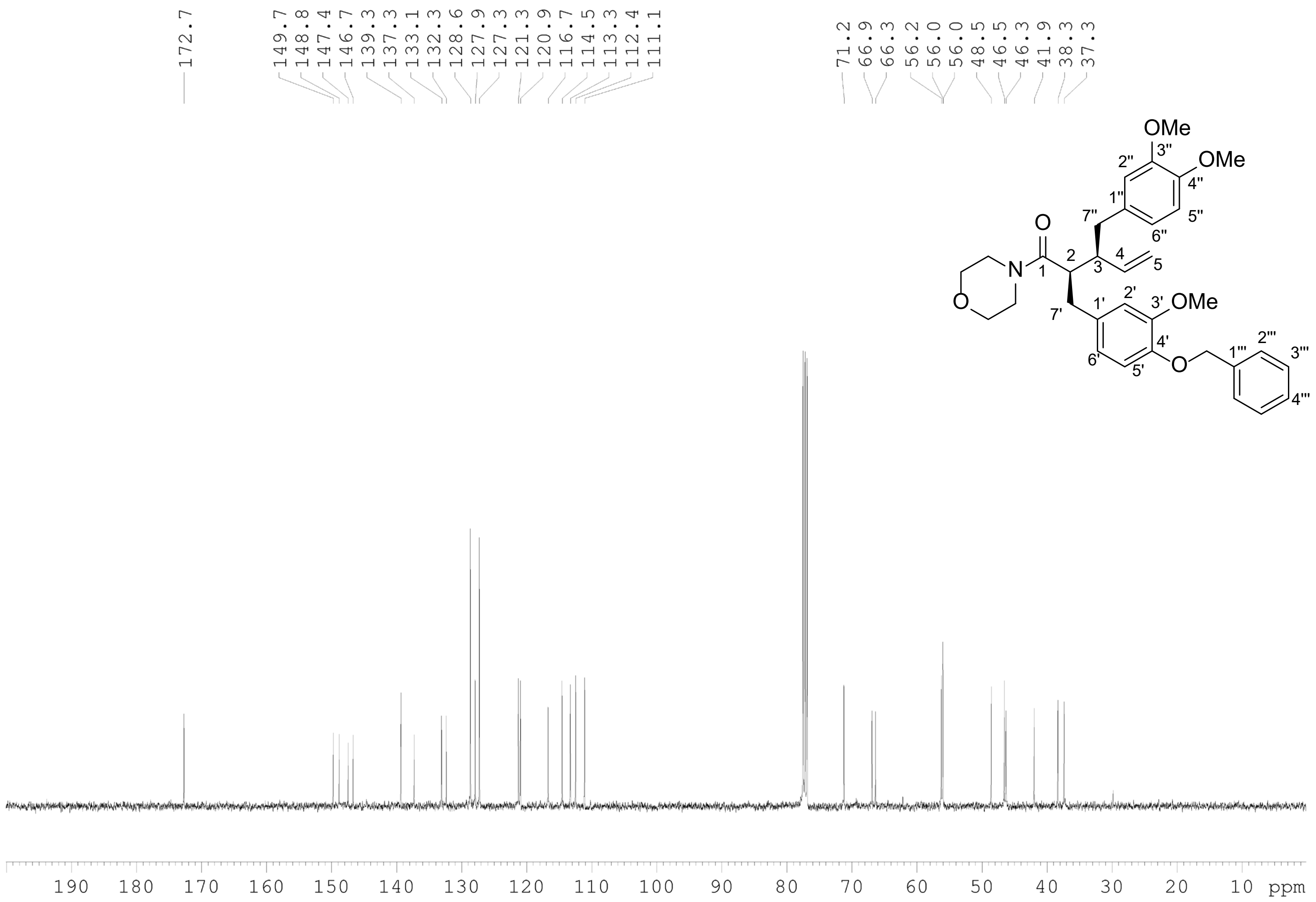

Figure S11. ^{13}C NMR spectrum of compound **1** in $CDCl_3$.

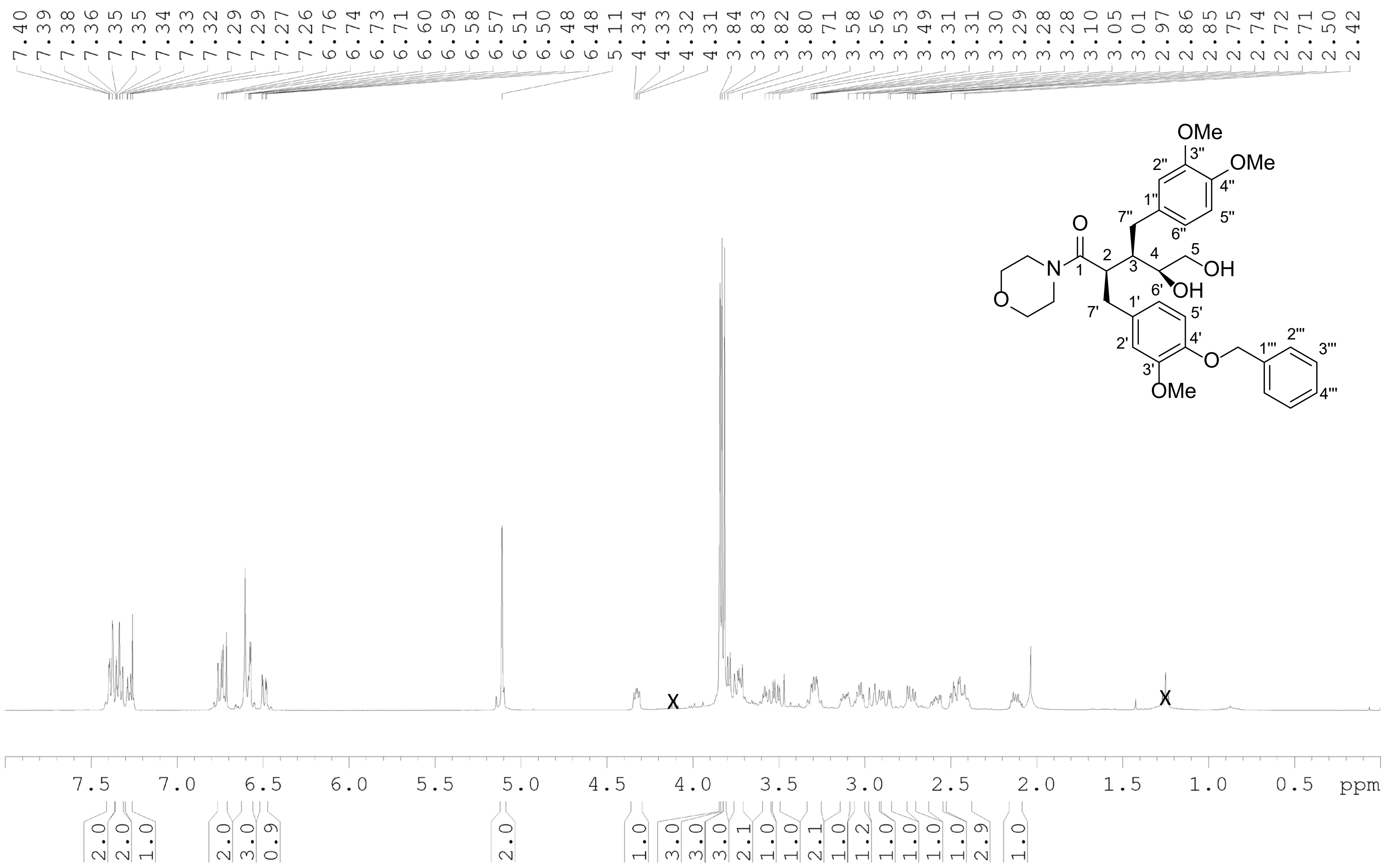

Figure S12. ^{1}H NMR spectrum of compound *syn*-**17** in $CDCl_3$. Crosses indicate solvent signals.

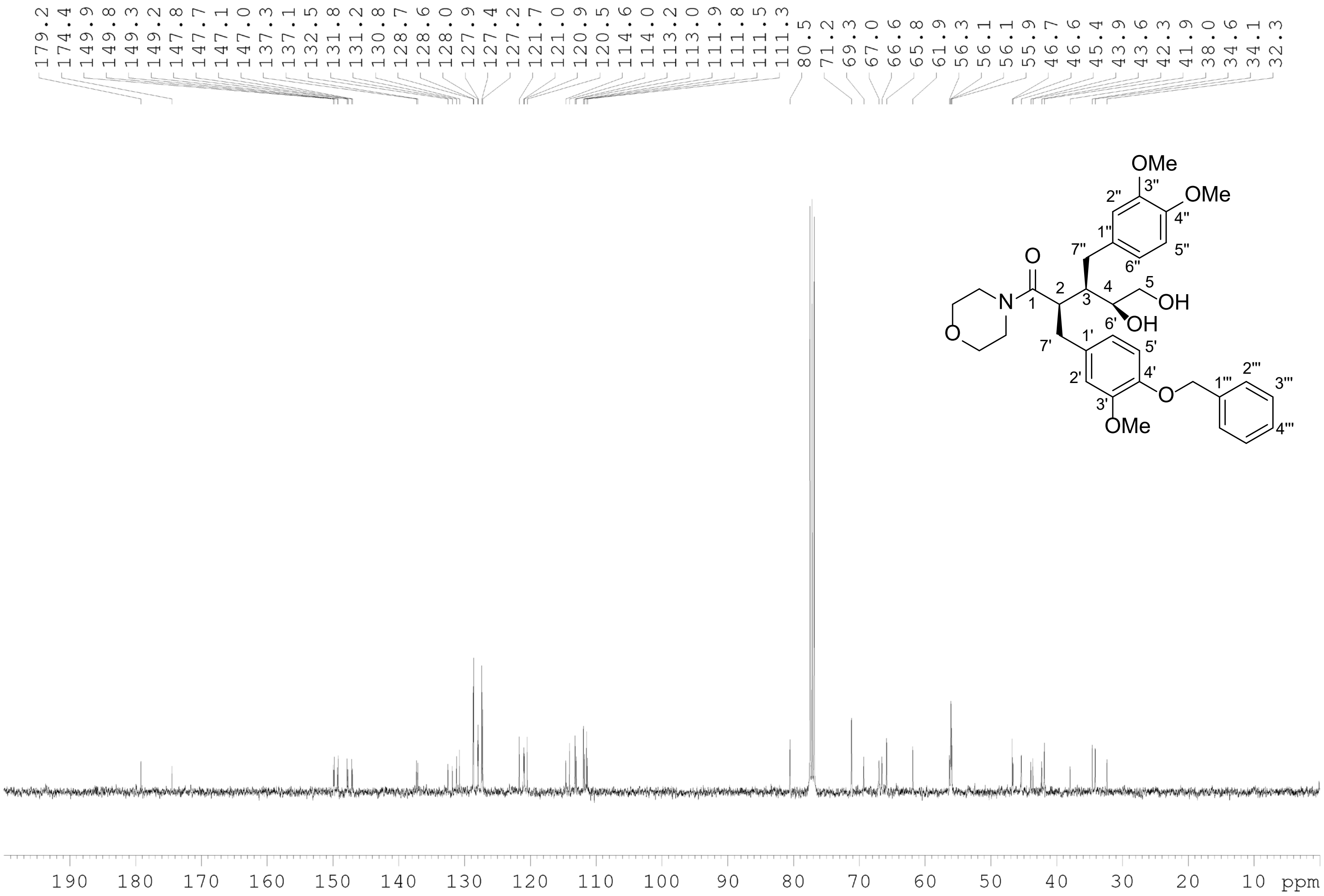

Figure S13. ^{13}C NMR spectrum of compound *syn*-**17** in $CDCl_3$.

NMR spectra of C-9’ lactone compounds

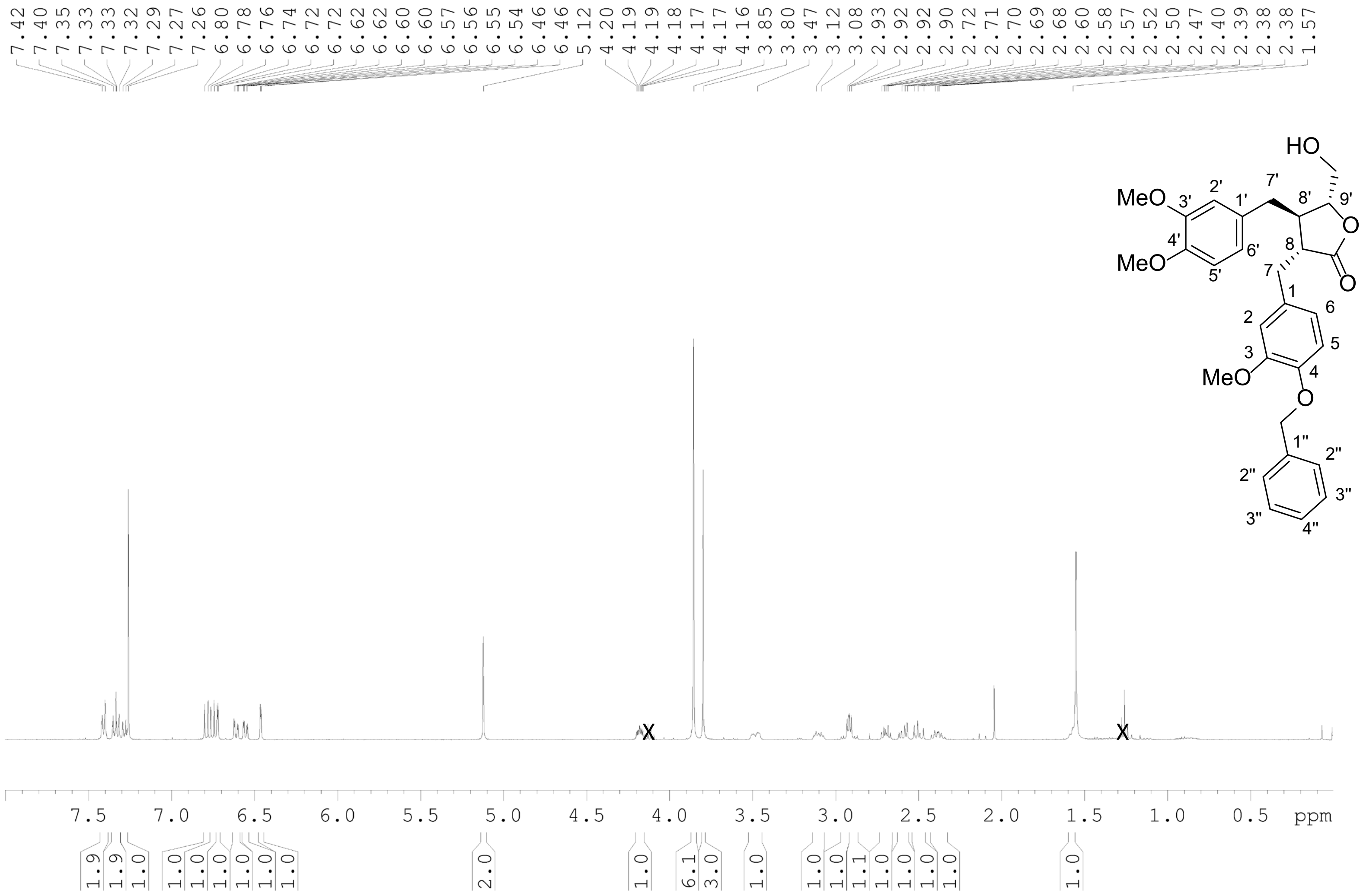

Figure S14. ^{1}H NMR spectrum of compound **16** in $CDCl_3$. Crosses indicate solvent signals.

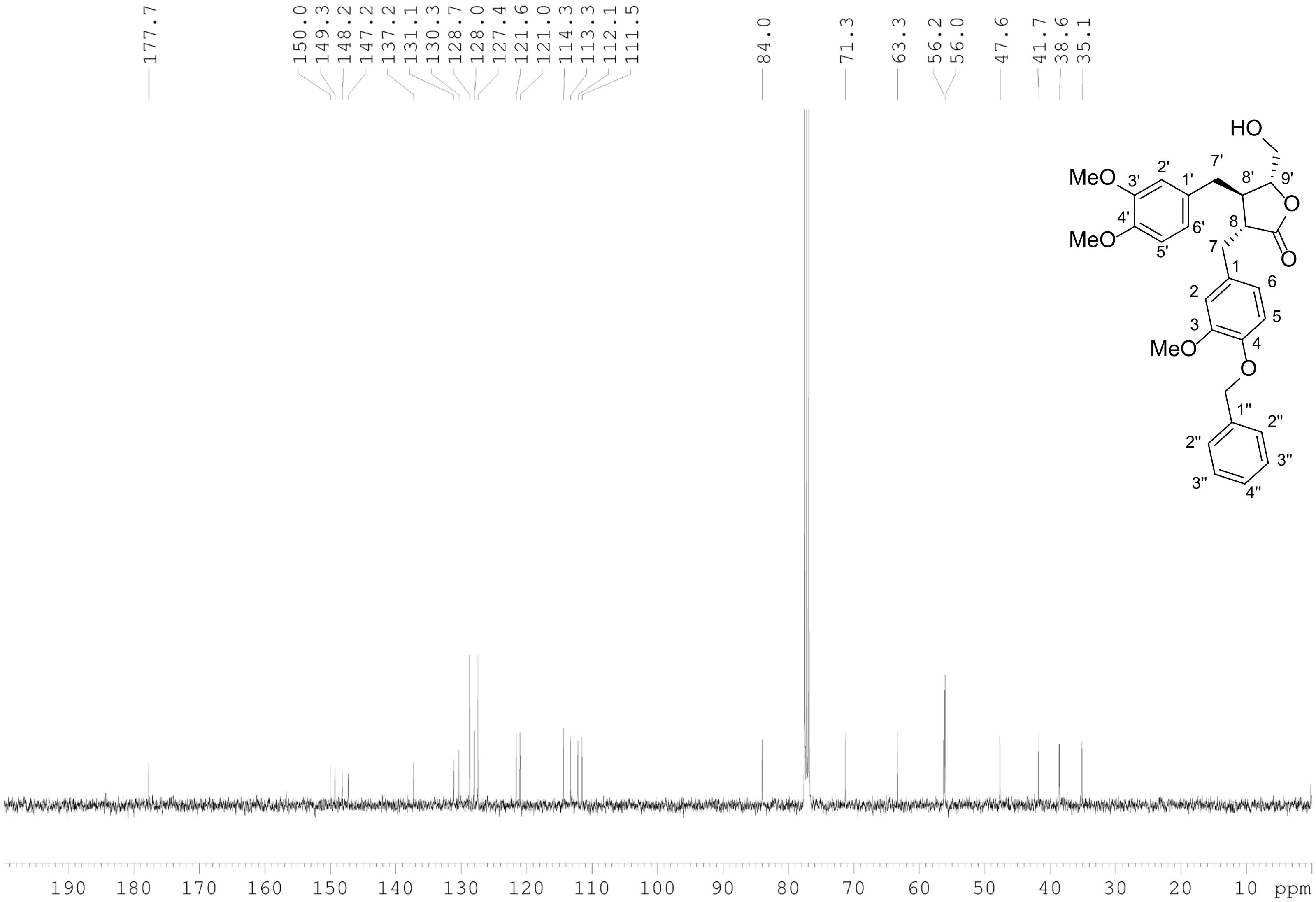

Figure S15. ^{13}C NMR spectrum of compound **16** in $CDCl_3$.

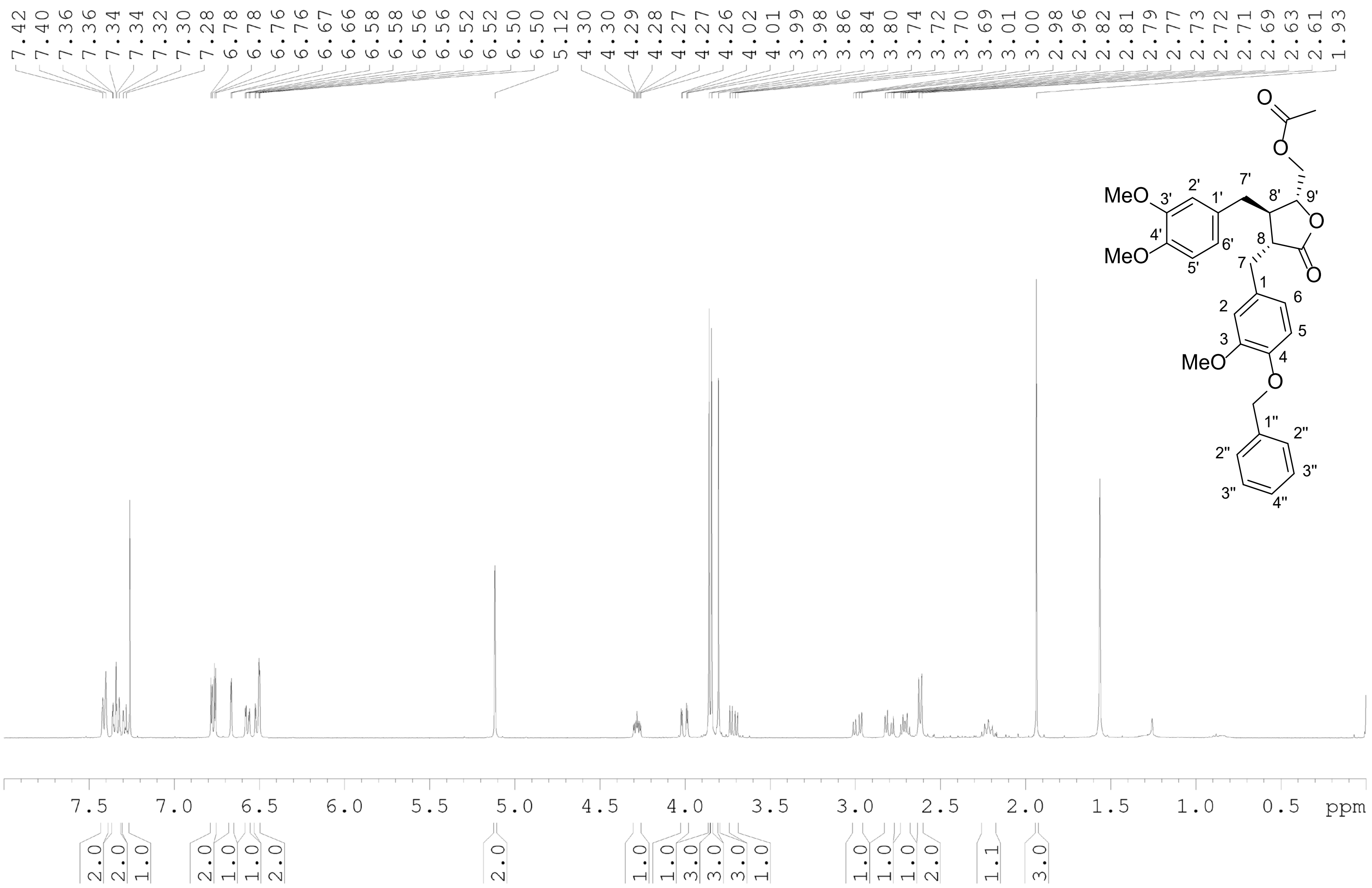

Figure S16. ^{1}H NMR spectrum of compound **21** in $CDCl_3$.

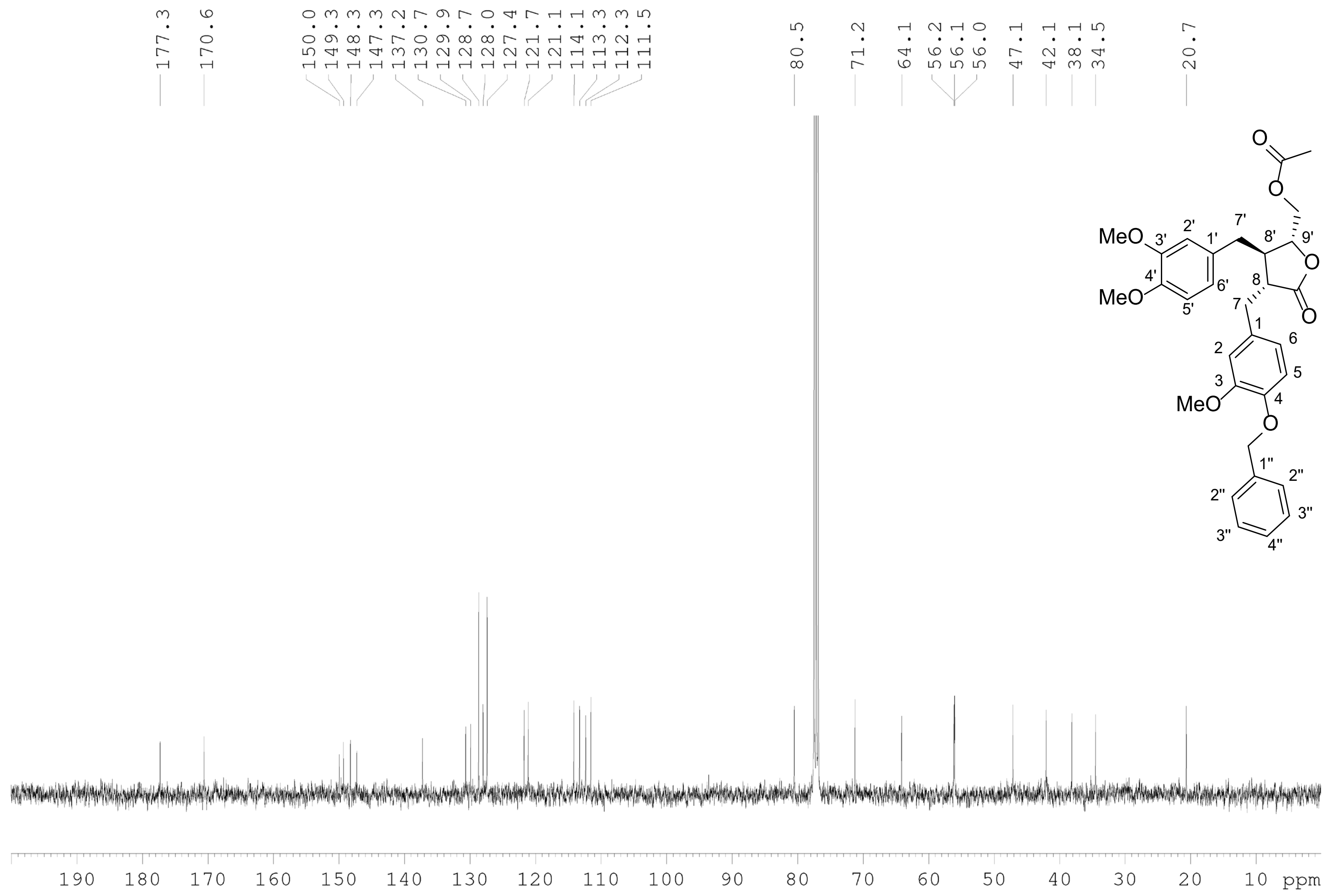

Figure S17. ^{13}C NMR spectrum of compound **21** in $CDCl_3$.

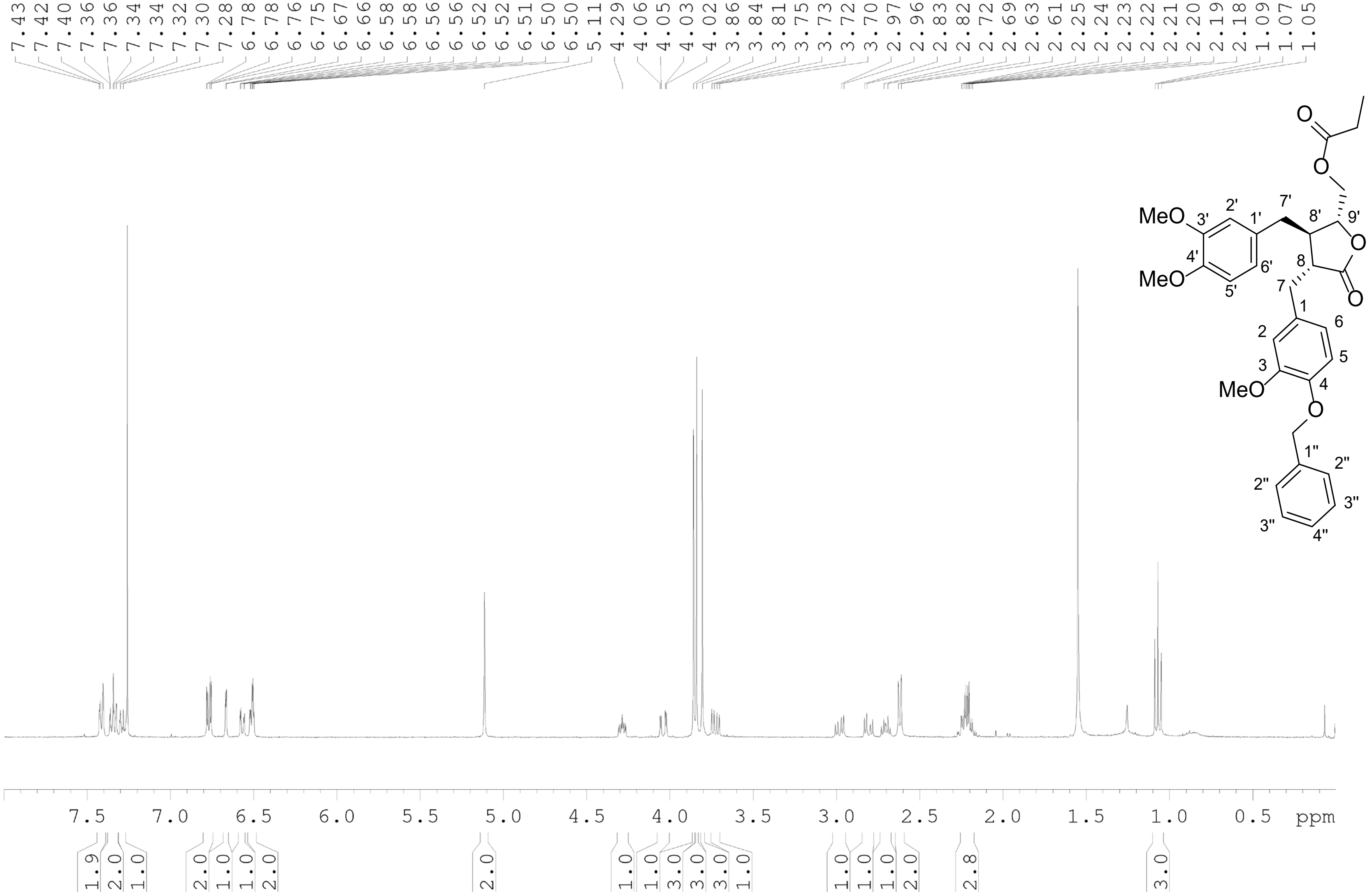

Figure S18. ^{1}H NMR spectrum of compound **22** in $CDCl_3$.

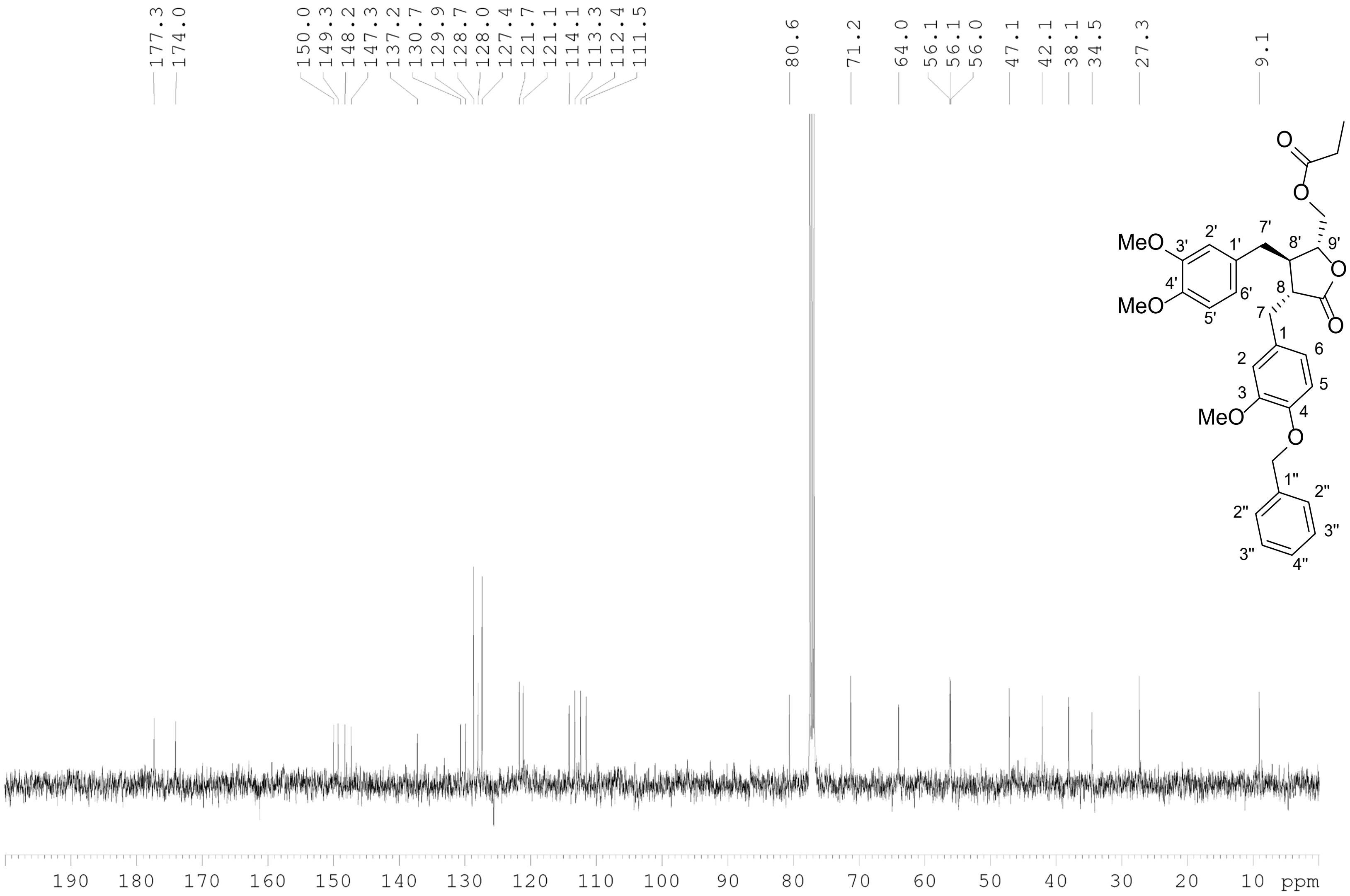

Figure S19. ^{13}C NMR spectrum of compound **22** in $CDCl_3$.

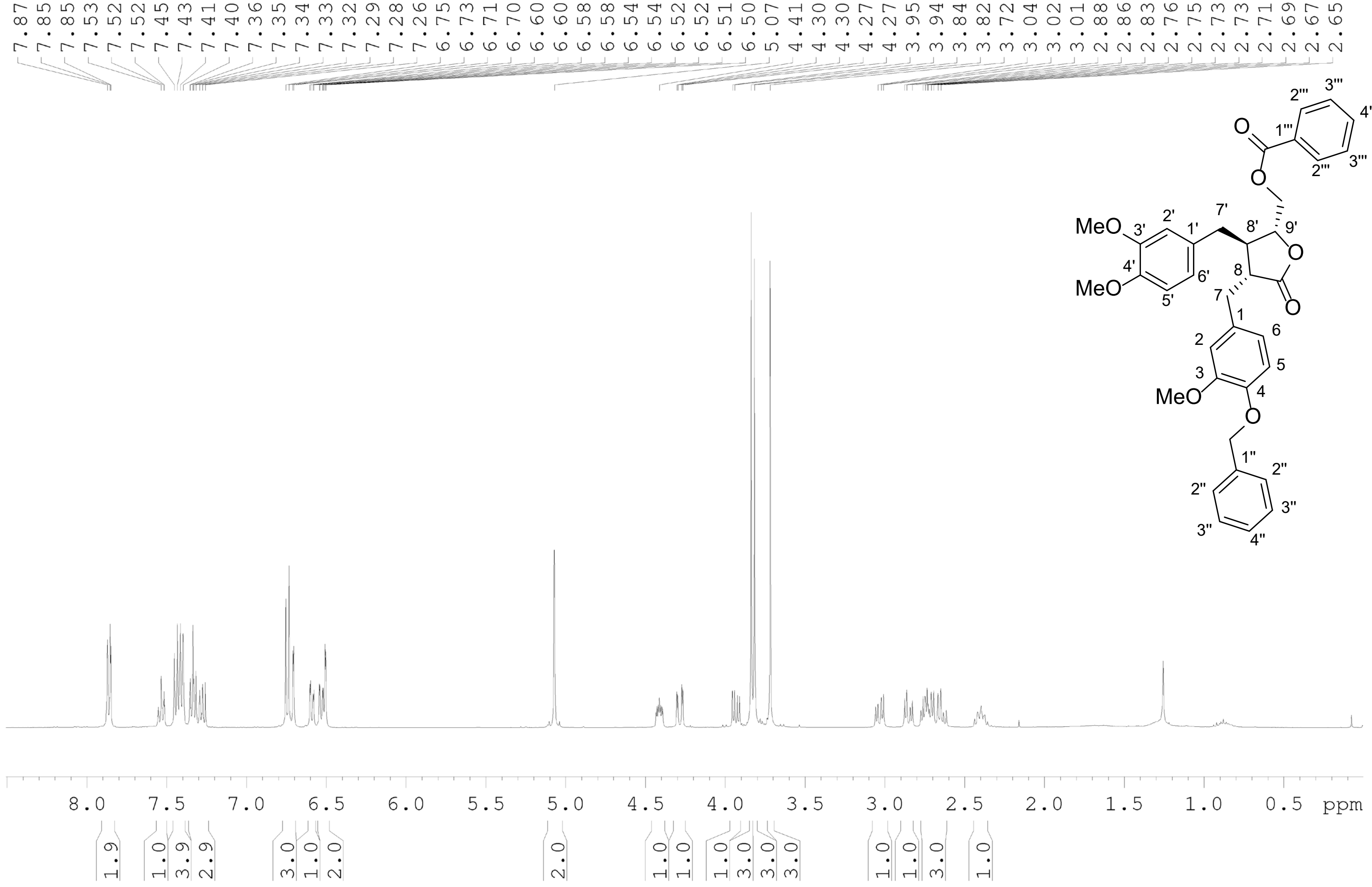

Figure S20. ^{1}H NMR spectrum of compound **23** in $CDCl_3$.

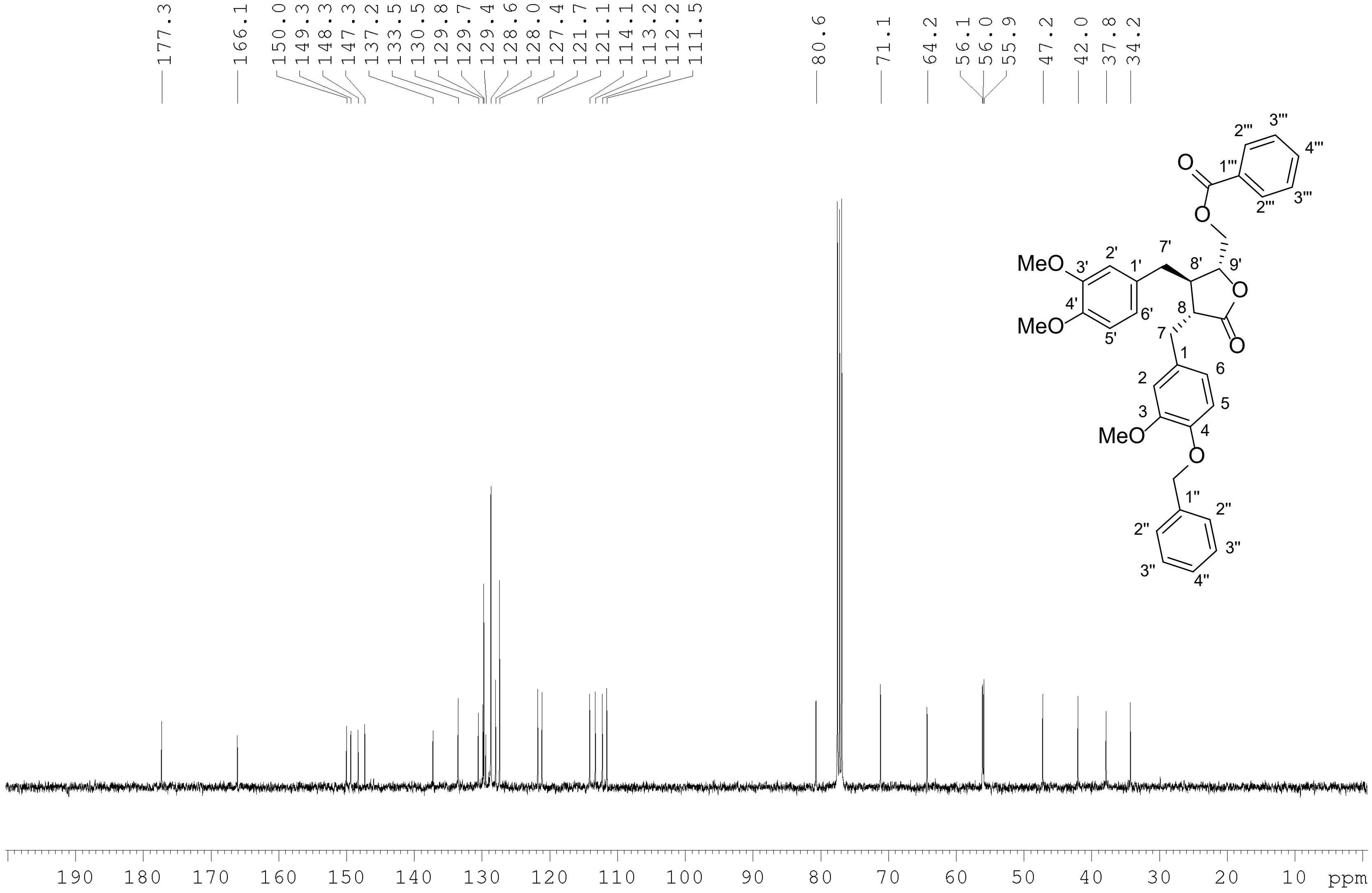

Figure S21. ^{13}C NMR spectrum of compound **23** in $CDCl_3$.

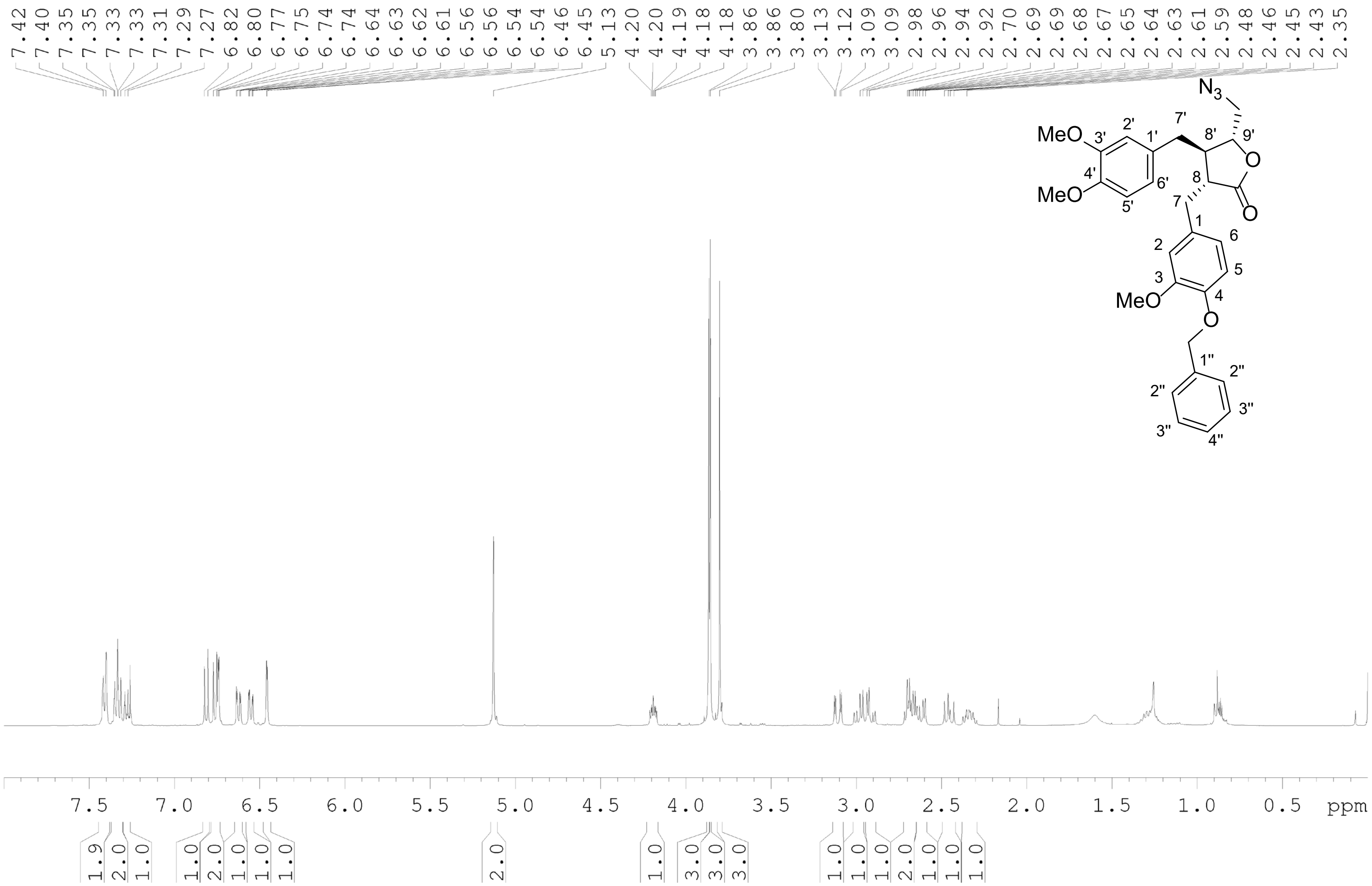

Figure S22. ^{1}H NMR spectrum of compound **29** in $CDCl_3$.

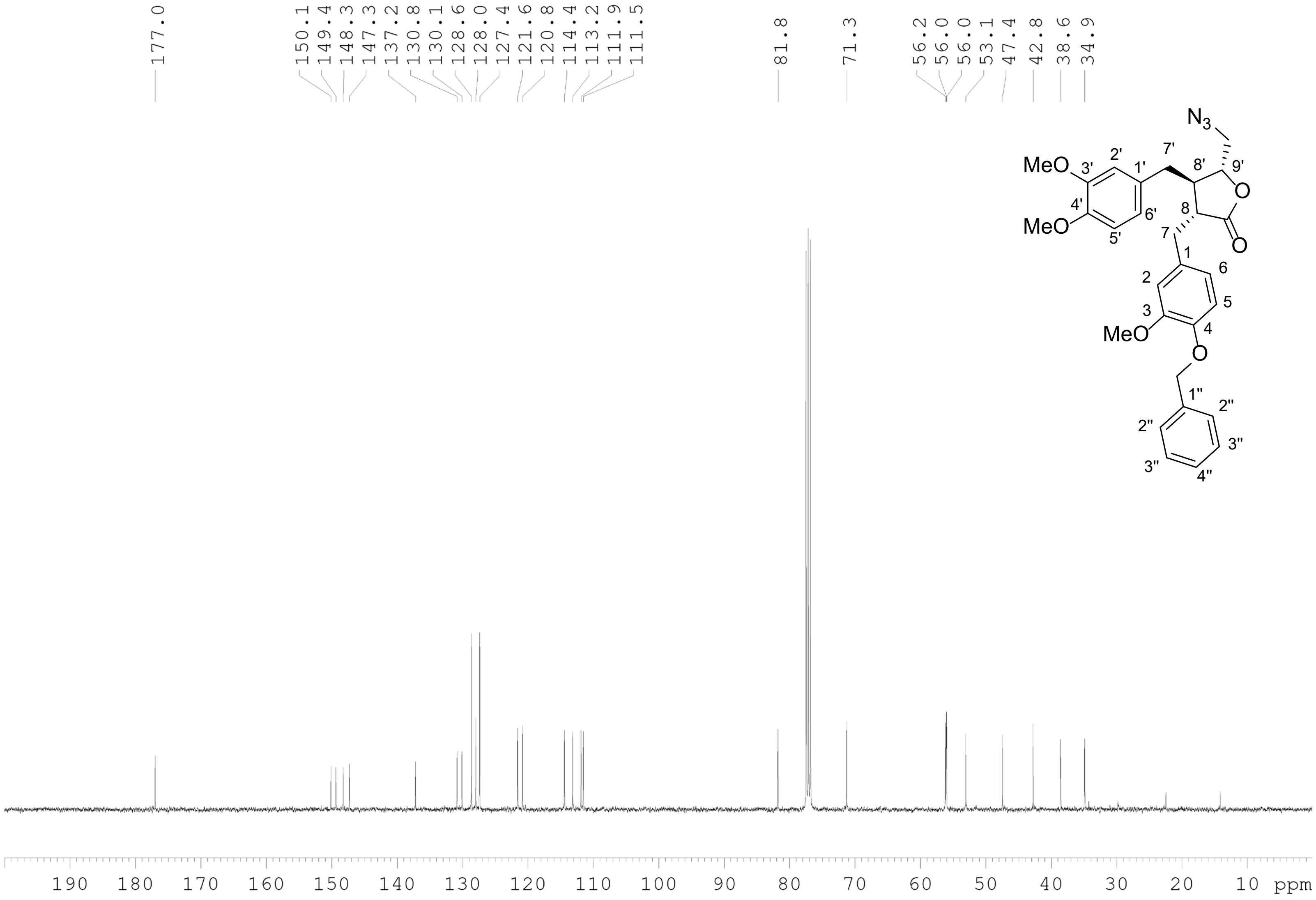

Figure S23. ^{13}C NMR spectrum of compound **29** in $CDCl_3$.

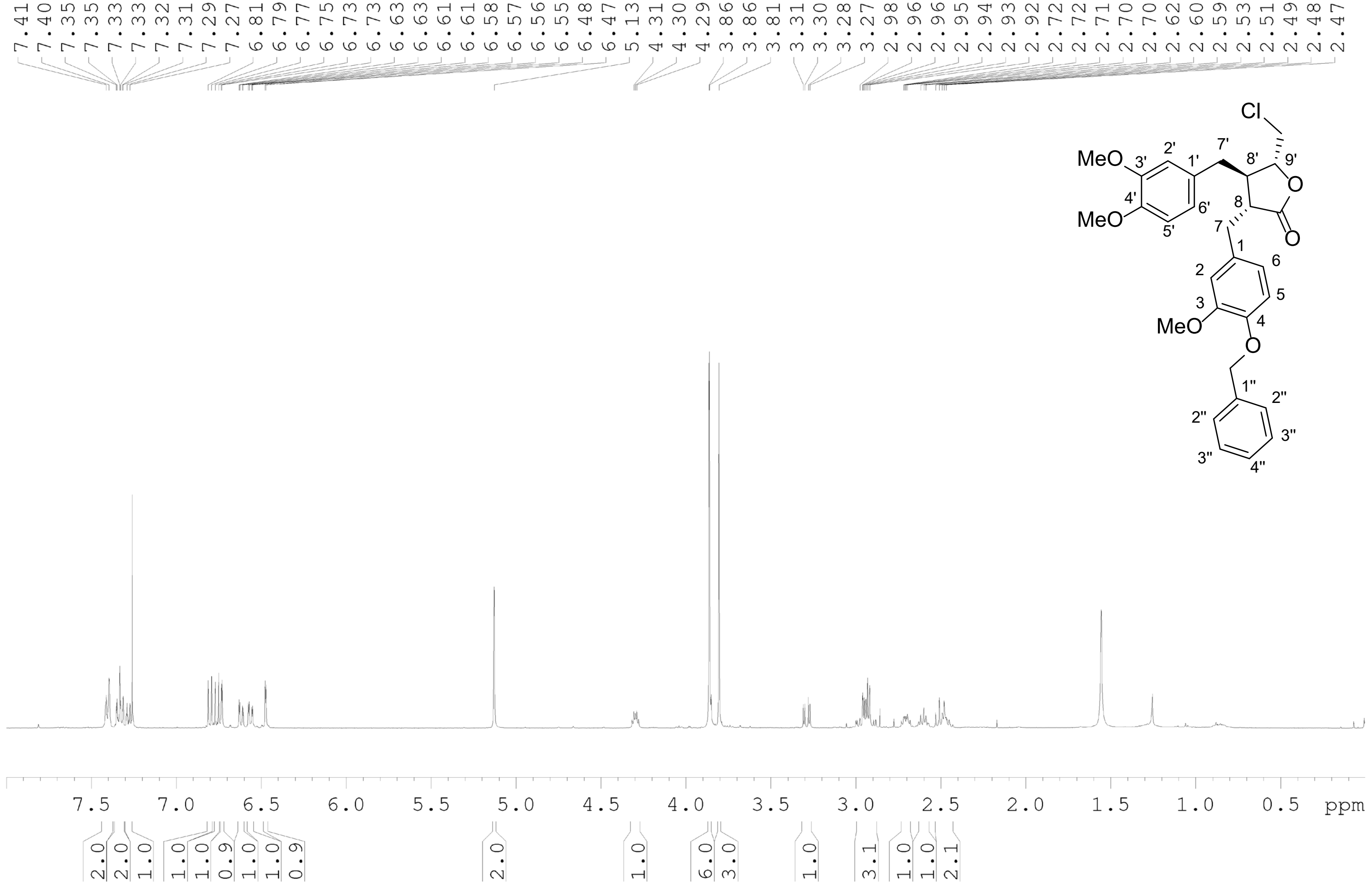

Figure S24. ^{1}H NMR spectrum of compound **30** in $CDCl_3$.

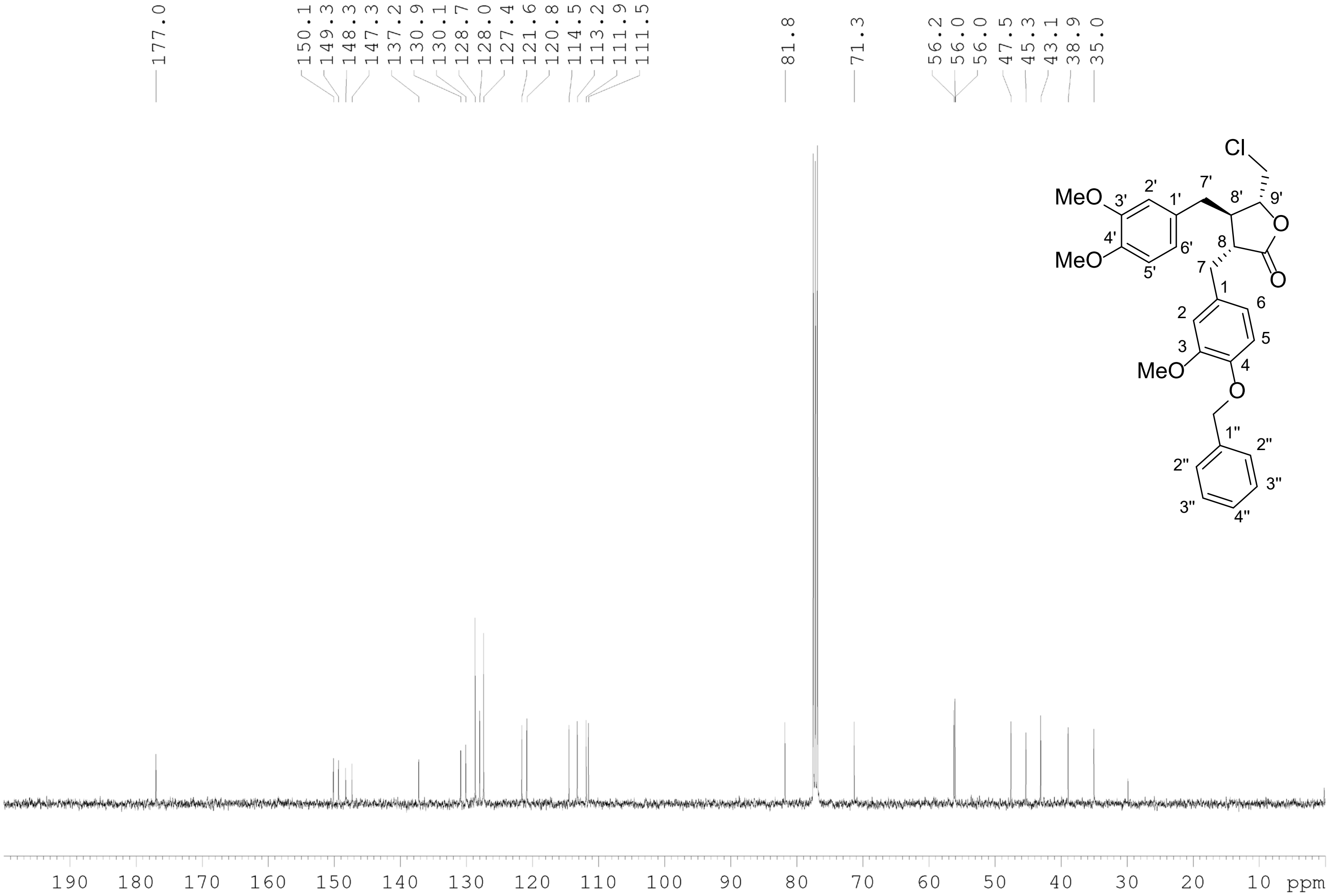

Figure S25. ^{13}C NMR spectrum of compound **30** in $CDCl_3$.

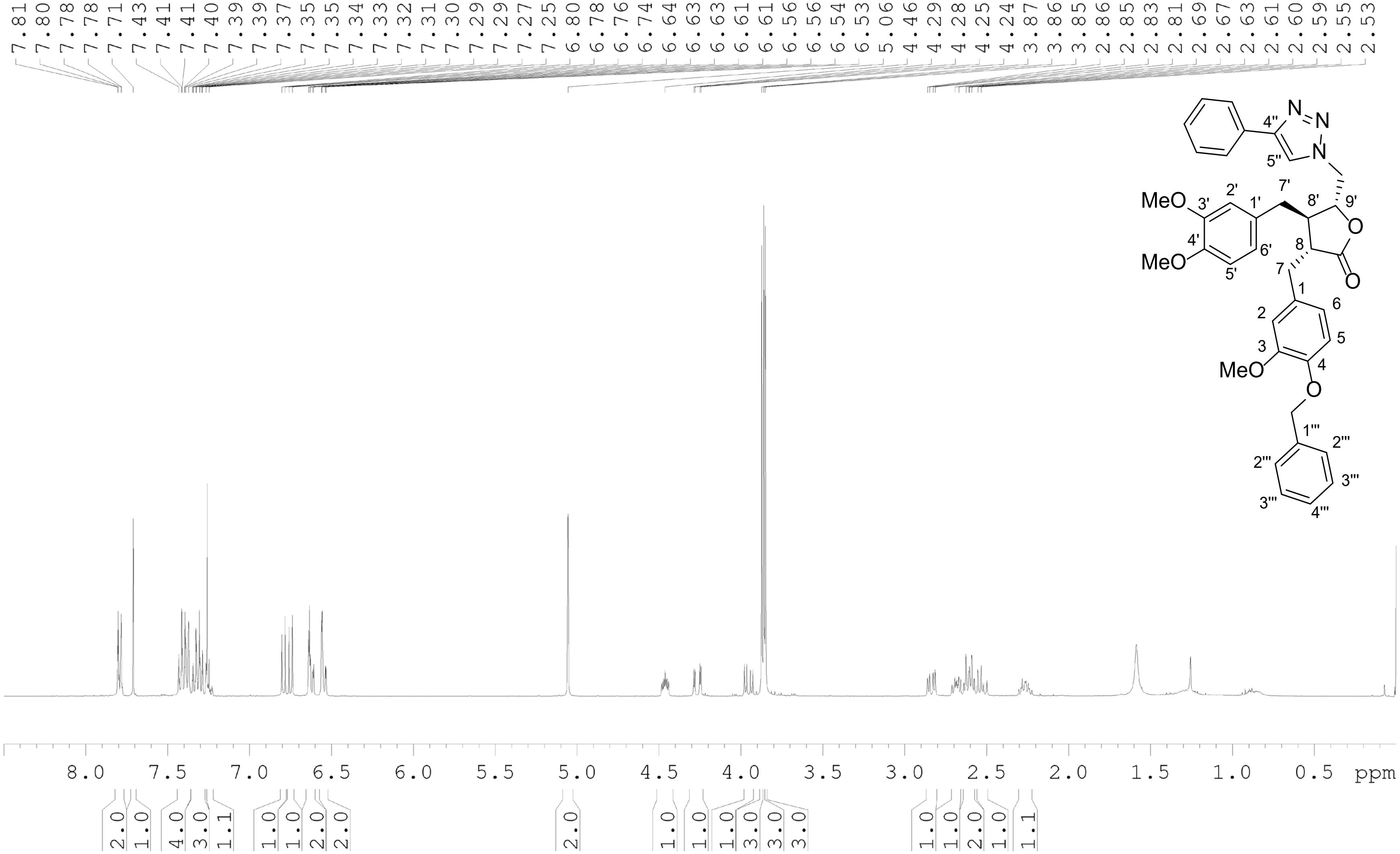

Figure S26. ^1H NMR spectrum of compound **32** in $CDCl_3$.

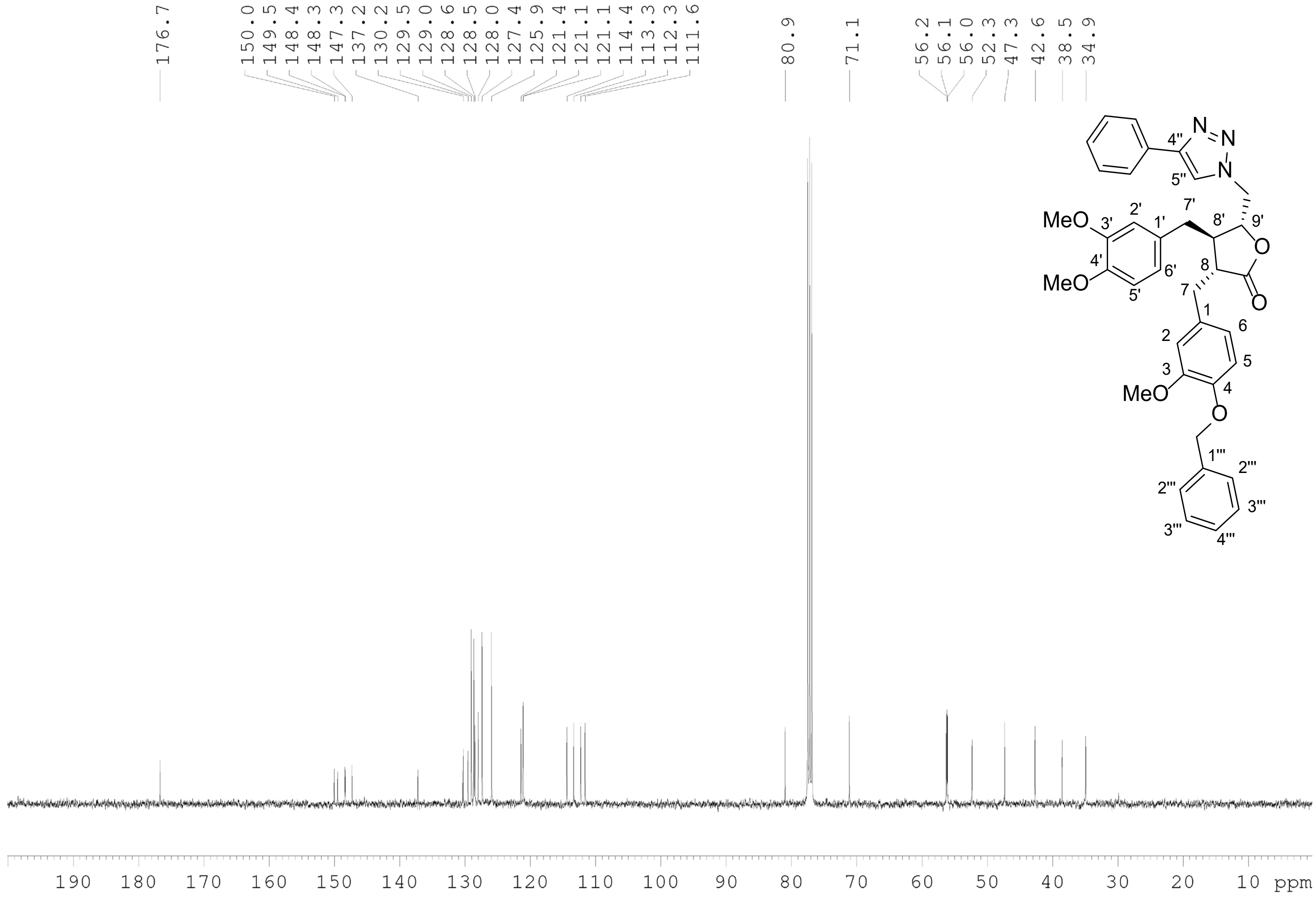

Figure S27. ^{13}C NMR spectrum of compound **32** in $CDCl_3$.

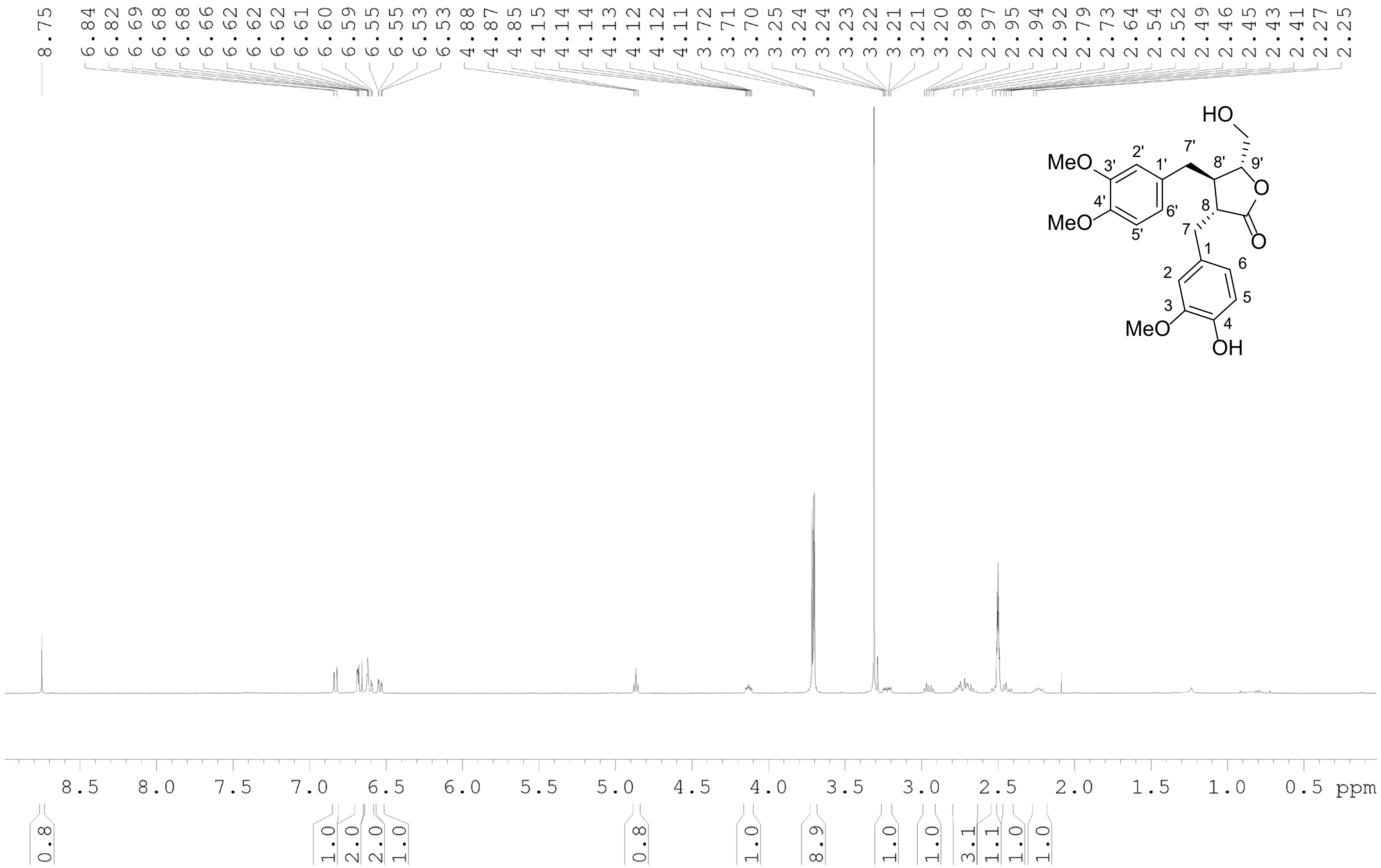

Figure S28. ^{1}H NMR spectrum of compound **20** in DMSO-d$_6$.

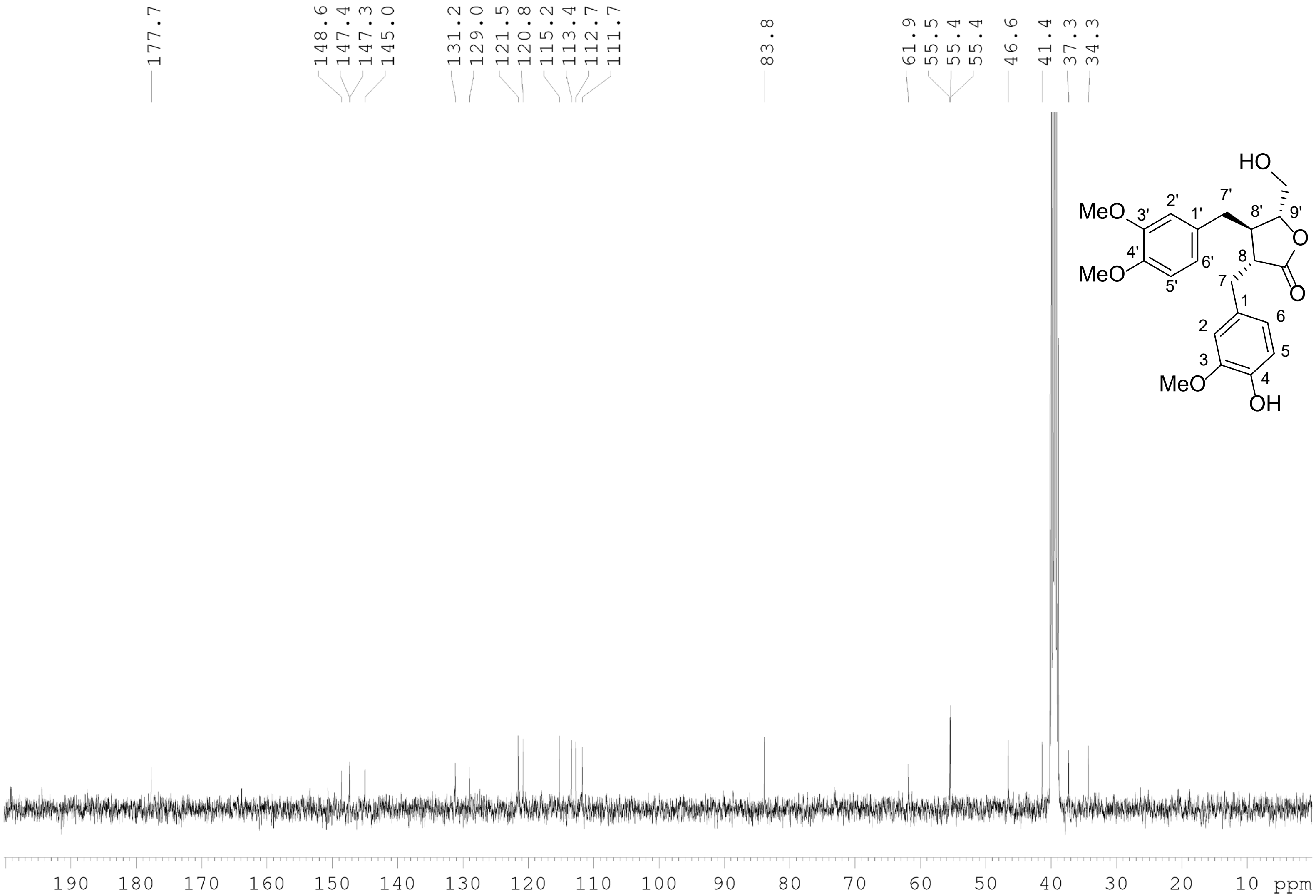

Figure S29. ^{13}C NMR spectrum of compound **20** in DMSO-d_6.

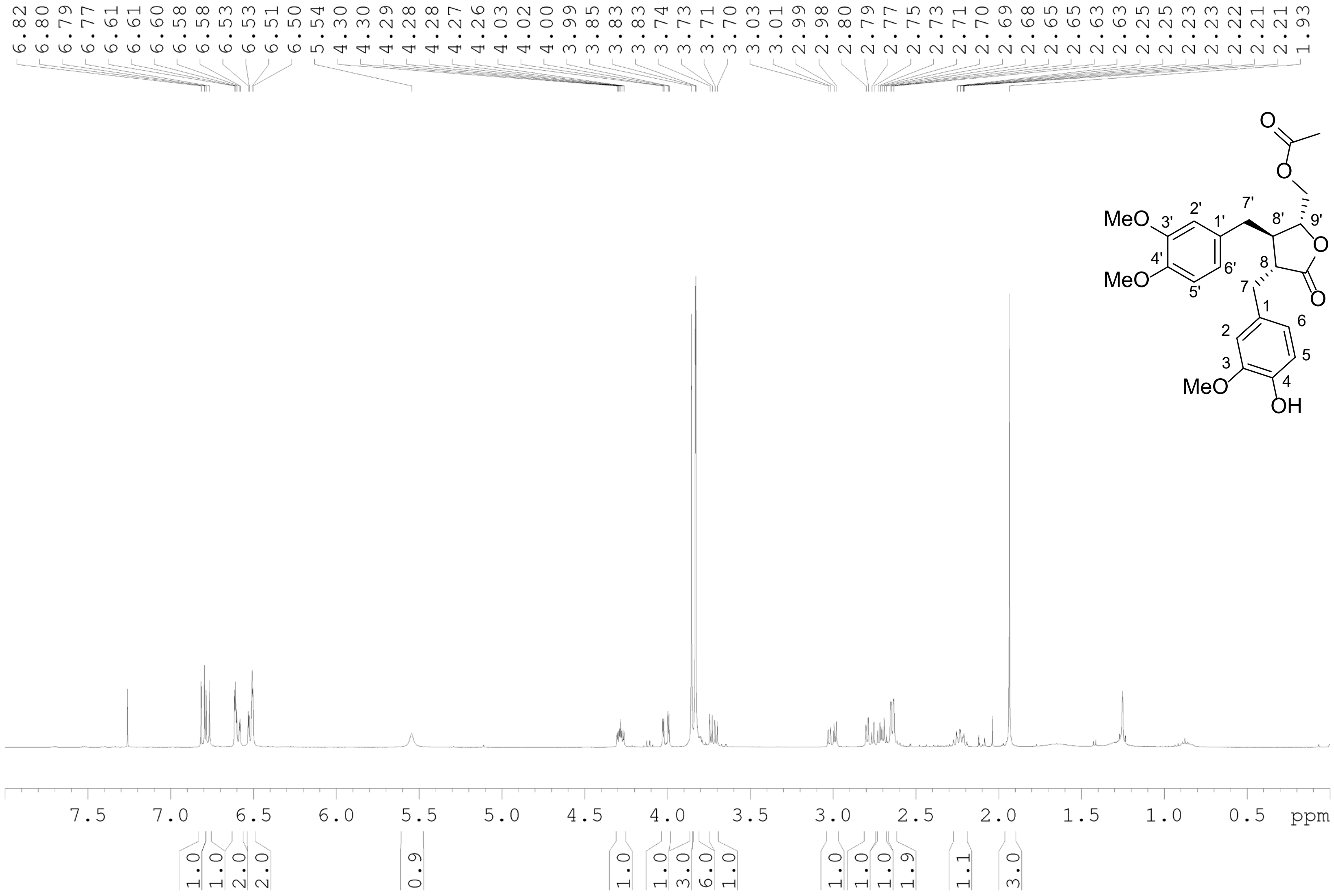

Figure S30. ^{1}H NMR spectrum of compound **25** in $CDCl_3$.

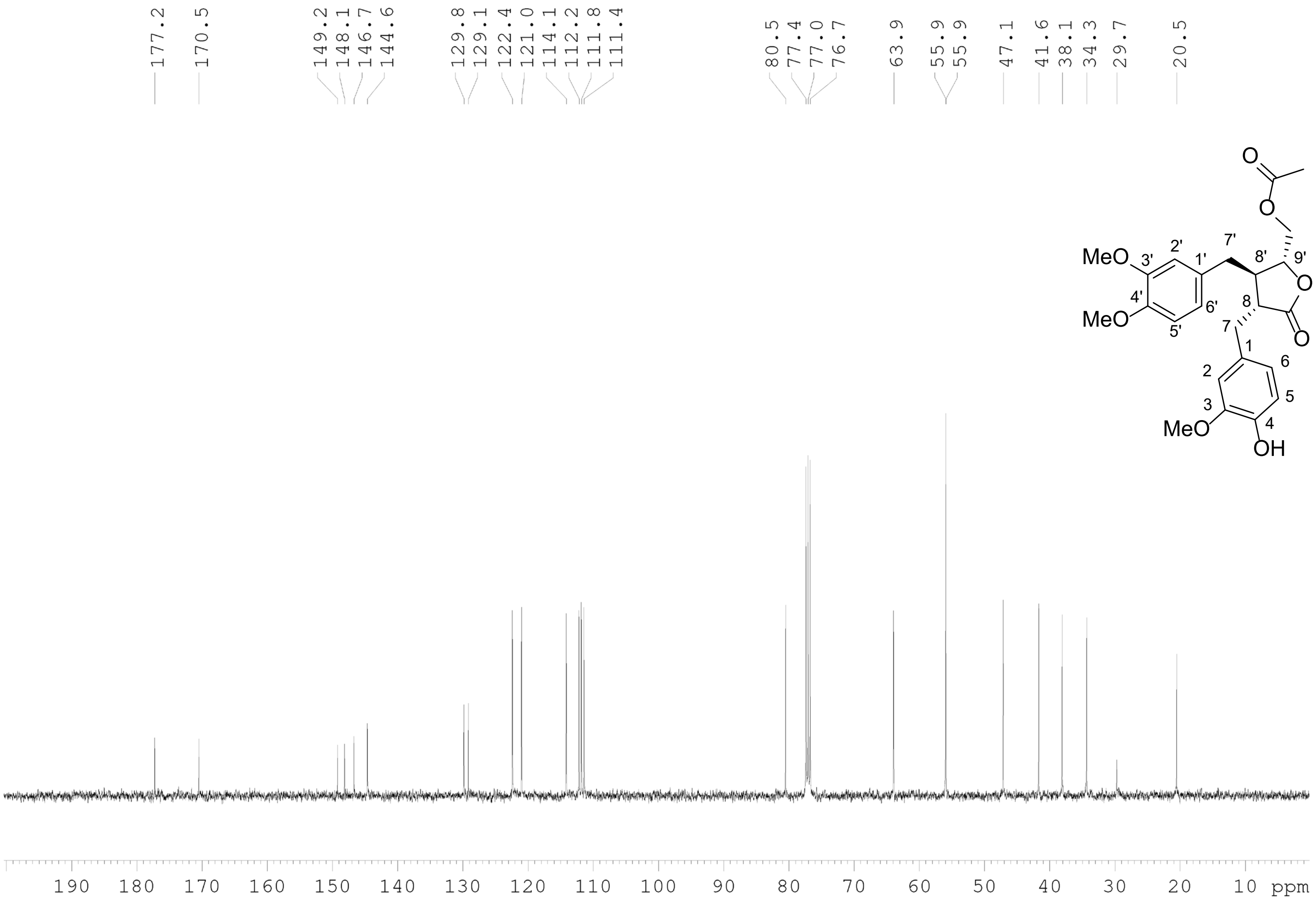

Figure S31. ^{13}C NMR spectrum of compound **25** in $CDCl_3$.

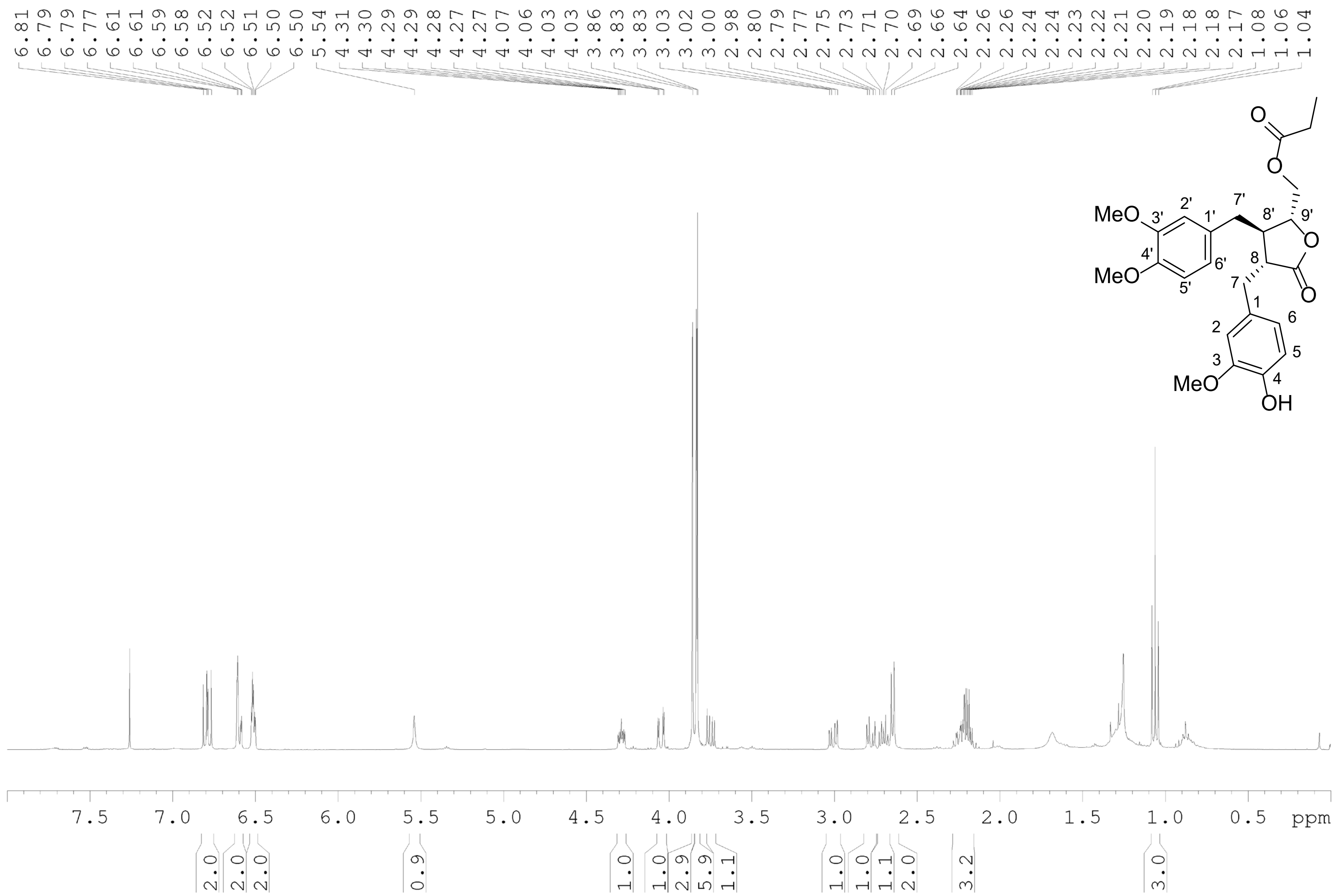

Figure S32. ^{1}H NMR spectrum of compound **26** in $CDCl_3$.

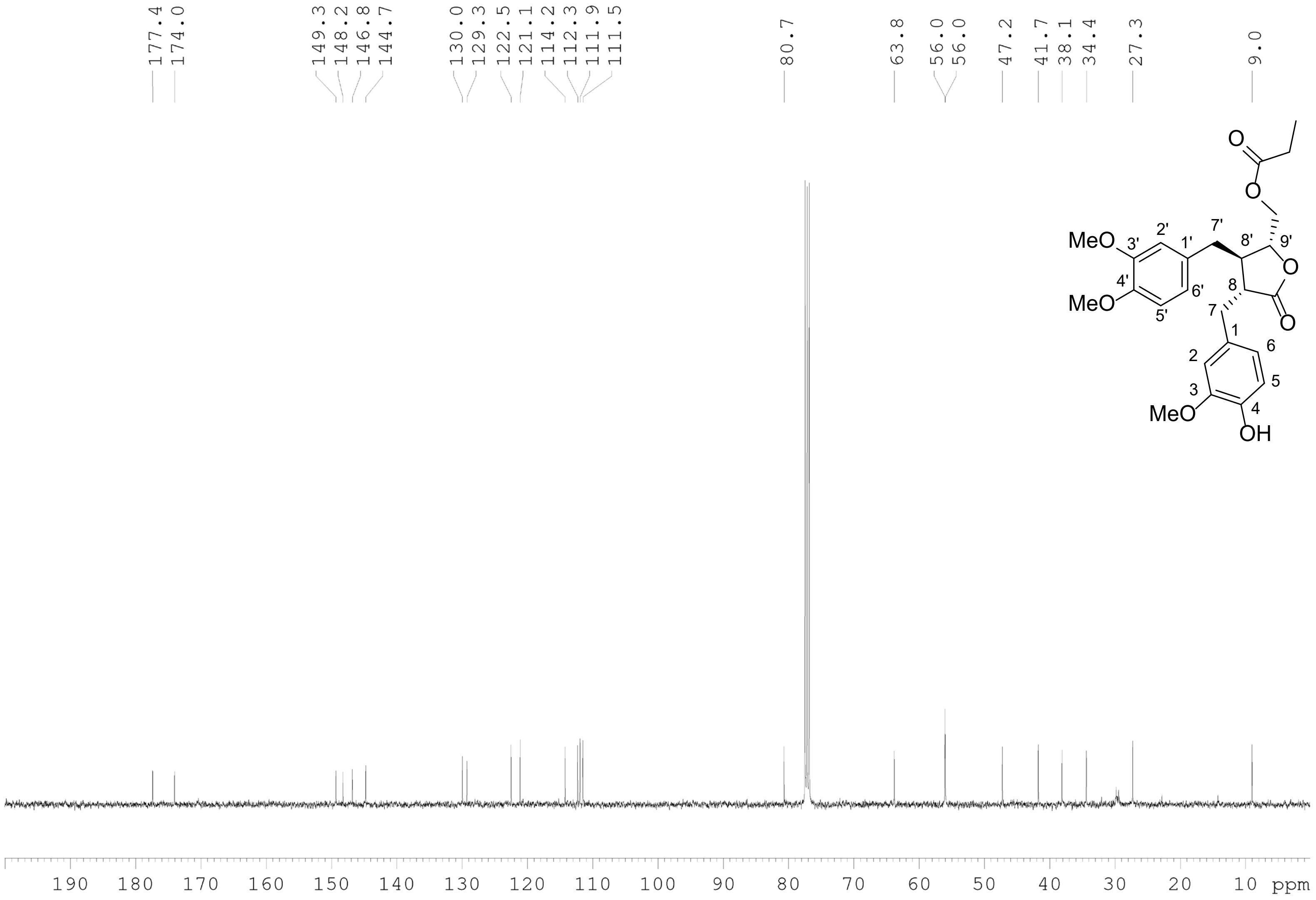

Figure S33. ^{13}C NMR spectrum of compound **26** in $CDCl_3$.

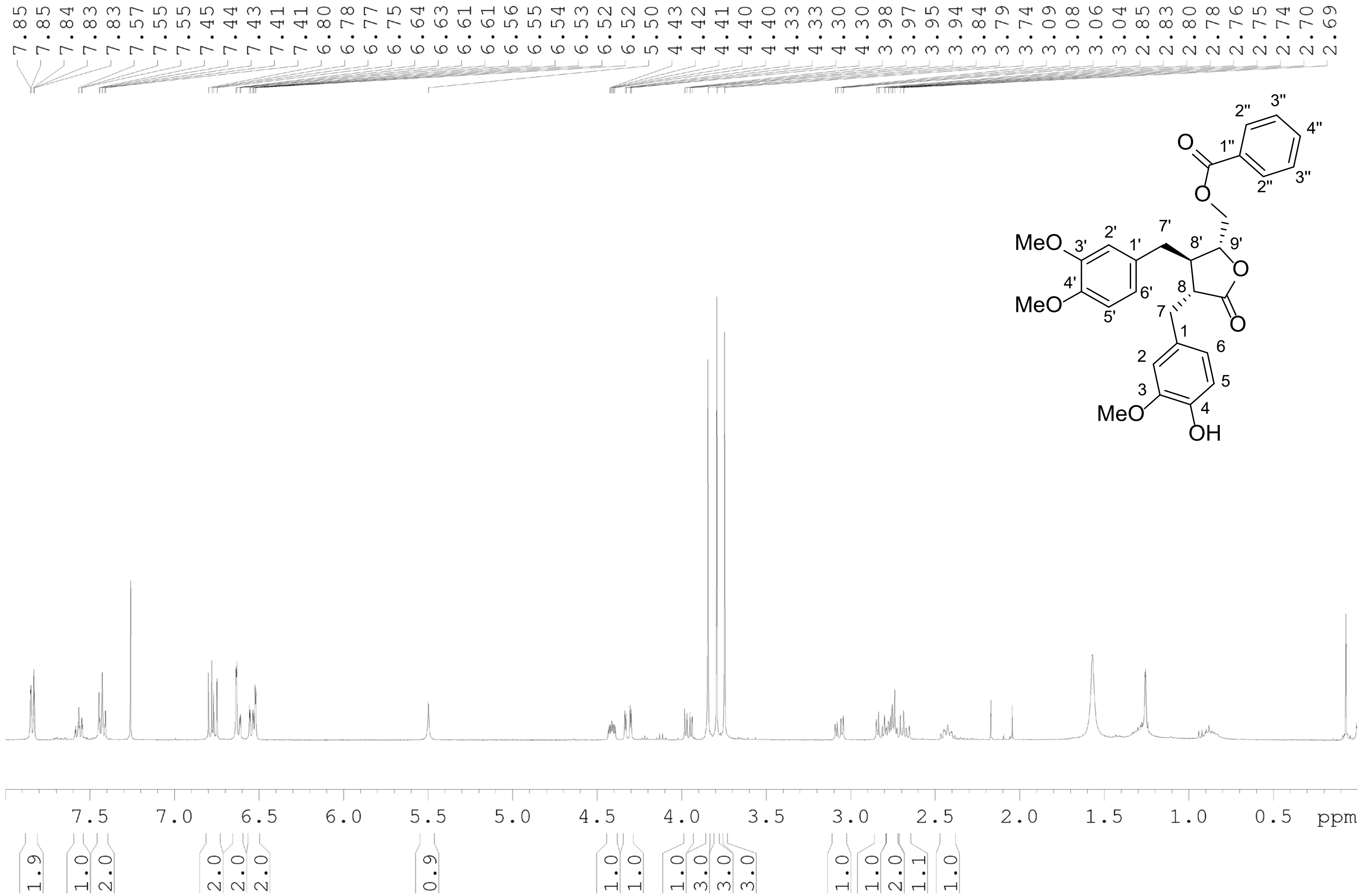

Figure S34. ^{1}H NMR spectrum of compound **27** in $CDCl_3$.

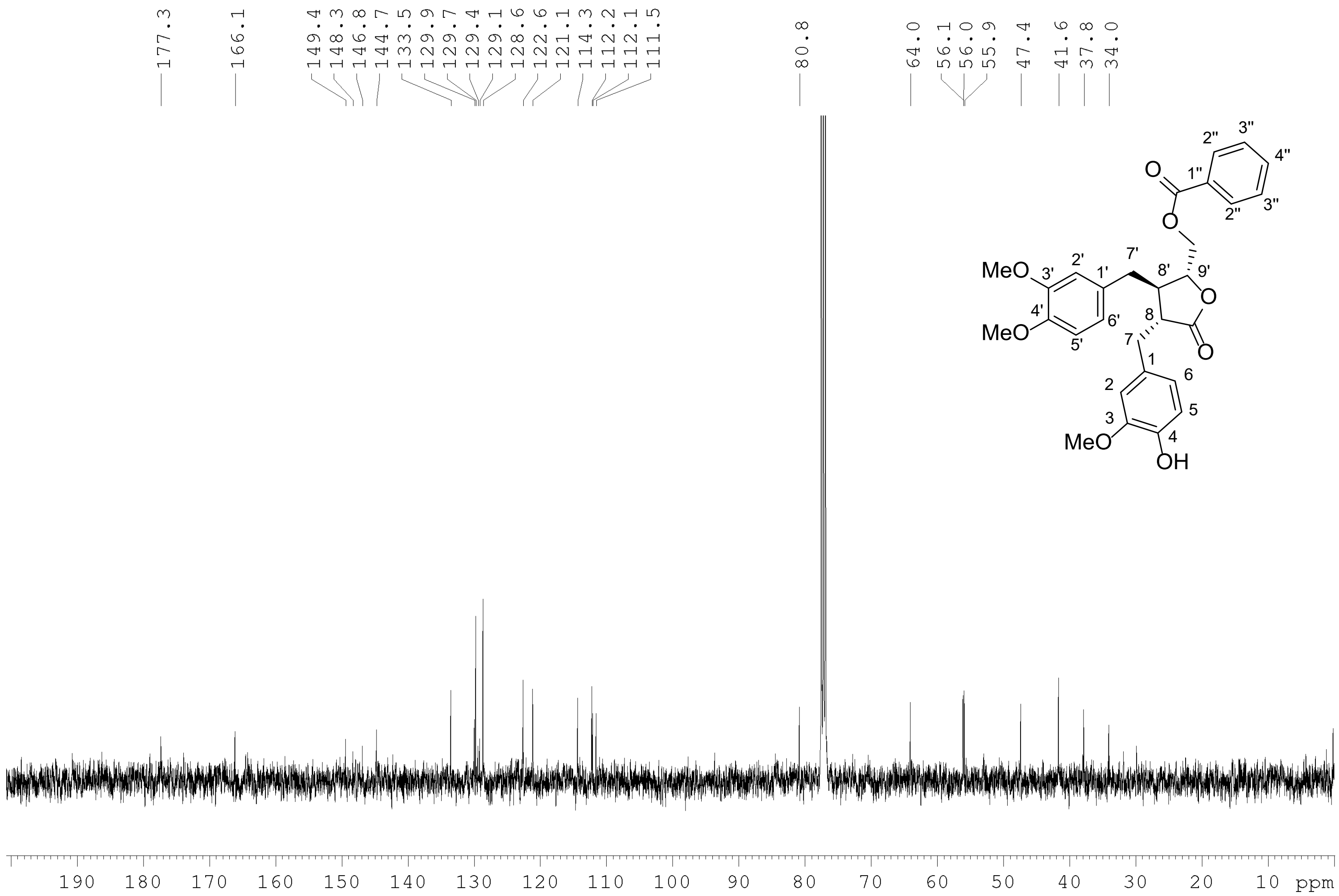

Figure S35. ^{13}C NMR spectrum of compound **27** in $CDCl_3$.

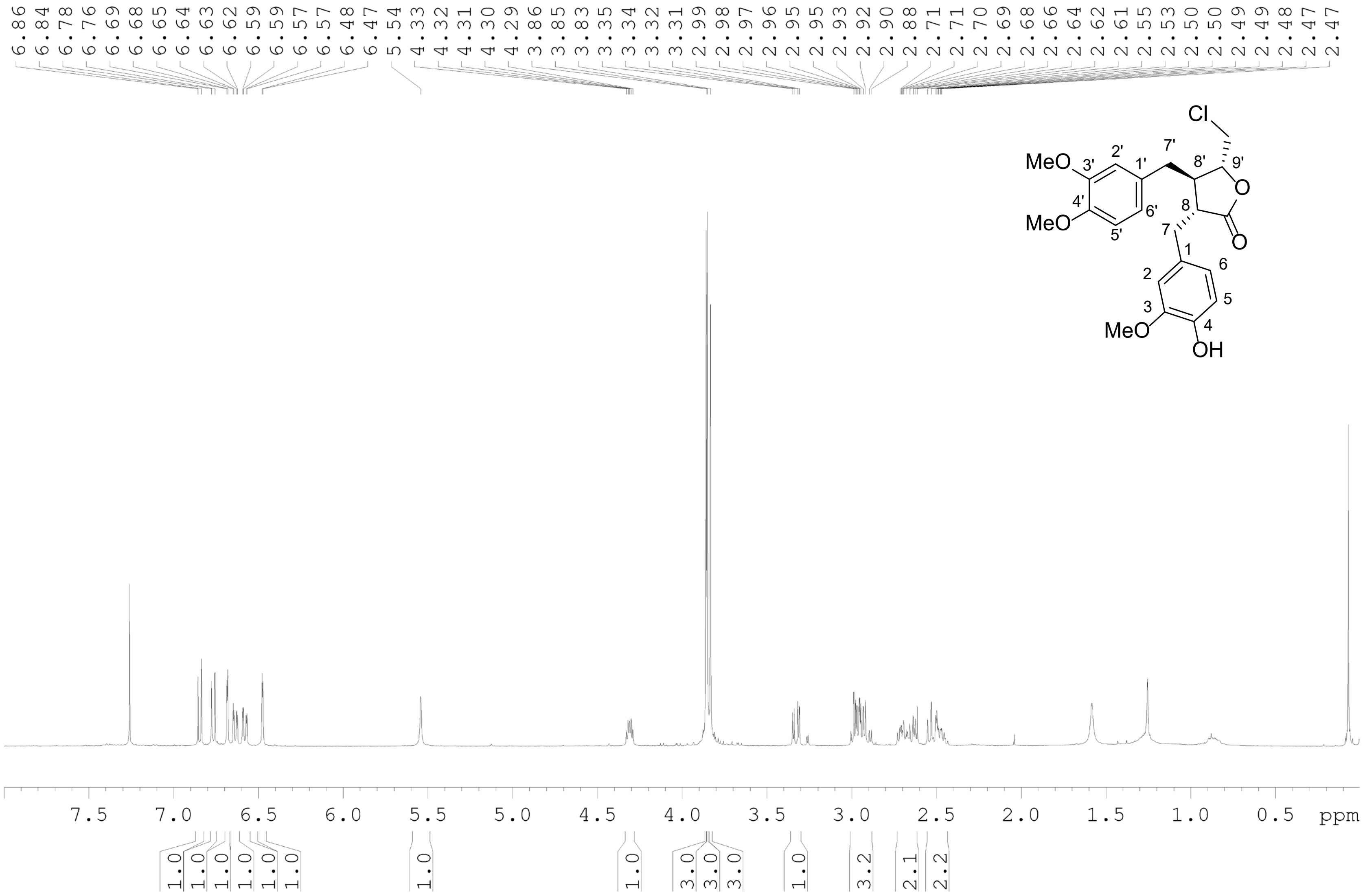

Figure S36. ^{1}H NMR spectrum of compound **33** in $CDCl_3$.

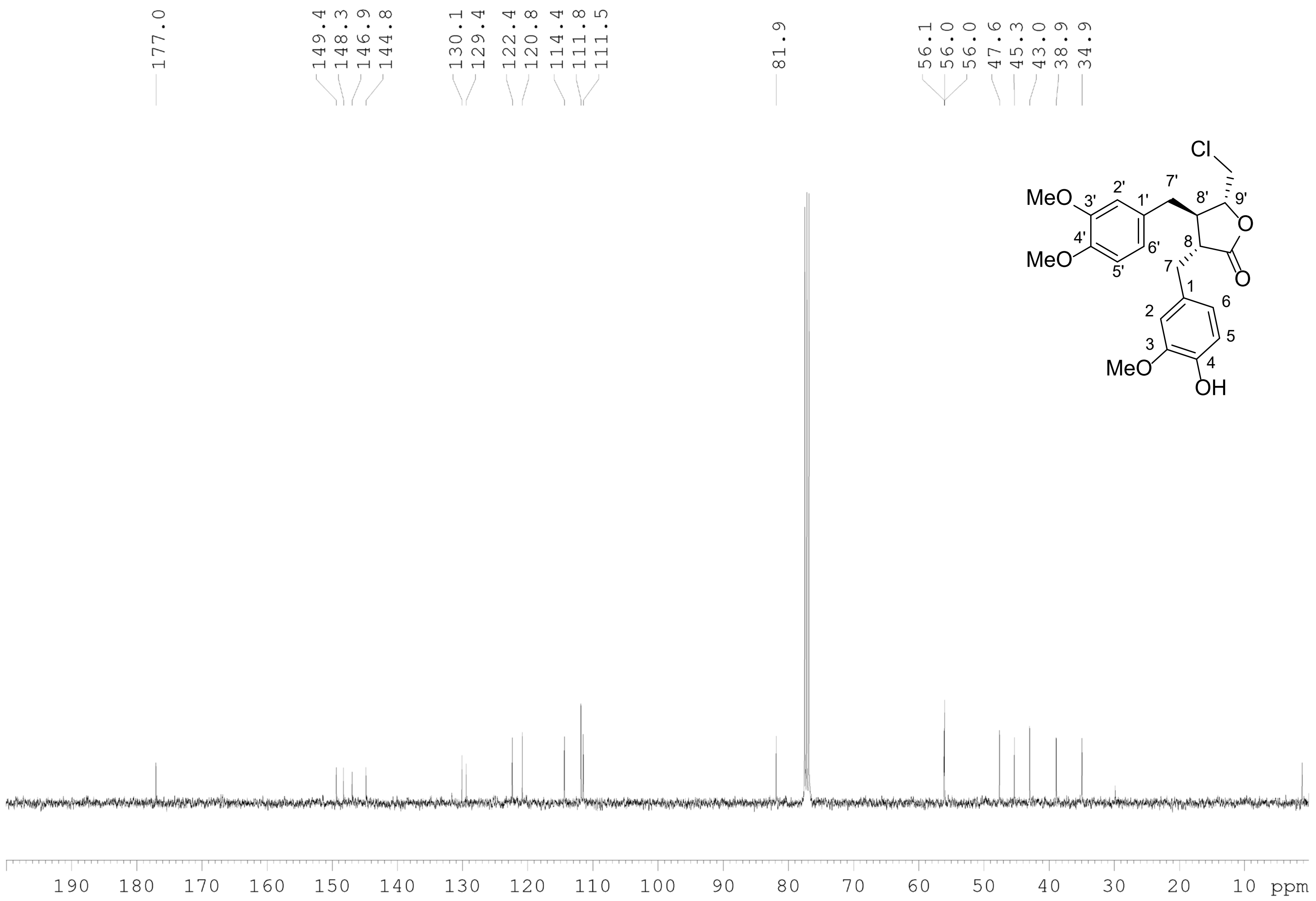

Figure S37. ^{13}C NMR spectrum of compound **33** in $CDCl_3$.

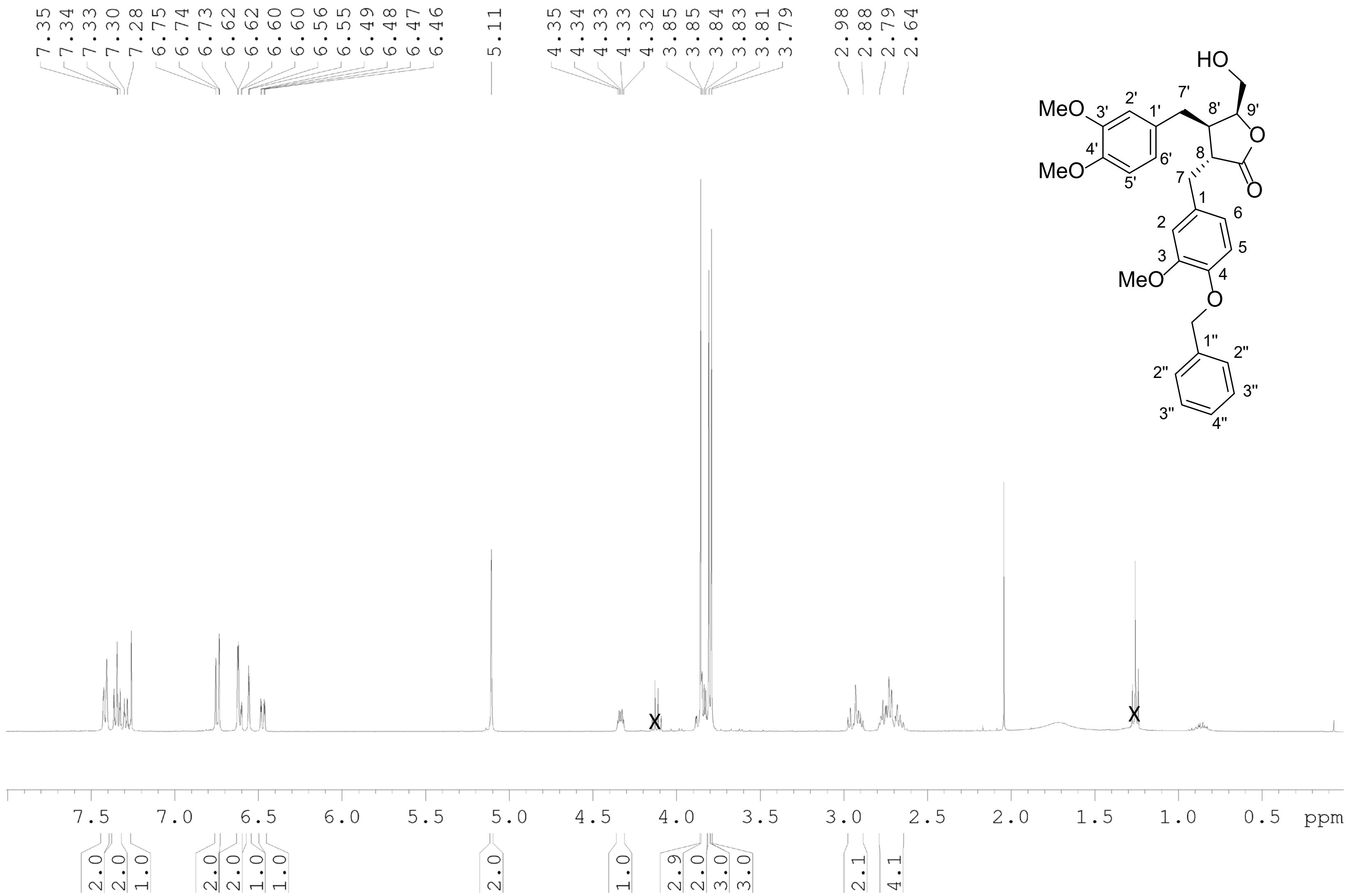

Figure S38. ^{1}H NMR spectrum of compound **18** in $CDCl_3$. Crosses indicate solvent signals.

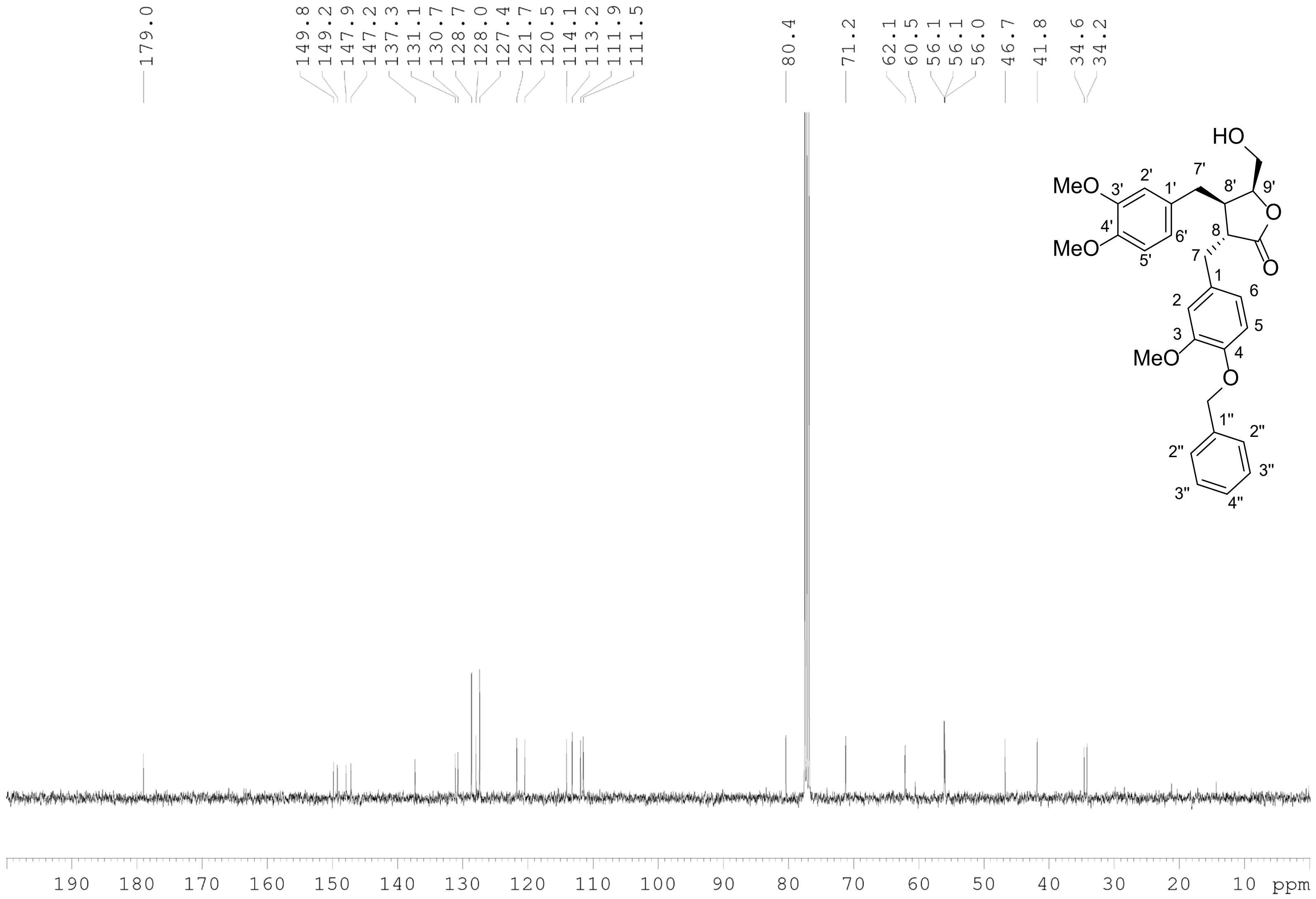

Figure S39. ^{13}C NMR spectrum of compound **18** in $CDCl_3$.

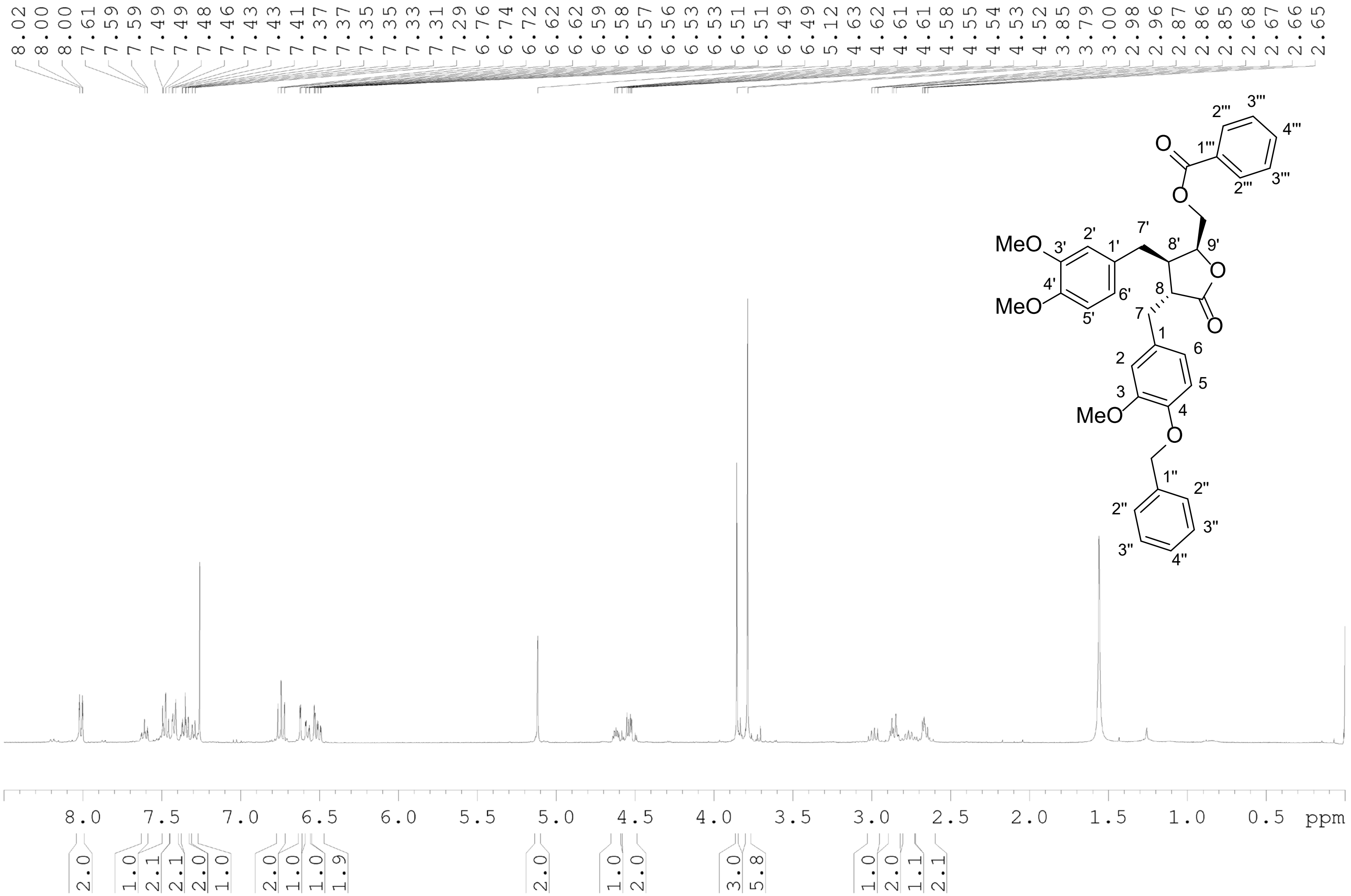

Figure S40. ^{1}H NMR spectrum of compound **24** in $CDCl_3$.

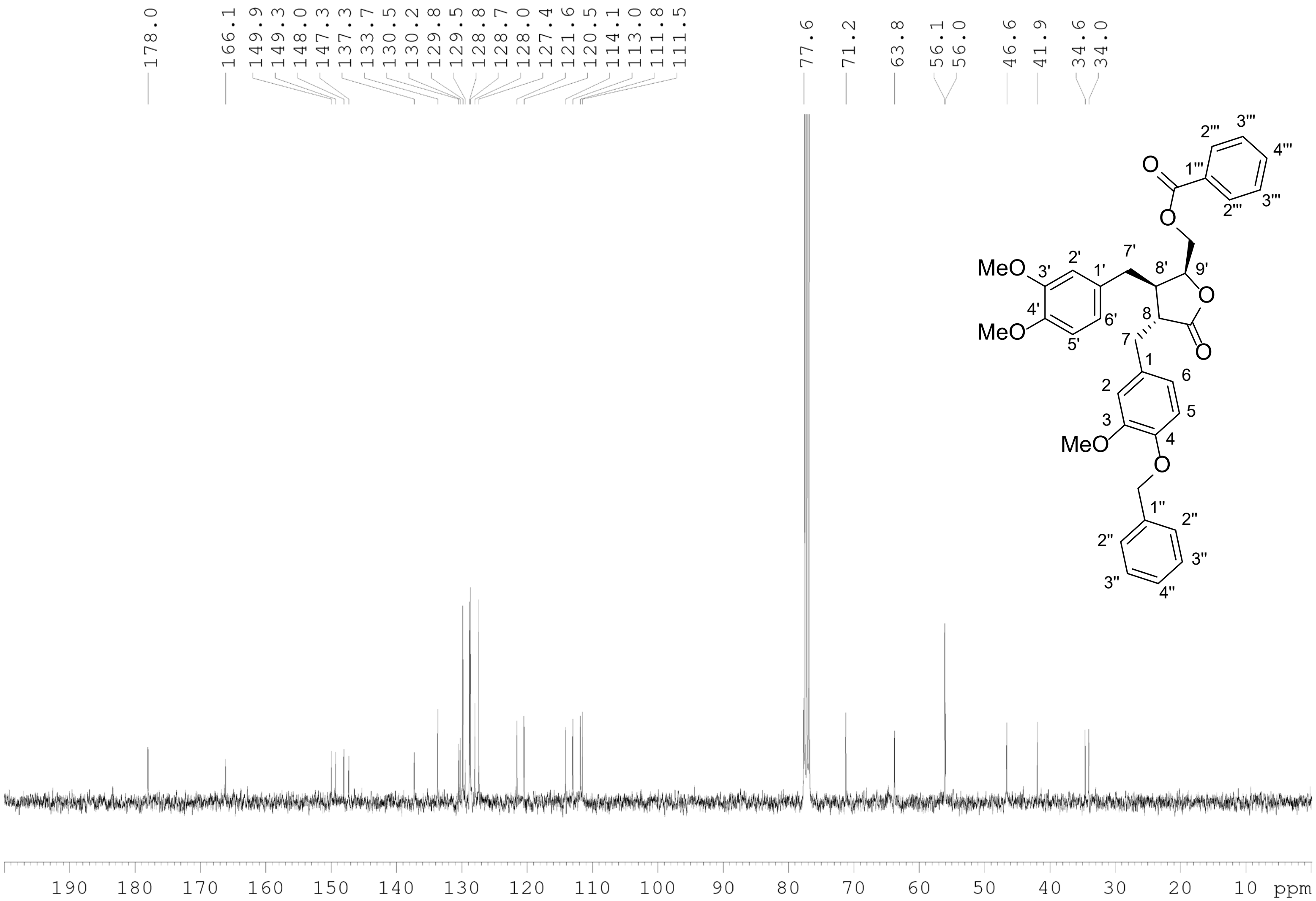

Figure S41. ^{13}C NMR spectrum of compound **24** in $CDCl_3$.

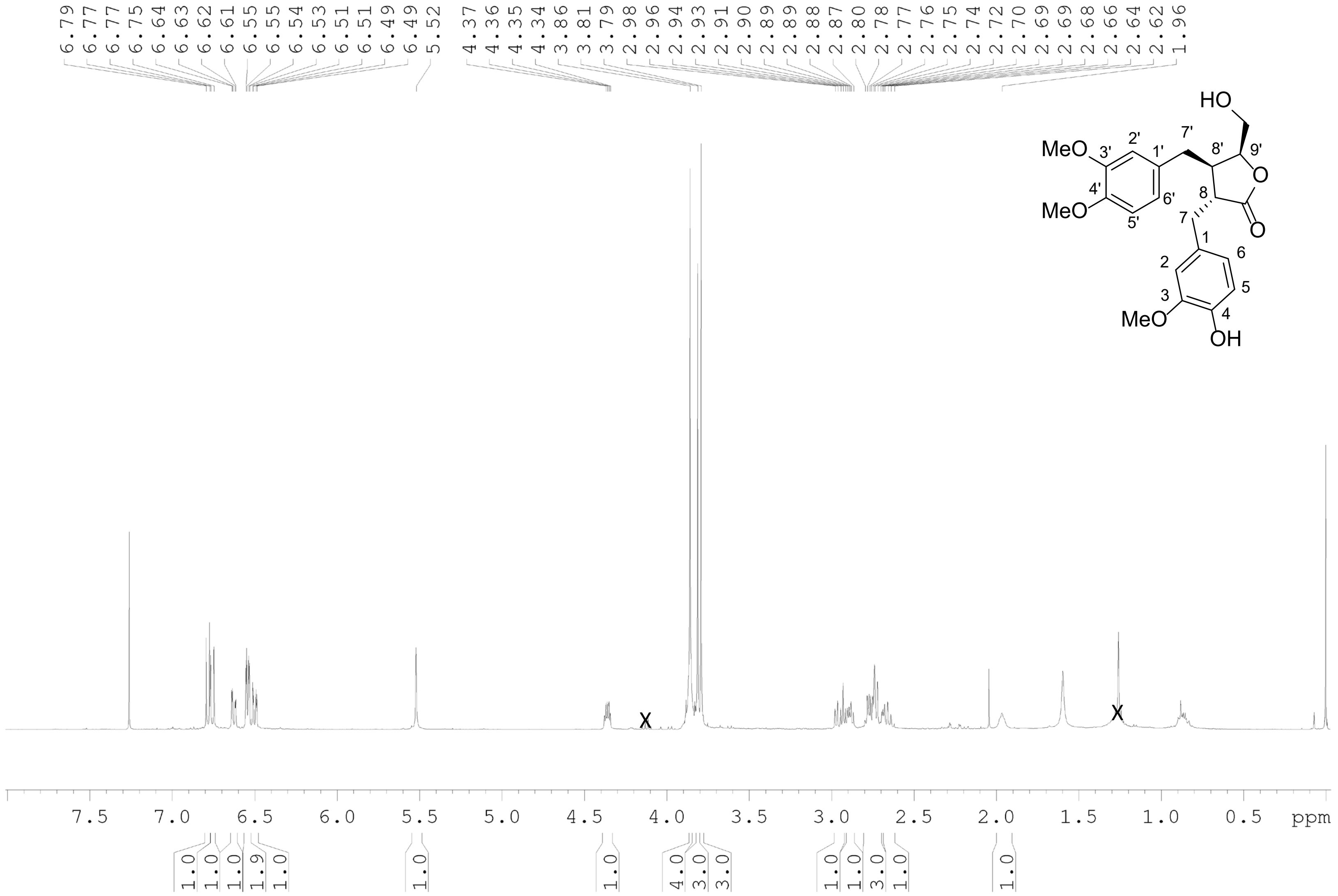

Figure S42. ^{1}H NMR spectrum of compound **19** in $CDCl_3$. Crosses indicate solvent signals.

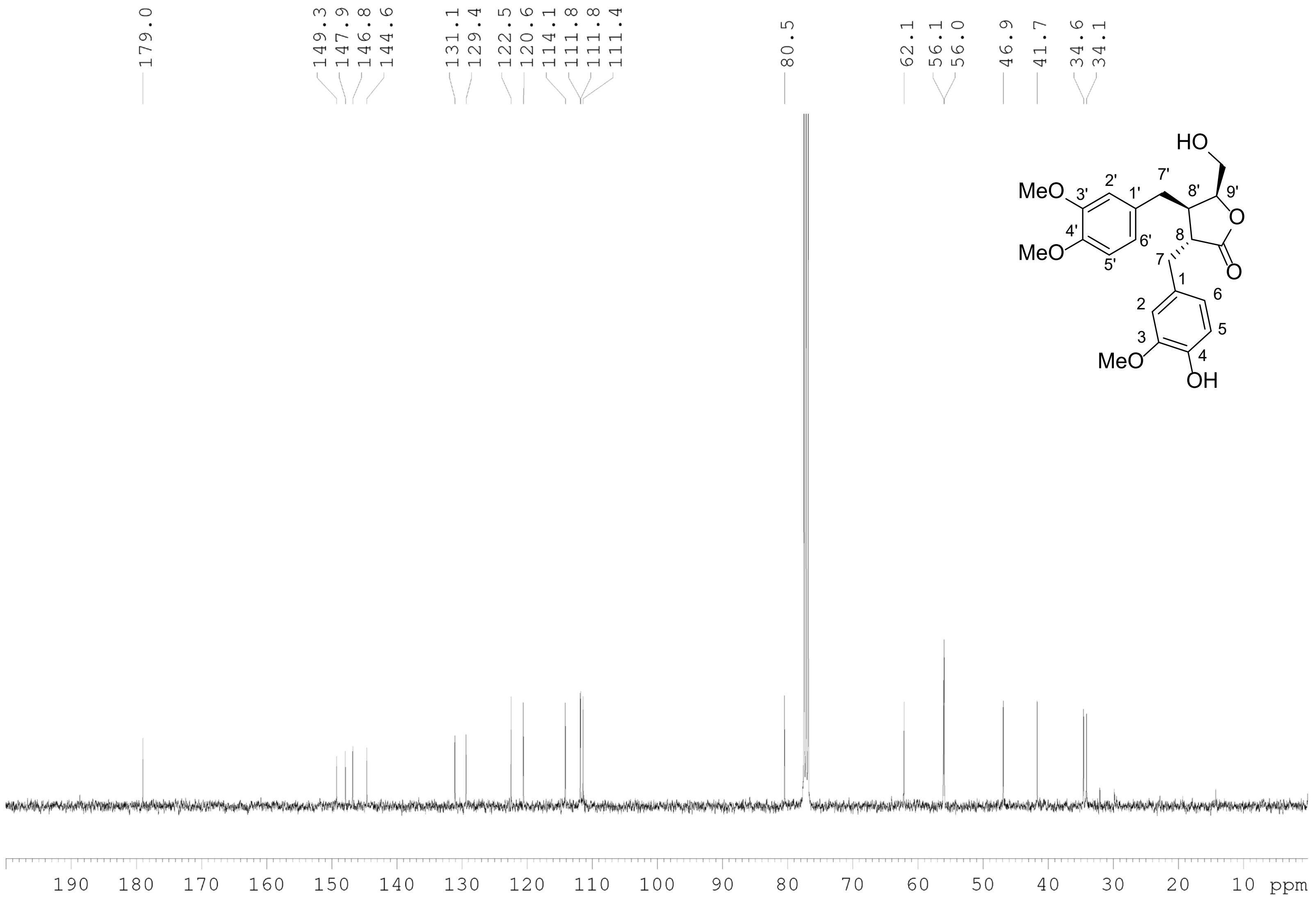

Figure S43. ^{13}C NMR spectrum of compound **19** in $CDCl_3$.

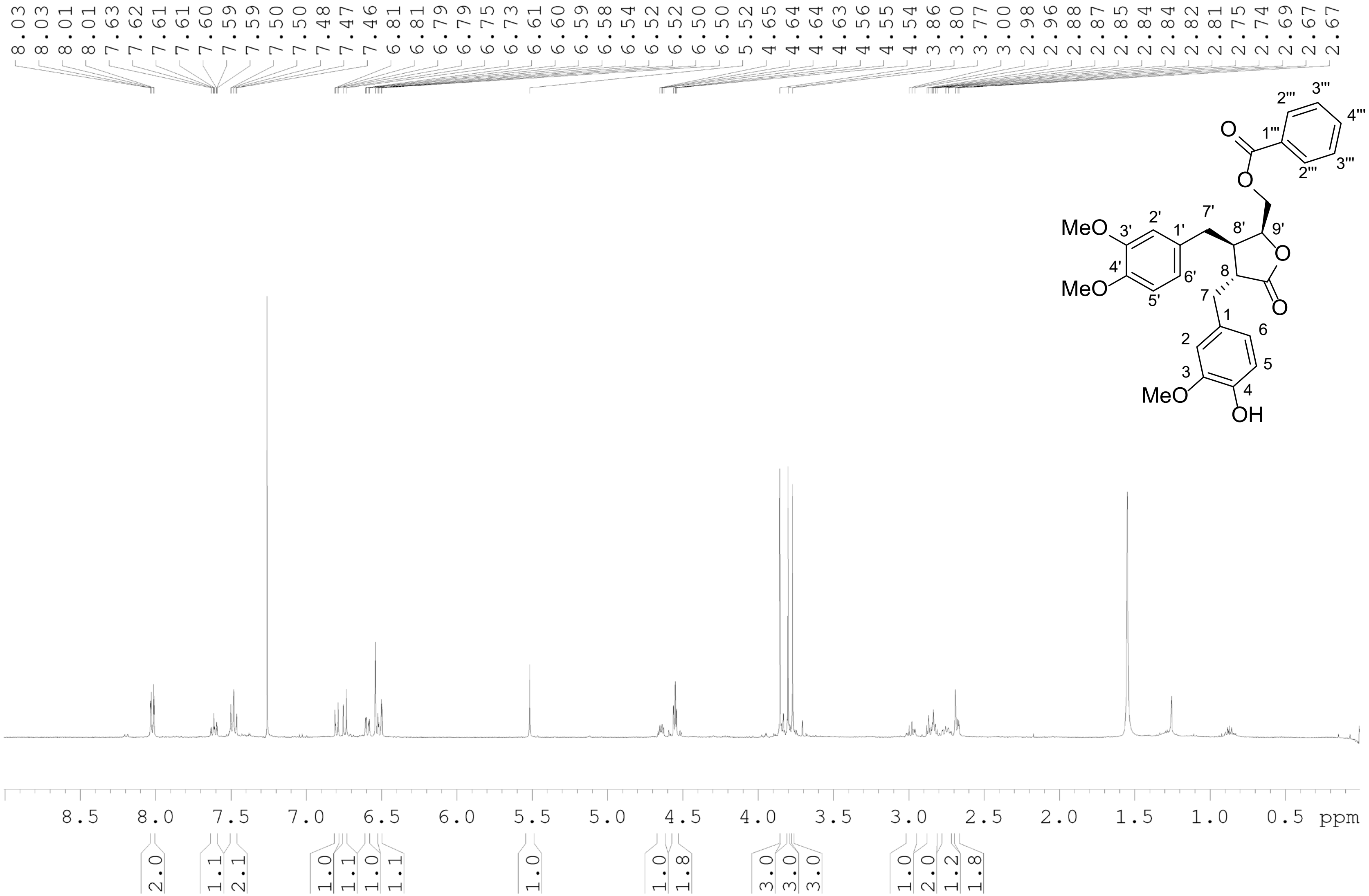

Figure S44. ^{1}H NMR spectrum of compound **28** in $CDCl_3$.

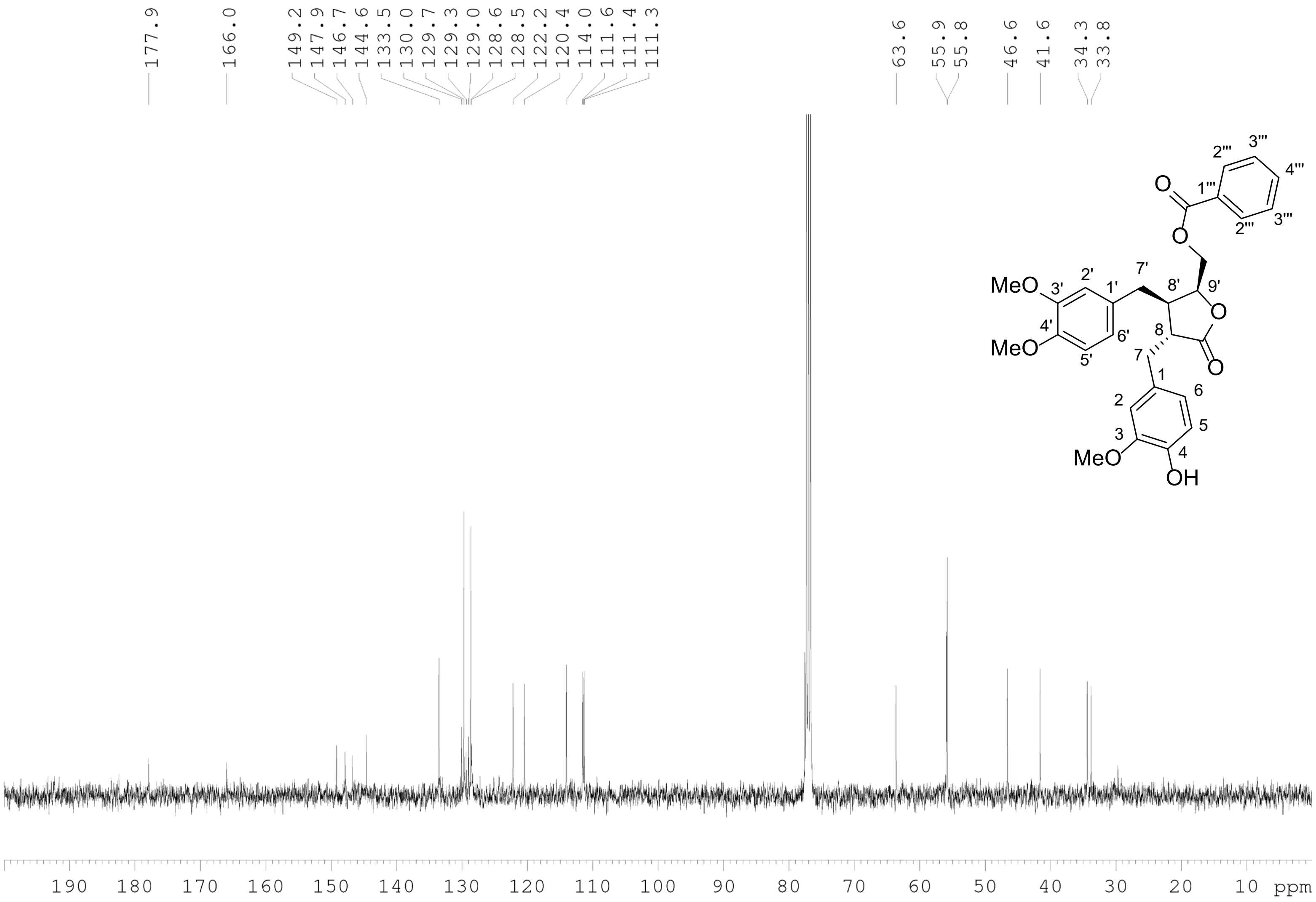

Figure S45. ^{13}C NMR spectrum of compound **28** in $CDCl_3$.

References

(1) Xia, Y.; Mo, Z.; Sun, L.; Zou, L.; Zhang, W.; Zhang, J.; Wang, L. First Total Synthesis of Quiquesetinerviusin A. *J. Chem. Res.* **2017**, *41* (5), 296–300. https://doi.org/10.3184/174751917X14931195075599.

(2) Hattori, H.; Mitsunaga, T.; Clive, D. L. J. Synthesis of Phenolic Components of Grains of Paradise. *Tetrahedron Letters* **2019**, *60* (30), 1989–1991. https://doi.org/10.1016/j.tetlet.2019.06.050.

(3) Purushotham Reddy, S.; Chinnababu, B.; Venkateswarlu, Y. First Stereoselective and Concise Synthesis of Rhoiptelol C. *Helvetica Chimica Acta* **2014**, *97* (7), 999–1003. https://doi.org/10.1002/hlca.201300390.

(4) Mane, B. B.; Kumbhar, D. D.; Waghmode, S. B. Enantioselective Total Synthesis of Ligraminol D and Ligraminol E. *Synlett* **2019**, *30* (20), 2285–2289. https://doi.org/10.1055/s-0039-1690249.

(5) Davidson, S. J.; Pilkington, L. I.; Dempsey-Hibbert, N. C.; El-Mohtadi, M.; Tang, S.; Wainwright, T.; Whitehead, K. A.; Barker, D. Modular Synthesis and Biological Investigation of 5-Hydroxymethyl Dibenzyl Butyrolactones and Related Lignans. *Molecules* **2018**, *23* (12), 3057. https://doi.org/10.3390/molecules23123057.

(6) Kikuzaki, H.; Hara, S.; Kawai, Y.; Nakatani, N. Antioxidative Phenylpropanoids from Berries of Pimenta Dioica. *Phytochemistry* **1999**, *52* (7), 1307–1312. https://doi.org/10.1016/S0031-9422(99)00406-9.

(7) Davies, S. G.; Goddard, E. C.; Roberts, P. M.; Russell, A. J.; Smith, A. D.; Thomson, J. E.; Withey, J. M. Strategies for the Construction of Morphinan Alkaloid AB-Rings: Regioselective Friedel-Crafts-Type Cyclisations of γ-Aryl-β-Benzoylamido Acids with Asymmetrically Substituted γ-Aryl Rings. *Tetrahedron Asymmetry* **2016**, *27* (6), 274–284. https://doi.org/10.1016/j.tetasy.2016.02.010.

(8) Sun, R.; Song, W.; Ma, C.; Zhang, H.; Yu, X. Titanium(IV) Chloride-Mediated Stereoselective α-Alkylidenation to Efficiently Assemble Multisubstituted 1,3-Dienes. *Adv. Synth. Catal.* **2016**, *358* (24), 3977–3982. https://doi.org/10.1002/adsc.201601025.

(9) Hryniewicka, A.; Misztalewska, I.; Czajkowska-Szczykowska, D.; Urbańczyk-Lipkowska, Z.; Morzycki, J. W.; Witkowski, S. New Olefin Metathesis Catalysts Bearing Polyether Clamp in N-Heterocyclic Carbenes Ligands. *Tetrahedron* **2014**, *70* (38), 6810–6816. https://doi.org/10.1016/j.tet.2014.07.056.

(10) Parpal, F.; Pandolfi, E.; Heguaburu, V. (π-Allyl)Palladium Coupling of 2-(Tributylstannyl)Cyclopent-2-Enone for the Synthesis of Jasmonoid Analogs. *Tetrahedron Lett.* **2017**, *58* (20), 1965–1968. https://doi.org/10.1016/j.tetlet.2017.04.018.

(11) Dittrich, N.; Pilkington, L. I.; Leung, E.; Barker, D. Synthesis of N-Benzyl-Des-D-Ring Lamellarin K via an Acyl-Claisen/Paal-Knorr Approach. *Tetrahedron* **2017**, *73* (14), 1881–1894. https://doi.org/10.1016/j.tet.2017.02.044.
